# PFAS Exposure and Postoperative Weight Regain in Adolescents After Bariatric Surgery: Findings From the Teen‐LABS Study

**DOI:** 10.1002/oby.70009

**Published:** 2025-08-14

**Authors:** Brittney O. Baumert, Elizabeth Costello, Zhenjiang Li, Justin R. Ryder, Thomas Inge, Todd Jenkins, Stephanie Sisley, Stavra A. Xanthakos, Douglas I. Walker, Nikos Stratakis, Damaskini Valvi, Scott M. Bartell, Angela L. Slitt, Rohit Kohli, Sarah Rock, Michele A. La Merrill, Sandrah P. Eckel, Max T. Aung, Rob McConnell, David V. Conti, Lida Chatzi

**Affiliations:** ^1^ Department of Population and Public Health Sciences Keck School of Medicine, University of Southern California Los Angeles California USA; ^2^ Department of Surgery Northwestern University Feinberg School of Medicine Chicago Illinois USA; ^3^ Ann & Robert H. Lurie Children's Hospital of Chicago Chicago Illinois USA; ^4^ Department of Pediatrics, Cincinnati Children's Hospital Medical Center University of Cincinnati College of Medicine Cincinnati Ohio USA; ^5^ Department of Pediatrics Baylor College of Medicine Houston Texas USA; ^6^ Gangarosa Department of Environmental Health Rollins School of Public Health Atlanta Georgia USA; ^7^ Barcelona Institute for Global Health, ISGlobal Barcelona Spain; ^8^ Department of Environmental Medicine and Climate Science Icahn School of Medicine at Mount Sinai New York New York USA; ^9^ Department of Environmental and Occupational Health University of California, Irvine Irvine California USA; ^10^ College of Pharmacy The University of Rhode Island Kingston Rhode Island USA; ^11^ Division of Gastroenterology, Hepatology and Nutrition Children's Hospital Los Angeles Los Angeles California USA; ^12^ Department of Environmental Toxicology University of California, Davis Davis California USA

**Keywords:** adolescents, bariatric surgery, BMI, metabolic health, PFAS, weight regain

## Abstract

**Objective:**

Weight regain following bariatric surgery remains a clinical challenge, with limited understanding of contributing environmental factors. Per‐ and polyfluoroalkyl substances (PFAS), persistent chemicals linked to metabolic dysfunction, may influence long‐term weight trajectories. This study aimed to evaluate associations between PFAS exposure and changes in BMI, percent weight loss, and waist circumference among adolescents after bariatric surgery.

**Methods:**

We included 186 adolescents (mean age: 17.1 years; 76.3% female; 72.0% White) from the Teen‐Longitudinal Assessment of Bariatric Surgery (Teen‐LABS) cohort who underwent surgery between 2007 and 2012. Anthropometric measurements were collected at baseline and 6, 12, 36, and 60 months post surgery. Presurgical plasma concentrations of seven PFAS were measured using liquid chromatography–tandem mass spectrometry. Associations were estimated using linear mixed‐effects models and quantile g‐computation.

**Results:**

Higher concentrations of PFOS, PFHxS, and PFHpS were associated with greater BMI regain, reduced percent weight loss, and increased waist circumference from 1 to 5 years post surgery. At PFOS concentrations of 1.45 to 2.94 log_2_ ng/mL, annual BMI regain increased from 1.34 to 1.84 kg/m^2^ (*p* = 0.0497). Mixture analyses confirmed cumulative PFAS effects, with sulfonic acids showing the strongest associations.

**Conclusions:**

PFAS exposure was associated with weight regain after bariatric surgery in adolescents, potentially undermining long‐term metabolic benefits.

**Trial Registration:**

ClinicalTrials.gov identifier NCT00474318

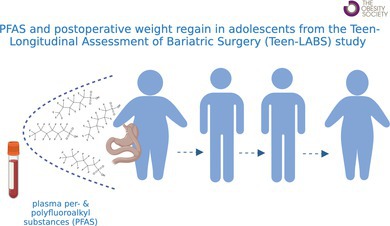

## Graphical abstract

Elevated baseline PFAS levels, particularly PFOS, PFHxS, and PFHpS, were associated with greater BMI regain, reduced weight loss, and increased waist circumference in adolescents after bariatric surgery. These sulfonic acid PFAS showed a dose‐dependent effect on BMI regain. Mixture analyses confirmed their cumulative impact, with sulfonic acids having the strongest associations.

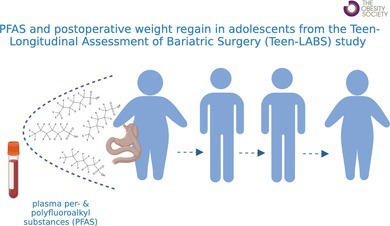

## 1 Introduction

Obesity remains a significant public health challenge, particularly in pediatric populations, for which severe obesity is associated with an increased risk of developing long‐term adverse health outcomes, including cardiovascular disease, type 2 diabetes, and reduced quality of life [1, 2]. In adolescents, the prevalence of severe obesity has risen dramatically over the past several decades, underscoring the urgent need for effective interventions [1, 3]. Bariatric surgery has emerged as an effective treatment for severe obesity in this age group, leading to substantial initial weight loss and significant improvements in obesity‐related comorbidities [4, 5]. However, one of the major challenges following bariatric surgery is the tendency for many patients to regain weight after the first postoperative year [6]. Weight regain not only compromises the health benefits of surgery but also poses psychological and physical challenges for adolescents and their families [6, 7]. Despite its clinical significance, the factors contributing to postoperative weight regain remain poorly understood.

Evidence suggests that environmental exposures may play an important role in weight regulation and metabolic health [8, 9]. Among these, per‐ and polyfluoroalkyl substances (PFAS) have gained attention due to their widespread use, persistence in the environment, and endocrine‐disrupting effects [9]. PFAS are synthetic chemicals commonly used in industrial and consumer products such as nonstick cookware, food packaging, and water‐ and stain‐repellent textiles due to their water‐ and grease‐resistant properties [9, 10]. These chemicals are highly stable, leading to their accumulation in the environment and the human body, where they have a long biological half‐life [9, 11]. Consequently, PFAS exposure is nearly ubiquitous, with measurable concentrations detected in the blood of the majority of the global population [9].

Numerous studies have identified associations between increased exposure to PFAS and metabolic dysregulation, including disruptions in lipid metabolism, glucose homeostasis, and alterations in body weight. In adults, higher PFAS concentrations have been associated with reduced weight loss during dietary interventions and greater weight regain after weight‐loss programs [12, 13]. Experimental and mechanistic studies suggest that PFAS may impair energy balance by altering adipogenesis, mitochondrial function, and hormone regulation of appetite and metabolism [14, 15]. Despite this growing body of evidence, the impact of PFAS exposure on weight outcomes in adolescents, particularly those undergoing bariatric surgery, remains unexplored.

Among PFAS subtypes, sulfonic acid‐containing compounds (PFSAs) such as PFOS and PFHxS may exert greater metabolic impact than carboxylic acid‐containing compounds (PFCAs) like PFOA and PFNA [16]. PFSAs are characterized by longer biological half‐lives, greater bioaccumulation potential, and stronger binding affinity to proteins involved in lipid transport and metabolism, including liver fatty acid binding protein (L‐FABP) and peroxisome proliferator‐activated receptors (PPARs) [17, 18, 19]. These properties may enhance their ability to disrupt key metabolic pathways and energy homeostasis, making them particularly relevant for studying postoperative weight dynamics. Despite this mechanistic evidence, the differential effects of PFAS subtypes on weight outcomes, particularly in adolescents, remain largely unexamined.

While several studies have documented the adverse metabolic effects of PFAS in adult populations, including reduced weight loss and increased weight regain following dietary interventions, the extent to which these findings apply to adolescents is unknown [12, 13]. Adolescents differ from adults in key developmental, hormonal, and metabolic processes, which may modify the biological response to environmental exposures [20]. To our knowledge, this is the first study to examine the relationship between baseline PFAS concentrations and postoperative weight outcomes in adolescents undergoing bariatric surgery, a population at heightened risk for both environmental vulnerability and long‐term obesity‐related complications.

Adolescents undergoing bariatric surgery represent a unique and high‐risk population for studying the interplay between environmental exposures and weight dynamics. Unlike adults, adolescents are still undergoing significant developmental and metabolic changes, making them potentially more vulnerable to the effects of environmental chemicals such as PFAS [21, 22]. Additionally, the rapid and substantial weight loss induced by surgery provides an opportunity to assess how PFAS exposure may influence postoperative weight trajectories, including the extent and timing of weight regain [23]. Understanding these relationships is critical for identifying modifiable risk factors that could enhance the long‐term success of bariatric surgery and improve health outcomes in this vulnerable population.

This study examines the association between baseline plasma PFAS concentrations and longitudinal changes in BMI, percent weight loss, and waist circumference over 5 years following bariatric surgery in adolescents enrolled in the Teen‐Longitudinal Assessment of Bariatric Surgery (Teen‐LABS) study [24]. We hypothesize that higher baseline PFAS concentrations will be associated with greater BMI regain, reduced percent weight loss, and increased waist circumference following bariatric surgery in adolescents. By leveraging a well‐characterized cohort, detailed longitudinal data, and advanced statistical modeling, this research aims to provide novel insights into the role of PFAS in postoperative weight dynamics. The findings from this study have the potential to inform strategies to mitigate weight regain and improve the long‐term management of severe obesity in adolescents, with implications for clinical care and public health policy.

## 2 Methods

### 2.1 Study Design

The Teen‐Longitudinal Assessment of Bariatric Surgery (Teen‐LABS) study was conducted between 2007 and 2012, enrolling adolescents (≤ 19 years of age) who were at Tanner Stage 4 or higher and undergoing either Roux‐en‐Y gastric bypass or sleeve gastrectomy [24]. The Teen‐LABS study focused on evaluating the safety and effectiveness of bariatric surgery in adolescents with severe obesity. Participants were eligible for bariatric surgery if their body mass index (BMI) was ≥ 35 kg/m^2^ and they had at least one obesity‐related comorbidity [24]. The study was carried out at five US clinical centers: Cincinnati Children's Hospital Medical Center (Cincinnati, OH), Nationwide Children's Hospital (Columbus, OH), University of Pittsburgh Medical Center (Pittsburgh, PA), Texas Children's Hospital (Houston, TX), and Children's Hospital of Alabama (Birmingham, AL). Participants were followed longitudinally for 5 years, with study visits conducted preoperatively and at 6 months, 1 year, 3 years, and 5 years post surgery. Detailed descriptions of the study design and methodology have been published previously [23, 24].

The Teen‐LABS study received approval from the Institutional Review Boards (IRB) at all participating sites. Written informed consent and assent were obtained from all participants and their guardians. The present ancillary study was approved by the University of Southern California IRB (HS‐19‐00919).

### 2.2 Data Collection

Baseline data were collected during in‐person visits conducted ≤ 30 days before surgery. Follow‐up visits were carried out either in clinical settings or participants' homes, following standardized protocols administered by trained study personnel [24]. Fasting blood samples were collected at each study visit. All collected data were centralized and managed at a data coordinating center.

### 2.3 Measurement of PFAS

The samples were transported on dry ice with temperature logging by World Courier (AmerisourceBergen Corp.) and stored at −80°C until analysis. The samples were analyzed by online solid‐phase extraction followed by liquid chromatography–tandem mass spectrometry (LC–MS/MS), as previously described [25, 26]. The limit of detection (LOD) was 0.03 ng/mL for all reported compounds. All PFAS measurements exceeded the LOD except for PFHpA (9.1% below LOD) and PFUnDA (8.1% below LOD). For these, values below the LOD were imputed as 1\2 LOD. The batch imprecision for the quality control samples was less than 6% for all measured compounds. Plasma concentrations of PFAS measured in Teen‐LABS participants have been previously published [27].

### 2.4 Outcomes

At each study visit, BMI (kg/m^2^) was determined using height and weight measurements taken in triplicate with a calibrated stadiometer and electronic scale. Waist circumference was measured three times at the midpoint between the lower rib margin and the iliac crest [23, 24]. Percent weight loss since surgery was calculated at each visit using the formula:

$$\text{Percent weight loss}=\frac{\left(\text{Weight lost}-\text{Baseline weight}\right)}{\text{Baseline weight}}\times 100\%$$

### 2.5 Covariates

At the baseline visit, demographic and socioeconomic information was collected, including sex (female or male), study site, race and ethnicity, and parental income. Parental income was categorized into four groups: < $25,000, $25,000–$74,000, $75,000+, and unknown. Study site was dichotomized as Cincinnati (CIN) or Other, while race and ethnicity were categorized as White or Other. Participants' age (in years) was recorded at each visit. The homeostatic model assessment for insulin resistance (HOMA‐IR) was calculated from fasting glucose and insulin levels using the following formula:

HOMA‐IR = $\frac{\text{Glucose}\;\left(\mathrm{mg}/\mathrm{dL}\right)\times \text{Insulin}\;\left(\mathrm{mg}/\mathrm{dL}\right)}{405}$.

### 2.6 Statistical Analysis

Descriptive statistics were calculated for all outcomes, PFAS concentrations at baseline, and covariates at each visit. Continuous variables were summarized as means with standard deviations (SD), and categorical variables were presented as counts and percentages. The sums of perfluoroalkyl carboxylic acids (PFOA, PFDA, PFHpA, and PFUnDA; PFCAs) and perfluoroalkyl sulfonic acids (PFOS, PFHxS, and PFHpS; PFSAs) were computed for analysis.

Linear mixed‐effects models were employed to assess the impact of baseline PFAS exposure on longitudinal changes in BMI, percent weight loss, and waist circumference. PFAS were analyzed in two ways: as continuous variables and as tertiles. For continuous models, each PFAS and the summed PFAS were log_2_‐transformed to meet model assumptions. For tertile‐based models, PFAS concentrations were divided into tertiles based on their original ng/mL distribution to evaluate potential concentration‐dependent relationships.

The linear mixed models included a random intercept for each participant and interactions between PFAS and time of study visit (in years). A linear B‐spline with a knot at 1 year was incorporated to account for the observed trajectory of weight loss, which peaked at 1 year, followed by weight regain in subsequent visits. We included a knot at 1 year post surgery in the B‐spline model to account for nonlinear changes in weight trajectory. This decision was informed by prior Teen‐LABS publications indicating that the greatest metabolic improvements occur within the first year following bariatric surgery [28]. We also explored alternative knot placements using data‐driven approaches, and the 1‐year knot consistently provided the best model fit while maintaining clinical relevance. The models provided estimates for the effect of time (visit) on weight loss and the combined effect of time and PFAS exposure. Associations between PFAS and outcomes were reported for two time periods: the first year after surgery and the 1‐ to 5‐year follow‐up period. Predicted weight loss trajectories for each PFAS tertile were calculated for both periods, and the mean outcome values at each tertile and visit were compared for significant differences (*p* < 0.05). All models were adjusted for participants' age at each visit, sex, race, study site, and parental income.

To evaluate the effects of PFAS mixtures on outcomes at 5 years post surgery, quantile g‐computation (QGC) was applied [29]. QGC allows for associations between the components of the exposure mixture and outcome to be either positive and negative [29]. Because different PFAS congeners may have varied biological activity, we do not expect that the effect of each will be in the same direction for all outcomes. Unlike individual PFAS models, PFAS levels were not log_2_‐transformed for QGC analysis. Three PFAS mixtures were analyzed using QGC: all seven PFAS combined, PFCAs only, and PFSAs only. QGC models provided estimates for the effects of increasing each PFAS chemical by one quartile. PFAS tertile and quartile ranges are described in Table S2.

We also performed a sensitivity analysis to assess the potential for confounding by insulin sensitivity (as HOMA‐IR) on the relationship between PFAS exposure and weight loss. HOMA‐IR was included as a time‐varying covariate in the linear mixed models, and the QGC mixtures model additionally adjusted for HOMA‐IR at baseline.

All statistical analyses were performed using R version 4.4.1. Linear mixed models were constructed with the nlme (lme function) and splines2 (bSpline function) packages. Contrasts for tertile models were calculated using the contrast package (contrast function), while associations in the continuous log_2_‐transformed PFAS models were estimated using the emtrends function from the emmeans package. QGC analyses were conducted using the qgcomp and epiomics packages. Data visualization and predicted trajectories were generated with the ggplot2 and ggeffects (ggpredict function) packages.

## 3 Results

### 3.1 Cohort Characteristics

The study cohort characteristics can be found in Table 1 and consisted of 186 participants at baseline, with a mean age of 17.1 years (SD: 1.5). The majority of participants were female (76.3%), White (72.0%), and primarily from study site CIN (55.4%). Parental income was most commonly reported within the $25,000 to $75,000 range. At baseline, the mean BMI was 52.5 kg/m^2^ (SD: 9.2), and the mean waist circumference was 139.0 cm (SD: 17.1). Participants experienced a mean weight loss of 30.5% of their baseline body weight (SD: 9.5%) at 1 year post surgery, which decreased to 23.5% (SD: 15.4%) by 5 years.

**TABLE 1:** Demographics and outcome distributions by year of visit.

	Mean (SD) or *n* (%)	Median (IQR)	Missing
BMI (kg/m^2^)			
Baseline	52.5 (9.2)	50.5 (34.0 to 87.7)	
0.5 year	39.6 (8.7)	38.0 (24.5 to 67.6)	44
1 year	36.5 (9.0)	34.5 (22.4 to 64.8)	50
3 years	38.9 (11.6)	35.9 (22.2 to 79.8)	68
5 years	40.5 (12.0)	38.0 (22.3 to 91.5)	25
Waist circumference (cm)			
Baseline	139.0 (17.1)	138.0 (98.8 to 184.0)	1
0.5 year	114.0 (18.5)	112.0 (77.0 to 165.0)	48
1 year	107.0 (19.1)	106.0 (75.3 to 168.0)	53
3 years	110.0 (21.3)	107.0 (74.5 to 168.0)	72
5 years	111.0 (21.8)	109.0 (76.0 to 172.0)	56
% Weight loss			
0.5 year	−24.8 (7.2)	−26.1 (−42.7 to −3.0)	44
1 year	−30.4 (9.5)	−30.5 (−52.8 to −5.16)	49
3 years	−26.2 (14.5)	−27.2 (−55.7 to 10.7)	67
5 years	−23.5 (15.4)	−23.7 (−60.3 to 10.8)	21
HOMA‐IR			
Baseline	7.26 (6.11)	5.75 (0.18 to 43.20)	2
0.5 year	3.76 (5.26)	2.51 (0.30 to 35.90)	40
1 year	2.68 (2.79)	1.98 (0.55 to 21.60)	47
3 years	2.82 (2.71)	1.85 (0.38 to 19.10)	63
5 years	2.96 (3.20)	1.91 (0.44 to 21.10)	37
Age at baseline (years)	17.1 (1.54)	17.2 (13.2 to 20.0)	
Parents' annual income, *n* (%)			
< $25,000	65 (34.9%)		
$25,000 to < 75,0000	70 (37.6%)		
≥ $75,000	42 (22.6%)		
Unknown	9 (4.8%)		
Sex, *n* (%)			
Male	44 (23.7%)		
Female	142 (76.3%)		
Race, *n* (%)			
White	134 (72.0%)		
Other	52 (28.0%)		
Site, *n* (%)			
CIN	103 (55.4%)		
All other sites	83 (44.6%)		

### 3.2 BMI Changes

**FIGURE 1:**
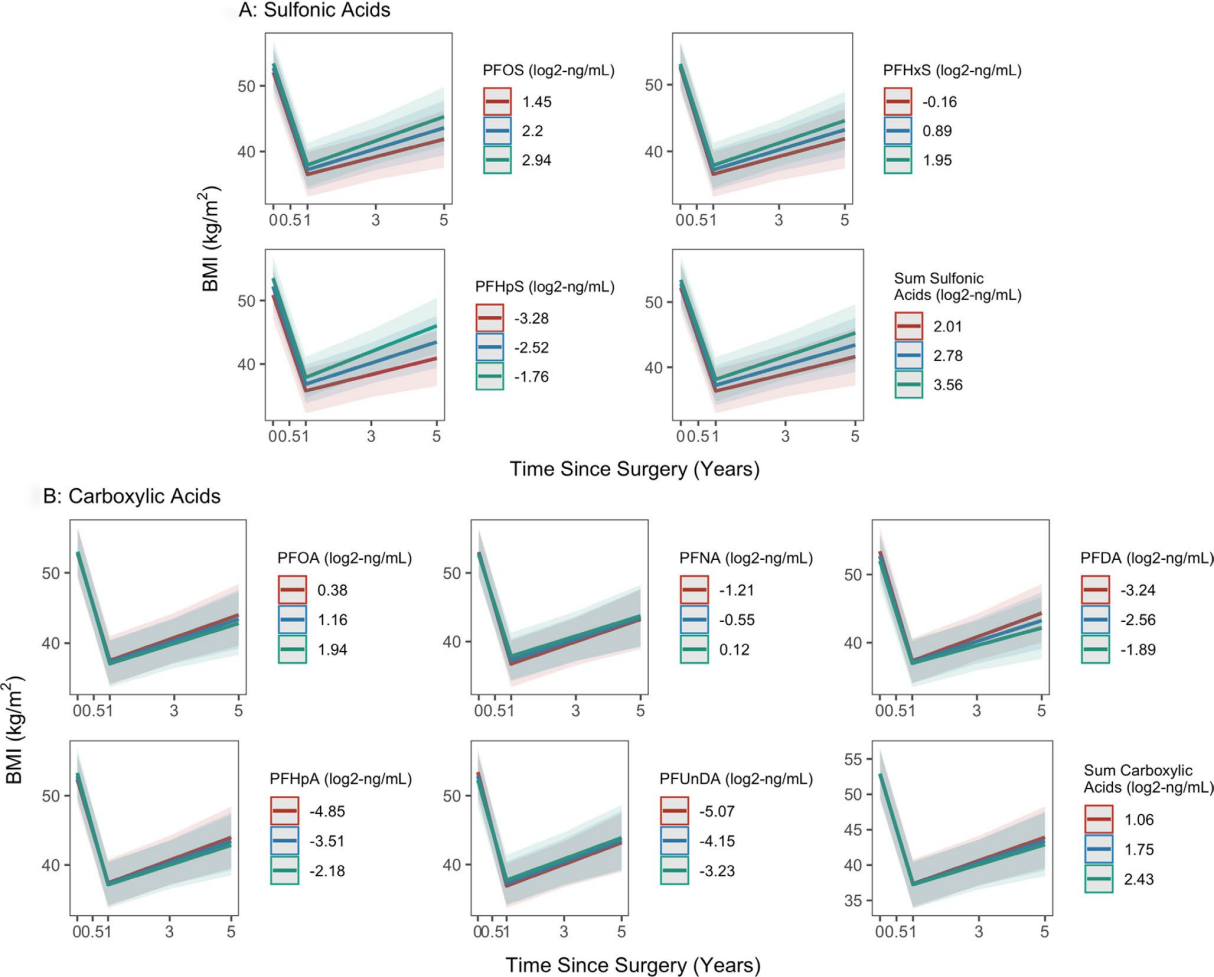
Mean predicted BMI at baseline and after surgery. Trajectories are stratified by representative values of log_2_‐transformed PFAS for (A) sulfonic acid congeners and (B) carboxylic acid congeners. [Color figure can be viewed at wileyonlinelibrary.com]

Significant positive associations were observed between continuous PFAS levels and the second‐period spline (years 1–5) for PFOS, PFHxS, and PFHpS individually and for the sum of PFSAs, as shown in Figure 1, Table 2, and Figure S1. These findings indicate that individuals with higher PFAS concentrations demonstrated less pronounced weight loss and a greater rate of BMI regain during this period. For example, at a PFOS concentration of 1.45 log_2_ ng/mL (2.73 ng/mL), the estimated rate of BMI regain was 1.34 kg/m^2^ per year (95% CI: 0.44, 2.24). This rate increased to 1.84 kg/m^2^ per year (95% CI: 0.94, 2.73) at a concentration of 2.94 log_2_ ng/mL (7.67 ng/mL), and the interaction was statistically significant (*β*_interaction_ = 1.38, *p* = 0.0497). Similarly, for PFHxS, at a concentration of −0.16 log_2_ ng/mL (0.90 ng/mL), the rate of BMI regain was 1.33 kg/m^2^ per year (95% CI: 0.43, 2.23), which increased to 1.50 kg/m^2^ per year (95% CI: 0.65, 2.35) at a level of 1.95 log_2_ ng/mL (3.86 ng/mL) (*β*_interaction_ = 1.04, *p* = 0.036). For PFHpS, the rate of BMI regain was 1.29 kg/m^2^ per year (95% CI: 0.39, 2.18) at −3.28 log_2_ ng/mL (0.10 ng/mL), which increased to 2.04 kg/m^2^ per year (95% CI: 1.15, 2.93) at −1.76 log_2_ ng/mL (0.30 ng/mL) (*β*_interaction_ = 2.39, *p* = 0.017). Additionally, for the sum of PFSAs, the rate of BMI regain was 1.31 kg/m^2^ per year (95% CI: 0.42, 2.21) at 2.01 log_2_ ng/mL (4.03 ng/mL), increasing to 1.77 kg/m^2^ per year (95% CI: 0.89, 2.66) at 3.56 log_2_ ng/mL (11.79 ng/mL) (*β*_interaction_ = 2.30, *p* = 0.022).

**TABLE 2:** Mean BMI (kg/m^2^) and 95% CI predictions per time since bariatric surgery, stratified by level of PFAS exposure.

	Time since surgery					Annual change			
	Baseline	0.5 year	1 year	3 years	5 years	Year 1	*p* ^a^	Years 1–5	*p* ^a^
PFOS									
T1	52.90 (49.06, 56.12)	45.01 (41.76, 48.27)	37.44 (34.13, 40.75)	39.90 (36.63, 43.16)	42.36 (38.18, 46.54)	−15.15 (−17.25, −13.05)		1.23 (0.29, 2.18)	
T2	55.39 (51.76, 59.02)	47.19 (43.79, 50.58)	38.98 (35.51, 42.45)	42.43 (38.88, 45.98)	45.87 (41.37, 50.37)	−16.41 (−18.52, −14.30)	0.36	1.72 (0.78, 2.67)	0.17
T3	52.91 (49.20, 56.62)	45.44 (41.92, 48.96)	37.97 (34.30, 41.63)	41.55 (37.38, 45.38)	45.14 (40.35, 49.93)	−14.95 (−17.13, −12.77)	0.89	1.79 (0.85, 2.74)	0.12
1.45 log_2_ ng/mL						−15.54 (−17.35, −13.72)		1.34 (0.44, 2.24)	
2.20 log_2_ ng/mL						−15.50 (−16.93, −14.06)	0.95	1.59 (0.74, 2.44)	0.05
2.94 log_2_ ng/mL						−15.46 (−17.29, −13.62)		1.84 (0.94, 2.73)	
PFHxS									
T1	53.13 (49.59, 56.67)	45.38 (42.09, 48.67)	37.63 (34.25, 41.01)	40.12 (36.69, 43.54)	42.61 (38.22, 47.00)	−15.50 (−17.62, −13.38)		1.25 (0.30, 2.19)	
T2	53.94 (50.29, 57.58)	45.89 (42.49, 49.29)	37.84 (34.39, 41.30)	41.34 (37.82, 44.86)	44.84 (40.40, 49.27)	−16.09 (−18.17, −14.02)	0.67	1.75 (0.82, 2.68)	0.16
T3	54.00 (50.26, 57.73)	46.47 (42.96, 49.98)	38.94 (35.31, 42.57)	42.02 (38.40, 45.63)	45.09 (40.62, 49.57)	−15.02 (−17.27, −12.84)	0.76	1.54 (0.60, 2.48)	0.43
−0.16 log_2_ ng/mL						−15.99 (−17.81, −14.17)		1.33 (0.43, 2.23)	
0.89 log_2_ ng/mL						−15.60 (−17.04, −14.17)	0.49	1.50 (0.65, 2.35)	0.04
1.95 log_2_ ng/mL						−15.21 (−17.02, −13.39)		1.68 (0.79, 2.57)	
PFHpS									
T1	53.19 (49.58, 56.80)	45.45 (42.11, 48.80)	37.72 (34.31, 41.13)	40.36 (37.01, 43.70)	42.99 (38.77, 47.21)	−15.47 (−17.60, −13.33)		1.32 (0.38, 2.26)	
T2	52.87 (49.30, 56.43)	45.37 (42.05, 48.70)	37.88 (34.48, 41.29)	40.24 (36.74, 43.75)	42.61 (38.15, 47.06)	−14.98 (−17.05, −12.91)	0.73	1.18 (0.25, 2.11)	0.70
T3	54.90 (51.23, 58.57)	46.91 (43.44, 50.38)	38.92 (35.32, 42.52)	43.61 (39.88, 47.33)	48.29 (43.63, 52.96)	−15.98 (−18.12, −13.85)	0.71	2.34 (1.41, 3.28)	0.005
−3.28 log_2_ ng/mL						−15.13 (−16.95, −13.31)		1.29 (0.39, 2.18)	
−2.52 log_2_ ng/mL						−15.39 (−16.82, −13.96)	0.64	1.66 (0.82, 2.51)	0.02
−1.76 log_2_ ng/mL						−15.66 (−17.49, −13.83)		2.04 (1.15, 2.93)	
Sum sulfonic acids									
T1	52.68 (49.24, 56.11)	45.01 (41.84, 48.18)	37.35 (34.10, 40.59)	40.01 (36.75, 43.27)	42.67 (38.45, 46.90)	−15.33 (−17.47, −13.19)		1.33 (0.39, 2.28)	
T2	55.01 (51.32, 58.70)	46.68 (43.20, 50.15)	38.34 (34.79, 41.90)	41.58 (37.91, 45.24)	44.81 (40.20, 49.42)	−16.67 (−18.73, −14.60)	0.33	1.62 (0.68, 2.55)	0.43
T3	54.15 (50.28, 58.01)	46.90 (43.25, 50.54)	39.64 (35.90, 43.39)	42.93 (39.20, 46.65)	46.21 (41.66, 50.77)	−17.47 (−16.70, −12.30)	0.56	1.64 (0.71, 2.58)	0.39
2.01 log_2_ ng/mL						−15.83 (−17.65, −14.01)		1.31 (0.42, 2.21)	
2.78 log_2_ ng/mL						−15.55 (−16.98, −14.11)	0.62	1.54 (0.70, 2.39)	0.02
3.56 log_2_ ng/mL						−15.25 (−17.08, −13.43)		1.77 (0.89, 2.66)	
PFOA									
T1	52.94 (49.51, 56.38)	45.34 (42.18, 48.49)	37.73 (34.50, 40.96)	40.98 (37.74, 44.22)	44.23 (40.02, 48.45)	−15.21 (−17.35, −13.07)		1.63 (0.68, 2.58)	
T2	54.25 (50.45, 58.06)	46.09 (42.53, 49.66)	37.93 (34.29, 41.57)	41.31 (37.65, 44.96)	44.68 (40.15, 49.22)	−16.32 (−18.47, −14.17)	0.43	1.69 (0.75, 2.63)	0.39
T3	53.73 (49.87, 57.58)	46.14 (42.46, 49.83)	38.56 (34.75, 42.38)	41.19 (37.19, 45.19)	43.81 (38.85, 48.78)	−15.16 (−17.30, −13.03)	0.97	1.31 (0.36, 2.27)	0.30
0.38 log_2_ ng/mL						−15.24 (−17.06, −13.41)		1.62 (0.72, 2.53)	
1.16 log_2_ ng/mL						−15.58 (−17.02, −14.13)	0.55	1.53 (0.67, 2.38)	0.17
1.94 log_2_ ng/mL						−15.92 (−17.75, −14.09)		1.43 (0.53, 2.34)	
PFNA									
T1	55.35 (51.79, 58.91)	46.82 (43.53, 50.11)	38.29 (34.94, 41.64)	41.37 (38.05, 44.69)	44.45 (40.21, 48.68)	−17.06 (−19.18, −14.95)		1.54 (0.60, 2.48)	
T2	51.40 (47.58, 55.22)	43.85 (40.24, 47.46)	36.30 (32.61, 40.00)	39.40 (35.62, 43.18)	42.50 (37.82, 47.19)	−15.10 (−17.20, −13.00)	0.16	1.55 (0.62, 2.49)	0.98
T3	52.82 (49.23, 56.40)	45.50 (42.13, 48.87)	38.19 (34.69, 41.69)	41.01 (37.43, 44.58)	43.82 (39.31, 48.34)	−14.62 (−16.80, −12.45)	0.09	1.41 (0.47, 2.35)	0.72
−1.21 log_2_ ng/mL						−16.30 (−18.13, −14.47)		1.62 (0.72, 2.52)	
−0.55 log_2_ ng/mL						−15.54 (−16.99, −14.10)	0.19	1.55 (0.69, 2.40)	0.38
0.12 log_2_ ng/mL						−14.77 (−16.63, −12.92)		1.47 (0.57, 2.38)	
PFDA									
T1	54.27 (50.83, 57.71)	46.21 (43.01, 49.40)	38.15 (34.85, 41.44)	41.75 (38.43, 45.07)	45.35 (41.08, 49.62)	−16.12 (−18.24, −13.99)		1.80 (0.87, 2.74)	
T2	53.30 (49.55, 57.05)	45.46 (41.95, 48.97)	37.62 (34.05, 41.19)	41.26 (37.67, 44.85)	44.90 (40.41, 49.40)	−15.68 (−17.80, −13.57)	0.76	1.82 (0.87, 2.77)	0.95
T3	52.22 (48.44, 56.00)	44.77 (41.21, 48.33)	37.32 (33.67, 40.98)	39.36 (35.64, 43.08)	41.40 (36.79, 46.01)	−14.90 (−17.04, −12.76)	0.38	1.02 (0.09, 1.95)	0.03
−3.24 log_2_ ng/mL						−16.12 (−17.95, −14.29)		1.75 (0.86, 2.65)	
−2.56 log_2_ ng/mL						−15.58 (−17.02, −14.14)	0.35	1.53 (0.67, 2.38)	0.48
−1.89 log_2_ ng/mL						−15.04 (−16.88, −13.21)		1.30 (0.41, 2.19)	
PFHpA									
T1	53.11 (49.61, 56.61)	45.81 (42.55, 49.07)	38.51 (35.14, 41.88)	41.70 (38.23, 45.18)	44.89 (40.42, 49.36)	−14.60 (−16.78, −12.42)		1.60 (0.65, 2.54)	
T2	53.51 (49.83, 57.18)	45.25 (41.81, 48.69)	36.99 (33.48, 40.51)	40.57 (37.04, 44.11)	44.15 (39.71, 48.59)	−16.51 (−18.61, −14.42)	0.17	1.79 (0.85, 2.73)	0.60
T3	53.78 (50.00, 57.56)	46.01 (42.48, 49.53)	38.23 (34.64, 41.82)	40.73 (37.18, 44.28)	43.22 (38.81, 47.64)	−15.55 (−17.67, −13.42)	0.50	1.25 (0.30, 2.20)	0.34
−4.85 log_2_ ng/mL						−15.00 (−16.84, −13.15)		1.65 (0.75, 2.54)	
−3.51 log_2_ ng/mL						−15.57 (−17.00, −14.13)	0.32	1.54 (0.68, 2.39)	0.05
−2.18 log_2_ ng/mL						−16.14 (−17.96, −14.33)		1.43 (0.52, 2.34)	
PFUnDA									
T1	54.55 (50.99, 58.12)	46.24 (42.92, 49.56)	37.92 (34.54, 41.30)	40.94 (37.58, 44.29)	43.95 (39.69, 48.21)	−16.63 (−18.68, −14.58)		1.51 (0.57, 2.45)	
T2	52.85 (49.19, 56.52)	45.25 (41.82, 48.68)	37.64 (34.11, 41.18)	41.06 (37.46, 44.67)	44.48 (39.93, 49.04)	−15.21 (−17.40, −13.01)	0.31	1.71 (0.76, 2.66)	0.58
T3	52.99 (49.26, 56.72)	45.62 (42.12, 49.12)	38.24 (34.64, 41.85)	41.07 (37.39, 44.75)	43.90 (39.30, 48.50)	−14.75 (−16.92, −12.58)	0.17	1.41 (0.48, 2.35)	0.79
−5.07 log_2_ ng/mL						−16.56 (−18.38, −14.75)		1.57 (0.66, 2.48)	
−4.15 log_2_ ng/mL						−15.55 (−16.99, −14.11)	0.08	1.56 (0.70, 2.41)	0.07
−3.23 log_2_ ng/mL						−14.53 (−16.37, −12.69)		1.54 (0.65, 2.44)	
Sum carboxylic acids									
T1	53.56 (50.13, 57.00)	45.87 (42.71, 49.02)	38.18 (34.95, 41.40)	41.32 (38.11, 44.53)	44.46 (40.29, 48.64)	−15.39 (−17.54, −13.24)		1.57 (0.62, 2.53)	
T2	53.48 (49.66, 57.29)	45.30 (41.70, 48.89)	37.12 (33.43, 40.80)	40.54 (36.78, 44.29)	43.96 (39.30, 48.62)	−16.36 (−18.52, −14.21)	0.49	1.71 (0.77, 2.65)	0.70
T3	53.30 (49.43, 57.17)	45.78 (42.09, 49.48)	38.26 (34.45, 41.08)	40.81 (36.84, 44.78)	43.36 (38.44, 48.28)	−15.04 (−17.16, −12.92)	0.80	1.28 (0.32, 2.23)	0.41
1.06 log_2_ ng/mL						−15.49 (−17.33, −13.66)		1.65 (0.74, 2.55)	
1.75 log_2_ ng/mL						−15.58 (−17.02, −14.13)	0.88	1.53 (0.67, 2.39)	0.30
2.43 log_2_ ng/mL						−15.66 (−17.50, −13.83)		1.41 (0.51, 2.32)	

*Note*: Adjusted for race, sex, study site, age at baseline, and parents' income. PFAS levels reported for a participant with the following characteristics: age 19 at baseline, White, female, parent's income < $25,000, study site CIN.

^a^ For tertiles: significance test for difference from tertile 1. For log2‐PFAS: significance of the PFAS × Spline interaction term.

In contrast, a significant negative interaction was observed between PFHpA and the second‐period spline, suggesting that higher PFHpA concentrations were associated with lower rates of BMI regain (*β*_interaction_ = −1.98, *p* = 0.048). In tertile analyses, most associations were nonsignificant. However, for PFDA, BMI regain was significantly lower in the third tertile (1.02 kg/m^2^ per year; 95% CI: 0.09, 1.95) compared to the first tertile (1.80 kg/m^2^ per year; 95% CI: 0.87, 2.74), with a *p* value of 0.03.

### 3.3 Percentage of Weight Lost

**FIGURE 2:**
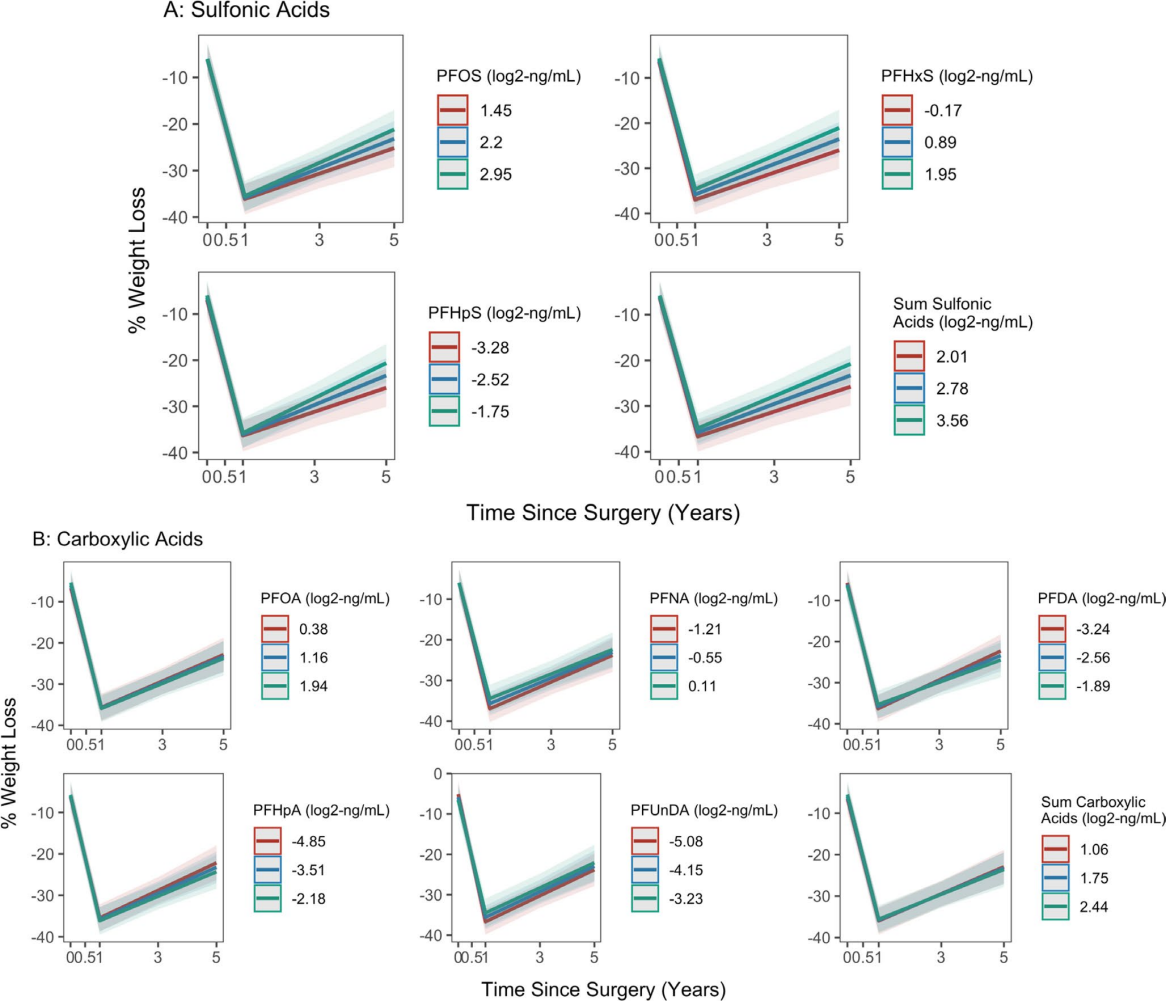
Mean predicted percent weight loss at baseline and after surgery. Trajectories are stratified by representative values of log_2_‐transformed PFAS for (A) sulfonic acid congeners and (B) carboxylic acid congeners. [Color figure can be viewed at wileyonlinelibrary.com]

Similar patterns were observed for the percentage of weight lost, with significant positive PFAS and second‐period spline interactions for PFOS, PFHxS, PFHpS, and the sum of PFSAs, as presented in Figure 2, Table 3, and Figure S1. For PFOS, at a concentration of 1.45 log_2_ ng/mL (2.73 ng/mL), the rate of weight regain was 2.73 percentage points of baseline body weight per year (95% CI: 1.72, 3.75). This increased to 3.57 percentage points per year (95% CI: 2.57, 6.98) at a concentration of 2.94 log_2_ ng/mL (7.67 ng/mL) (*β*_interaction_ = 2.54, *p* = 0.040). For PFHpS, at a concentration of −3.28 log_2_ ng/mL (0.10 ng/mL), the rate of weight regain was 2.59 percentage points per year (95% CI: 1.57, 3.61), increasing to 3.78 percentage points per year (95% CI: 2.77, 4.79) at −1.76 log_2_ ng/mL (0.30 ng/mL) (*β*_interaction_ = 2.86, *p* = 0.018).

**TABLE 3:** Mean weight lost (% of baseline body weight) and 95% CI predictions per time since bariatric surgery, stratified by level of PFAS exposure.

	Time since surgery				Annual change			
	0.5 year	1 year	3 years	5 years	Year 1	*p* ^a^	Years 1–5	*p* ^a^
PFOS								
T1	−18.34 (−21.31, −15.37)	−33.02 (−36.42, −29.63)	−27.54 (−30.54, −24.54)	−22.05 (−26.14, −17.97)	−29.37 (−32.83, −25.91)		2.74 (1.62, 3.87)	
T2	−18.35 (−21.45, −15.25)	−33.75 (−37.28, −30.22)	‐ 27.19 (−30.45, −23.92)	−20.62 (−25.00, −16.24)	−30.80 (−34.30, −27.30)	0.56	3.28 (2.15, 4.42)	0.39
T3	−17.90 (−21.13, −14.68)	−32.33 (−36.09, −28.58)	‐ 25.30 (−28.86, −21.74)	−18.27 (−22.92, −13.61)	−28.86 (−32.49, −25.23)	0.84	3.52 (2.37, 4.66)	0.22
1.45 log_2_ ng/mL					−29.94 (−32.86, −27.01)		2.73 (1.72, 3.75)	
2.2 log_2_ ng/mL					−29.71 (−31.86, −27.56)	0.83	3.16 (2.28, 4.03)	**0.04**
2.94 log_2_ ng/mL					−29.49 (−32.43, −26.55)		3.57 (2.57, 6.98)	
PFHxS								
T1	−18.44 (−21.43, −15.45)	−33.35 (−36.79, −29.92)	−27.97 (−31.08, −24.87)	−22.60 (−26.82, −18.37)	−29.82 (−33.32, −26.33)		2.69 (1.55, 3.83)	
T2	−18.71 (−20.34, −15.66)	−34.05 (−37.49, −30.60)	−27.13 (−30.33, −23.92)	−20.21 (−24.49, −15.92)	−30.66 (−34.10, −27.23)	0.73	3.46 (2.36, 4.56)	0.22
T3	−17.12 (−20.24, −13.90)	−31.44 (−35.19, −27.68)	−25.23 (−28.58, −21.88)	−19.03 (−23.38, −14.67)	−28.63 (−32.29, −24.98)	0.64	3.10 (1.96, 4.25)	0.52
−0.16 log_2_ ng/mL					−30.54 (−33.45, −27.63)		2.74 (1.73, 3.76)	
0.89 log_2_ ng/mL					−29.78 (−31.93, −27.63)	0.44	3.07 (2.20, 3.94)	**0.02**
1.95 log_2_ ng/mL					−29.01 (−31.92, −26.10)		3.39 (2.40, 4.39)	
PFHpS								
T1	−18.67 (−21.72, −15.63)	−33.39 (−36.88, −29.90)	−28.03 (−31.10, −24.97)	−22.68 (−26.80, −18.55)	−29.43 (−32.95, −25.92)		2.68 (1.54, 3.82)	
T2	−17.94 (−20.96, −14.92)	−32.76 (−36.20, −29.33)	−27.59 (−30.79, −24.39)	−22.41 (−26.72, −18.10)	−29.65 (−33.09, −26.21)	0.93	2.59 (1.48, 3.70)	0.89
T3	−17.90 (−21.06, −14.74)	−32.83 (−36.49, −29.16)	−24.21 (−27.63, −20.78)	−15.59 (−20.07, −11.10)	−29.84 (−33.37, −26.31)	0.87	4.31 (3.19, 5.43)	**0.01**
−3.28 log_2_ ng/mL					−29.41 (−32.33, −26.48)		2.59 (1.57, 3.61)	
−2.52 log_2_ ng/mL					−29.62 (−31.77, −27.47)	0.83	3.19 (2.31, 4.06)	**0.02**
−1.76 log_2_ ng/mL					−29.84 (−32.78, −26.90)		3.78 (2.77, 4.79)	
Sum sulfonic acids								
T1	−18.37 (−21.24, −15.50)	−33.11 (−36.44, −29.78)	−27.49 (−30.45, −24.53)	−21.88 (−25.97, −17.78)	−29.47 (−32.99, −25.95)		2.81 (1.67, 3.95)	
T2	−18.55 (−21.68, −15.42)	−34.58 (−38.12, −31.04)	−28.30 (−31.64, −24.95)	−22.01 (−26.46, −17.57)	−32.06 (−35.47, −28.65)	0.29	3.14 (2.03, 4.25)	0.60
T3	−16.89 (−20.21, −13.58)	−30.64 (−34.46, −26.82)	−24.23 (−27.66, −20.80)	−17.82 (−22.20, −13.43)	−27.49 (−31.12, −23.85)	0.43	3.21 (2.07, 4.34)	0.53
2.01 log_2_ ng/mL					−30.35 (−33.28, −27.43)		2.72 (1.71, 3.74)	
2.78 log_2_ ng/mL					−29.73 (−31.87, −27.58)	0.54	3.12 (2.25, 4.00)	**0.02**
3.56 log_2_ ng/mL					−29.09 (−32.03, −26.15)		3.53 (2.53, 4.53)	
PFOA								
T1	−18.96 (−21.83, −16.09)	−33.70 (−37.03, −30.37)	−26.85 (−29.82, −23.88)	−20.01 (−24.13, −15.88)	−29.48 (−33.01, −25.95)		3.42 (2.28, 4.57)	
T2	−18.50 (−21.72, −15.27)	−33.61 (−37.26, −29.95)	−27.10 (−30.44, −23.76)	−20.59 (−24.95, −16.22)	−30.23 (−33.77, −26.68)	0.76	3.26 (2.14, 4.38)	0.79
T3	−17.01 (−20.35, −13.66)	−31.73 (−35.56, −27.90)	−26.11 (−29.77, −22.45)	−20.50 (−25.25, −15.75)	−29.44 (−32.96, −25.92)	0.99	2.81 (1.66, 3.95)	0.33
0.38 log_2_ ng/mL					−28.96 (−31.90, −26.02)		3.20 (2.18, 4.22)	
1.16 log_2_ ng/mL					−29.76 (−31.92, −27.60)	0.43	3.12 (2.24, 4.00)	0.22
1.94 log_2_ ng/mL					−30.56 (−33.49, −27.64)		3.04 (2.03, 4.05)	
PFNA								
T1	−18.87 (−21.87, −15.88)	−34.40 (−37.83, −30.97)	−28.15 (−31.21, −25.10)	−21.91 (−26.07, −17.74)	−31.06 (−34.56, −27.55)		3.12 (1.98, 4.27)	
T2	−18.13 (−21.39, −14.87)	−33.08 (−36.76, −29.40)	−26.47 (−29.93, −23.02)	−19.86 (−24.37, −15.36)	−29.91 (−33.38, −26.43)	0.63	3.31 (2.19, 4.42)	0.78
T3	−17.73 (−20.84, −14.62)	−31.76 (−35.40, −28.12)	−25.69 (−29.01, −22.38)	−19.62 (−24.01, −15.24)	−28.06 (−31.68, −24.44)	0.23	3.04 (1.89, 4.18)	0.89
−1.21 log_2_ ng/mL					−30.93 (−33.87, −27.99)		3.29 (2.27, 4.31)	
−0.55 log_2_ ng/mL					−29.70 (−31.86, −27.54)	0.23	3.15 (2.27, 4.03)	0.46
0.12 log_2_ ng/mL					−28.46 (−31.45, −25.47)		3.01 (1.99, 4.03)	
PFDA								
T1	−18.13 (−21.03, −15.24)	−33.14 (−36.53, −29.75)	−26.19 (−29.23, −23.15)	−19.23 (−23.36, −15.11)	−30.01 (−33.54, −26.48)		3.48 (2.36, 4.60)	
T2	−18.52 (−21.70, −15.35)	−33.79 (−37.36, −30.22)	−26.58 (−29.87, −23.29)	−19.36 (−23.74, −14.98)	−30.54 (−34.02, −27.05)	0.83	3.61 (2.48, 4.74)	0.84
T3	−18.99 (−22.22, −15.75)	−33.41 (−37.10, −29.72)	−28.96 (−32.34, −25.58)	−24.51 (−28.92, −20.10)	−28.84 (−32.38, −25.31)	0.64	2.22 (1.10, 3.35)	**0.05**
−3.24 log_2_ ng/mL					−30.65 (−33.61, −27.70)		3.51 (2.50, 4.53)	
−2.56 log_2_ ng/mL					−29.79 (−31.94, −27.63)	0.40	3.11 (2.23, 3.99)	0.42
−1.89 log_2_ ng/mL					−28.93 (−31.88, −25.99)		2.71 (1.70, 3.72)	
PFHpA								
T1	−18.03 (−20.98, −15.09)	−32.28 (−35.72, −28.83)	−25.66 (−28.84, −22.48)	−19.05 (−23.36, −14.74)	−28.49 (−32.08, −24.90)		3.31 (2.18, 4.44)	
T2	−19.40 (−22.51, −16.29)	−35.01 (−38.54, −31.47)	−28.09 (−31.30, −24.87)	−21.17 (−25.46, −16.87)	−31.21 (−34.69, −27.74)	0.27	3.46 (2.33, 4.59)	0.81
T3	−18.01 (−21.21, −14.81)	−32.75 (−36.38, −29.12)	−27.35 (−30.58, −24.11)	−21.95 (−26.19, −17.71)	−29.47 (−32.99, −25.95)	0.69	2.70 (1.57, 3.83)	0.34
−4.85 log_2_ ng/mL					−29.16 (−32.12, −26.21)		3.32 (2.31, 4.33)	
−3.51 log_2_ ng/mL					−29.77 (−31.93, −27.62)	0.55	3.14 (2.26, 4.02)	0.14
−2.18 log_2_ ng/mL					−30.38 (−33.30, −27.46)		2.95 (1.93, 3.98)	
PFUnDA								
T1	−18.59 (−21.60, −15.58)	−34.25 (−37.68, −30.83)	−27.93 (−30.99, −24.87)	−21.62 (−25.71, −17.52)	−31.33 (−34.72, −27.94)		3.16 (2.05, 4.27)	
T2	−17.76 (−20.88, −14.64)	−32.53 (−36.14, −28.92)	−26.18 (−29.51, −22.85)	−19.83 (−24.31, −15.35)	−29.53 (−33.18, −25.89)	0.47	3.17 (2.01, 4.34)	0.98
T3	−18.70 (−21.88, −15.52)	−32.82 (−36.47, −29.17)	−26.58 (−29.94, −23.21)	−20.34 (−24.76, −15.91)	−28.23 (−31.80, −24.67)	0.21	3.12 (1.99, 4.25)	0.95
−5.07 log_2_ ng/mL					−31.55 (−34.49, −28.61)		3.22 (2.20, 4.24)	
−4.15 log_2_ ng/mL					−29.75 (−31.90, −27.59)	0.08	3.16 (2.28, 4.04)	0.09
−3.23 log_2_ ng/mL					−27.95 (−30.89, −25.00)		3.11 (2.10, 4.11)	
Sum carboxylic acids								
T1	−18.62 (−21.49, −15.75)	−33.15 (−36.49, −29.81)	−26.52 (−29.46, −23.59)	−19.90 (−23.98, −15.82)	−29.05 (−32.61, −25.50)		3.31 (2.17, 4.46)	
T2	−18.96 (−22.22, −15.71)	−34.56 (−38.26, −30.87)	−27.93 (−31.37, −24.49)	−21.30 (−25.78, −16.83)	−31.20 (−34.74, −27.66)	0.39	3.32 (2.20, 4.43)	1.00
T3	−17.23 (−20.59, −13.88)	−31.76 (−35.58, −27.94)	−26.26 (−29.89, −22.62)	−20.75 (−25.47, −16.04)	−29.06 (−32.56, −25.56)	1.00	2.75 (1.61, 3.89)	0.38
1.06 log_2_ ng/mL					−29.42 (−32.38, −26.47)		3.26 (2.24, 4.28)	
1.75 log_2_ ng/mL					−29.76 (−31.92, −27.59)	0.75	3.13 (2.25, 4.01)	0.35
2.43 log_2_ ng/mL					−30.08 (−33.02, −27.14)		3.00 (1.98, 4.01)	

*Note*: Adjusted for race, sex, study site, age at baseline, and parents' income. PFAS levels reported for a participant with the following characteristics: age 19 at baseline, White, female, parent's income < $25,000, study site CIN.

^a^ For tertiles: significance test for difference from tertile 1. For log2‐PFAS: significance of the PFAS × Spline interaction term.

In tertile analyses, higher PFHpS levels were associated with greater weight regain. Participants in the highest tertile (tertile 3) exhibited a rate of 4.31 percentage points per year (95% CI: 3.19, 5.43), compared to 2.68 percentage points per year (95% CI: 1.54, 3.82) in tertile 1, with a *p* value of 0.01.

### 3.4 Waist Circumference Changes

**FIGURE 3:**
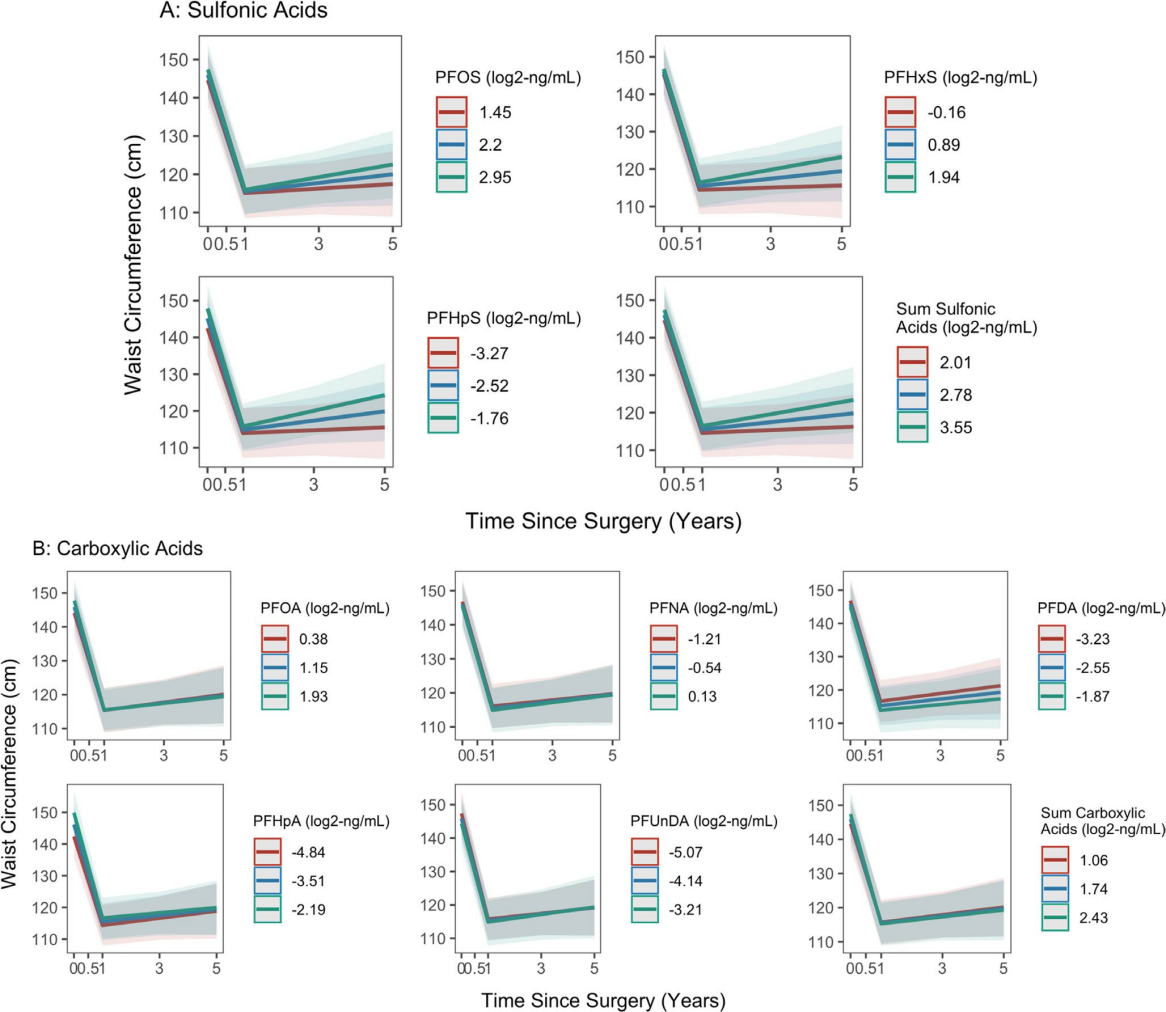
Mean predicted waist circumference at baseline and after surgery. Trajectories are stratified by representative values of log_2_‐transformed PFAS for (A) sulfonic acid congeners and (B) carboxylic acid congeners. [Color figure can be viewed at wileyonlinelibrary.com]

Significant positive interactions were also observed between PFHxS levels and the second‐period spline for waist circumference, suggesting that higher PFHxS levels were associated with greater increases in waist circumference during years 1–5, as shown in Figure 3, Table 4, and Figure S1. At a PFHxS concentration of −0.16 log_2_ ng/mL (0.90 ng/mL), the rate of waist circumference increase was 0.29 cm per year (95% CI: −1.50, 2.08). This rate increased to 1.72 cm per year (95% CI: −0.07, 3.50) at a concentration of 1.95 log_2_ ng/mL (3.86 ng/mL) (*β*_interaction_ = 2.97, *p* = 0.0072).

**TABLE 4:** Mean waist circumference (cm) and 95% CI predictions per time since bariatric surgery, stratified by level of PFAS exposure.

	Time since surgery					Annual change			
	Baseline	0.5 year	1 year	3 years	5 years	Year 1	*p* ^a^	Years 1–5	*p* ^a^
PFOS									
T1	139.02 (132.22, 145.82)	124.91 (118.66, 131.17)	110.80 (104.38, 117.23)	111.41 (105.11, 117.71)	112.01 (103.84, 120.18)	−28.22 (−32.53, −23.91)		0.30 (−1.59, 2.20)	
T2	142.30 (135.31, 149.28)	126.10 (119.60, 132.61)	109.91 (103.19, 116.63)	112.55 (105.67, 119.43)	115.19 (106.28, 124.10)	−32.39 (−36.72, −28.06)	0.15	1.32 (−0.61, 3.25)	0.20
T3	140.34 (133.20, 147.48)	124.87 (118.12, 131.62)	109.41 (102.31, 116.50)	112.73 (105.30, 120.16)	116.06 (106.59, 125.52)	−30.94 (−35.44, −26.43)	0.36	1.66 (−0.26, 3.58)	0.08
1.45 log_2_ ng/mL						−29.54 (−33.25, −25.83)		0.59 (−1.21, 2.38)	
2.2 log_2_ ng/mL						−30.48 (−33.39, −27.57)	0.43	1.12 (−0.56, 2.80)	0.30
2.94 log_2_ ng/mL						−31.41 (−35.16, −27.66)		1.64 (−0.14, 3.43)	
PFHxS									
T1	140.65 (133.91, 147.39)	125.64 (119.40, 131.89)	110.64 (104.17, 117.11)	111.24 (104.70, 117.78)	111.84 (103.32, 120.36)	−30.01 (−34.33, −25.69)		0.30 (−1.60, 2.20)	
T2	139.40 (132.46, 146.33)	123.53 (117.10, 129.96)	107.66 (101.06, 114.27)	110.30 (103.59, 117.02)	112.94 (104.33, 121.56)	−31.73 (−35.97, −27.50)	0.54	1.32 (−0.54, 3.18)	0.19
T3	142.27 (135.16, 149.37)	127.37 (120.72, 134.03)	112.48 (105.51, 119.45)	115.28 (108.34, 122.22)	118.08 (109.32, 126.84)	−29.79 (−34.37, −25.21)	0.94	1.40 (−0.51, 3.31)	0.18
−0.16 log_2_ ng/mL						−30.92 (−34.61, −27.23)		0.29 (−1.50, 2.08)	
0.89 log_2_ ng/mL						−30.63 (−33.52, −27.74)	0.81	1.00 (−0.67, 2.67)	**0.007**
1.95 log_2_ ng/mL						−30.34 (−34.08, −26.60)		1.72 (−0.07, 3.50)	
PFHpS									
T1	139.34 (132.42, 146.26)	125.01 (118.61, 131.40)	110.67 (104.08, 117.27)	111.25 (104.80, 117.69)	111.82 (103.54, 120.10)	−28.66 (−33.07, −24.25)		0.29 (−1.62, 2.20)	
T2	139.22 (132.39, 146.05)	123.97 (117.63, 130.32)	108.73 (102.19, 115.27)	109.81 (103.10, 116.52)	110.89 (102.22, 119.56)	−30.49 (−34.71, −26.27)	0.53	0.54 (−1.33, 2.41)	0.75
T3	143.41 (136.39, 150.44)	127.56 (120.94, 134.18)	111.71 (104.79, 118.93)	117.19 (110.01, 124.37)	122.68 (113.52, 131.83)	−31.70 (36.08, −27.33)	0.30	2.74 (0.86, 4.63)	**0.002**
−3.28 log_2_ ng/mL						−28.49 (−32.21, −24.76)		0.37 (−1.41, 2.16)	
−2.52 log_2_ ng/mL						−30.28 (−33.18, −27.39)	0.13	1.25 (−0.42, 2.92)	0.14
−1.76 log_2_ ng/mL						−32.08 (−35.81, −28.35)		2.13 (0.34, 3.92)	
Sum sulfonic acids									
T1	139.11 (132.53, 145.70)	125.11 (119.07, 131.15)	111.10 (104.85, 117.36)	111.71 (105.44, 117.93)	112.31 (104.06, 120.55)	−28.01 (−32.35, −23.67)		0.30 (−1.59, 2.19)	
T2	141.84 (134.79, 148.89)	124.73 (118.12, 131.35)	107.62 (100.79, 114.45)	109.67 (102.64, 116.70)	111.71 (102.74, 120.69)	−34.22 (−38.40, −30.03)	0.03	1.02 (−0.86, 2.91)	0.36
T3	142.19 (134.80, 149.57)	127.69 (120.75, 134.64)	113.20 (105.98, 120.42)	116.30 (109.11, 123.50)	119.40 (110.45, 128.35)	−28.99 (−33.52, −24.46)	0.74	1.55 (−0.34, 3.45)	0.11
2.01 log_2_ ng/mL						−30.10 (−33.81, −26.40)		0.42 (−1.37, 2.21)	
2.78 log_2_ ng/mL						−30.53 (−33.42, −27.63)	0.72	1.08 (−0.59, 2.74)	0.06
3.56 log_2_ ng/mL						−30.96 (−34.72, −27.20)		1.74 (−0.036, 3.52)	
PFOA									
T1	139.02 (132.44, 145.59)	124.77 (118.76, 130.79)	110.53 (104.31, 116.75)	112.64 (106.41, 118.86)	114.74 (106.51, 122.97)	−28.49 (−32.87, −24.10)		1.05 (−0.85, 2.96)	
T2	141.12 (133.84, 148.40)	125.18 (118.39, 131.97)	109.24 (102.25, 116.23)	111.75 (104.72, 118.79)	114.27 (105.36, 123.17)	−31.88 (−36.28, −27.49)	0.24	1.26 (−0.65, 3.17)	0.80
T3	141.72 (134.36, 149.07)	126.11 (119.09, 133.13)	110.50 (103.16, 117.85)	112.30 (104.62, 119.98)	114.09 (104.43, 123.76)	−31.21 (−35.60, −26.83)	0.35	0.90 (−1.02, 2.82)	0.85
0.38 log_2_ ng/mL						−28.71 (−32.42, −25.00)		1.17 (−0.62, 2.97)	
1.16 log_2_ ng/mL						−30.49 (−33.40, −27.58)	0.13	1.08 (−0.61, 2.76)	0.07
1.94 log_2_ ng/mL						−32.28 (−36.00, −28.55)		0.98 (−0.84, 2.80)	
PFNA									
T1	143.52 (136.75, 150.28)	127.83 (121.61, 134.05)	112.14 (105.73, 118.55)	112.93 (106.61, 119.25)	113.71 (105.51, 121.92)	−31.37 (−35.74, −27.01)		0.39 (−1.50, 2.28)	
T2	135.18 (127.94, 142.42)	120.07 (113.26, 126.88)	104.97 (97.93, 112.00)	107.30 (110.06, 114.53)	109.62 (100.46, 118.79)	−30.21 (−34.52, −25.91)	0.69	1.17 (−0.73, 3.06)	0.34
T3	140.00 (133.20, 146.80)	124.76 (118.39, 131.12)	109.51 (102.82, 116.20)	112.00 (105.18, 118.81)	114.48 (105.75, 123.21)	−30.49 (−34.93, −26.05)	0.77	1.24 (−0.63, 3.11)	0.28
−1.21 log_2_ ng/mL						−30.66 (−34.41, −26.90)		0.90 (−0.89, 2.70)	
−0.55 log_2_ ng/mL						−30.60 (−33.51, −27.68)	0.96	1.01 (−0.67, 2.70)	0.66
0.12 log_2_ ng/mL						−30.54 (−34.32, −26.76)		1.12 (−0.67, 2.91)	
PFDA									
T1	141.88 (135.31, 148.44)	127.09 (121.02, 133.16)	112.30 (105.97, 118.64)	114.18 (107.83, 120.54)	116.06 (107.75, 124.37)	−29.57 (−33.95, −25.20)		0.94 (−0.95, 2.83)	
T2	138.69 (131.53, 145.84)	123.74 (117.08, 130.39)	108.78 (101.96, 115.61)	112.39 (105.49, 119.28)	115.99 (107.15, 124.83)	−29.90 (−34.22, −25.59)	0.91	1.80 (−0.13, 3.73)	0.29
T3	138.89 (131.68, 146.09)	122.71 (115.95, 129.47)	106.54 (99.53, 113.55)	107.66 (100.54, 114.77)	108.77 (99.86, 117.69)	−32.35 (−36.73, −27.97)	0.34	0.56 (−1.29, 2.41)	0.62
−3.24 log_2_ ng/mL						−30.23 (−33.96, −26.50)		1.17 (−0.63, 2.69)	
−2.56 log_2_ ng/mL						−30.64 (−33.54, −27.73)	0.73	1.01 (−0.67, 2.69)	0.36
−1.89 log_2_ ng/mL						−31.04 (−34.77, −27.30)		0.86 (−0.91, 2.63)	
PFHpA									
T1	137.64 (130.99, 144.30)	123.85 (117.69, 130.01)	110.05 (103.62, 116.48)	111.88 (105.22, 118.54)	113.71 (104.97, 122.46)	−27.59 (−32.02, −23.16)		0.92 (−0.99, 2.82)	
T2	139.32 (132.33, 146.31)	123.83 (117.32, 130.35)	108.35 (101.63, 115.07)	111.36 (104.61, 118.10)	114.36 (105.78, 122.94)	−30.97 (−35.24, −26.70)	0.24	1.50 (−0.36, 3.37)	0.46
T3	145.92 (138.75, 153.10)	129.23 (122.54, 135.91)	112.53 (105.66, 119.40)	113.19 (106.44, 119.94)	113.85 (105.31, 122.38)	−33.39 (−37.72, −29.06)	**0.05**	0.33 (−1.57, 2.23)	0.46
−4.85 log_2_ ng/mL						−28.01 (−31.74, −24.27)		1.12 (−0.65, 2.90)	
−3.51 log_2_ ng/mL						−30.63 (−33.51, −27.74)	**0.03**	0.98 (−0.69, 2.64)	**0.005**
−2.18 log_2_ ng/mL						−33.23 (−36.92, −29.53)		0.83 (−0.98, 2.63)	
PFUnDA									
T1	143.37 (136.58, 150.17)	127.33 (121.03, 133.63)	111.28 (104.81, 117.76)	112.84 (106.43, 119.25)	114.40 (106.12, 122.68)	−32.09 (−36.26, −27.91)		0.78 (−1.10, 2.66)	
T2	137.28 (130.29, 144.26)	122.76 (116.26, 129.26)	108.24 (101.46, 115.02)	111.00 (104.04, 117.96)	113.76 (104.73, 122.78)	−29.03 (−33.57, −24.50)	0.29	1.38 (−0.58, 3.34)	0.46
T3	140.55 (133.45, 147.64)	125.30 (118.67, 131.93)	110.05 (103.16, 116.94)	111.93 (104.93, 118.93)	113.80 (104.95, 122.65)	−30.50 (−34.93, −26.06)	0.58	0.94 (−0.93, 2.80)	0.84
−5.07 log_2_ ng/mL						−31.64 (−35.34, −27.94)		0.85 (−0.97, 2.66)	
−4.15 log_2_ ng/mL						−30.60 (−33.51, −27.69)	0.38	0.99 (−0.70, 2.67)	0.15
−3.23 log_2_ ng/mL						−29.55 (−33.31, −25.80)		1.13 (−0.65, 2.90)	
Sum carboxylic acids									
T1	139.26 (132.69, 145.83)	125.45 (119.44, 131.45)	111.63 (105.42, 117.85)	113.58 (107.44, 119.72)	115.52 (107.42, 123.63)	−27.63 (−32.02, −23.24)		0.97 (−0.93, 2.88)	
T2	140.33 (133.04, 147.62)	123.93 (117.10, 130.76)	107.53 (100.47, 114.59)	109.72 (102.47, 116.98)	111.92 (102.73, 121.11)	−32.80 (−37.19, −28.41)	0.08	1.10 (−0.81, 3.00)	0.88
T3	141.45 (134.07, 148.83)	125.76 (118.73, 132.79)	110.07 (102.74, 117.39)	111.80 (104.19, 119.41)	113.53 (103.97, 123.10)	−31.38 (−35.72, −27.05)	0.20	0.87 (−1.05, 2.78)	0.89
1.06 log_2_ ng/mL						−28.86 (−32.60, −25.13)		1.12 (−0.69, 2.92)	
1.75 log_2_ ng/mL						−30.53 (−33.44, −27.61)	0.17	1.06 (−0.63, 2.75)	0.11
2.43 log_2_ ng/mL						−32.17 (−35.91, −28.43)		1.01 (−0.80, 2.82)	

*Note*: Adjusted for race, sex, study site, age at baseline, and parents' income. PFAS levels reported for a participant with the following characteristics: age 19 at baseline, White, female, parent's income < $25,000, study site CIN.

^a^ For tertiles: significance test for difference from tertile 1. For log2‐PFAS: significance of the PFAS × Spline interaction term.

In addition, significant negative interactions were observed between PFHpA levels and both the first‐ and second‐period splines, indicating slower rates of waist circumference increase with higher PFHpA concentrations in both periods. In tertile analyses, participants in the highest tertile of PFHpS (tertile 3) had a rate of waist circumference increase of 2.74 cm per year (95% CI: 0.86, 4.63), compared to 0.29 cm per year (95% CI: −1.62, 2.20) in the lowest tertile (tertile 1).

### 3.5 Mixtures

**FIGURE 4:**
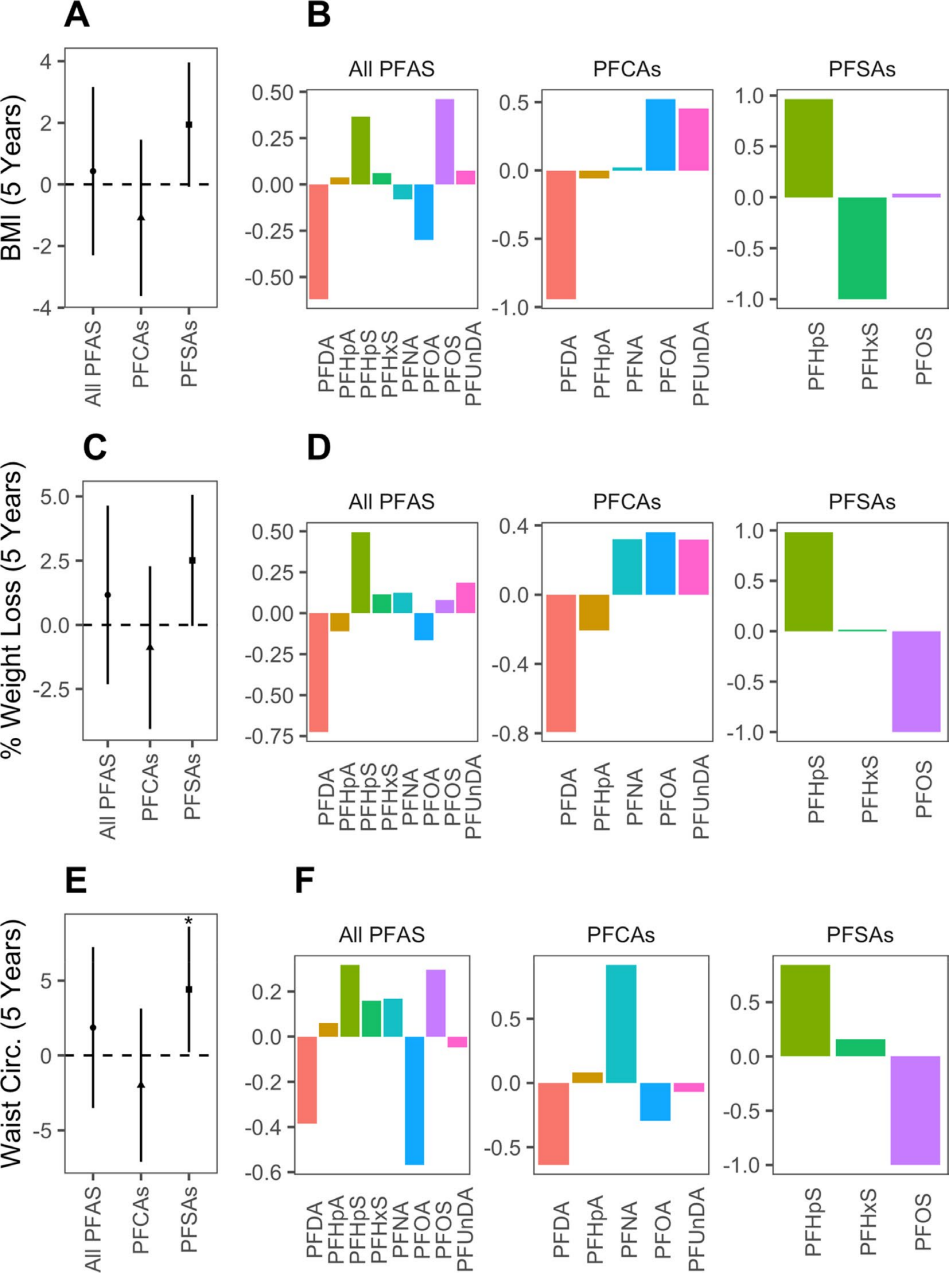
Results from quantile g‐computation assessing the relationship between baseline PFAS mixtures (all PFAS, PFCAs, and PFSAs) and (A) BMI, (C) percent weight loss, and (E) waist circumference 5 years after bariatric surgery. Coefficient plots display the Ψ and 95% CI for each outcome and mixture. Estimated weights are displayed for (B) BMI, (D) percent weight loss, and (F) waist circumference and represent the relative contribution of each PFAS to the mixture association with each outcome. Models adjusted for participants' age at baseline, sex, race, parents' income, and study site. Asterisks represent statistically significant results (*p* < 0.05). [Color figure can be viewed at wileyonlinelibrary.com]

Mixtures effects were observed for the mixture of PFSAs (PFOS, PFHxS, and PFHpS) (Figure 4 and Table S3). A one‐quartile increase in this mixture was associated with a 4.41 cm (95% CI: 0.23, 8.59, *p* = 0.041) larger waist circumference 5 years after surgery. The PFSA mixture was also positively, but not statistically significantly, associated with BMI (Ψ = 1.94, 95% CI: −0.076, 3.96, *p* = 0.061) and percent weight loss (Ψ = 2.51, 95% CI: −0.040, 5.06, *p* = 0.056) 5 years after surgery. PFHpS made the largest positive contribution to the mixture for all three outcomes, while PFOS made a negative contribution to the associations with both percent weight loss and waist circumference. The PFCA mixture was negatively associated with BMI, percent weight loss, and waist circumference 5 years post‐surgery, while the total PFAS mixture was positively associated with each 5‐year outcome (BMI [kg/m^2^], waist circumference [cm], and percent weight loss), but these associations were not statistically significant (Table S3). In both the PFCA mixture and in the mixture of all PFAS, PFDA made the largest negative contribution to the mixture.

### 3.6 Sensitivity Analyses

In the single‐chemical models, adjustment for HOMA‐IR did not appreciably change the magnitude of the interaction terms for any chemical or outcome (Figure S1). Adjustment for HOMA‐IR attenuated the effects of the PFSA mixture on BMI, waist circumference, and percent weight loss (Table S3), though the overall effects were similar.

## 4 Discussion

This study is among the first to examine the associations between exposure to PFAS and longer‐term weight outcomes following bariatric surgery in adolescents. Our findings suggest that higher levels of sulfonic acid PFAS congeners, specifically PFOS, PFHxS, and PFHpS, are associated with greater BMI regain, reduced total percent weight loss, and increased waist circumference during the 1‐ to 5‐year period after surgery. These results add to a growing body of evidence implicating PFAS exposure in weight regulation and metabolic dysregulation [9, 13, 14, 30], suggesting a role for environmental chemicals in long‐term outcomes after bariatric surgery. These findings are especially timely and significant given the growing use of weight loss interventions worldwide, emphasizing the need to understand the relationship between PFAS exposure and the successful management of weight loss [31].

The observed positive association between PFAS levels and weight regain aligns with prior studies in adults, where higher PFAS concentrations were associated with reduced weight loss and greater regain following weight loss interventions [12, 13]. Mechanistically, PFAS are known to disrupt lipid metabolism, energy homeostasis, and endocrine function, which could contribute to weight regain [14, 30, 32]. For example, PFAS have been shown to alter adipogenesis, impair mitochondrial function, and disrupt hormonal regulation, all of which may affect weight maintenance following significant initial weight loss [30, 33]. Adolescents, with their ongoing developmental and metabolic changes, may be particularly vulnerable to these effects, which warrant further investigation.

Interestingly, the strongest associations in our study were observed with PFSAs, particularly PFOS and PFHxS. These findings are consistent with experimental studies demonstrating that PFSAs have higher bioaccumulation potential and stronger effects on metabolic pathways compared to PFCAs [30, 34]. The concentration‐response relationships observed in our tertile analyses further support the hypothesis that PFAS exposure contributes to weight regain in a concentration‐dependent manner. At a PFOS concentration of 1.45 log_2_ ng/mL (2.73 ng/mL), the estimated BMI regain rate was 1.34 kg/m^2^ per year (95% CI: 0.44–2.24). Similar rates of BMI regain were observed for PFHxS, PFHpS, and the sum of the sulfonic acid congeners. Notably, in the Teen‐LABS study, exposure to PFSAs was associated with a greater BMI increase following bariatric surgery than the average BMI regain reported in a meta‐analysis of bariatric surgery outcomes [35]. For context, Shoar et al. reported an average BMI increase of approximately a 1 kg/m^2^ over a 6‐year follow‐up period among 950 adolescents who underwent bariatric surgery [35]. Therefore, the observed annual regain rates of 1.3–1.8 kg/m^2^ in our study, if sustained, could represent a clinically meaningful trend toward reversal of the metabolic benefits typically seen after surgery. For context, the Teen‐LABS study reported an average BMI reduction of 13 kg/m^2^ over a 5‐year period, equivalent to approximately 2.6 kg/m^2^ per year of weight loss in the early postoperative years [23, 28]. A regain trajectory of 1.3–1.8 kg/m^2^ per year represents a substantial proportion of this early benefit, particularly if it continues beyond the initial nadir period. Even modest increases in BMI following bariatric surgery have been associated with the recurrence of insulin resistance, dyslipidemia, and hepatic steatosis, which can significantly undermine long‐term health outcomes [36, 37].

A separate study examining the role of dietary interventions in weight control among families with obesity found that weight loss relapse was associated with PFAS exposure [13]. Higher plasma concentrations of PFOA and PFHxS were associated with greater weight gain, exceeding the effects attributed to dietary factors. These findings further support our results [13]. Notably, our study included a broader range of emerging PFAS compared to the previous study. Additionally, we observed lower PFOA concentrations but higher levels of emerging PFAS, such as PFHxS, in our study population [13]. These findings underscore the potential clinical significance of our results and highlight the importance of identifying modifiable risk factors, such as environmental exposures, that may contribute to weight regain.

The significant negative interaction observed between the time of study visit and PFHpA suggests that not all PFAS uniformly exacerbate weight outcomes. The inverse association observed between PFHpA and BMI regain was unexpected and may reflect complex pharmacokinetic or tissue‐specific mechanisms. Although PFHpA is a short‐chain PFCA with a shorter biological half‐life and lower overall bioaccumulation in plasma compared to longer‐chain PFAS, our prior research within the Teen‐LABS cohort demonstrated that PFHpA has a disproportionately high liver to plasma concentration ratio, suggesting selective hepatic sequestration [27]. This pattern differs from other PFAS congeners and may reflect unique interactions with hepatic lipid or energy metabolism. Additionally, PFHpA has been positively associated with metabolic dysfunction‐associated steatotic liver disease (MASLD) in this same cohort, indicating that its effects may be more pronounced in liver‐specific pathways. The absence of a statistically significant association for the total PFAS mixture may reflect the combined influence of PFAS congeners with opposing effects. In our individual analyses, PFSAs such as PFOS and PFHxS were positively associated with BMI regain, whereas PFHpA, a PFCA, was inversely associated with this outcome. These divergent directions of association could have attenuated the overall mixture effect in quantile g‐computation models, which estimate the joint impact of simultaneously increasing all exposures. This finding underscores the complexity of PFAS mixtures and the value of assessing both individual compounds and mixture effects to fully characterize exposure‐related health risks. These findings raise the possibility that PFHpA may influence postoperative metabolic outcomes through mechanisms distinct from other PFAS and warrant further investigation in both epidemiologic and mechanistic studies.

The stronger associations observed with PFSAs, particularly PFOS and PFHxS, compared to PFCAs may be attributed to differences in their physicochemical and toxicokinetic properties. PFSAs are generally more bioaccumulative and have longer biological half‐lives in humans, with PFOS and PFHxS estimated to persist in serum for 5–8 years and 8–10 years, respectively, compared to shorter‐chain PFCAs such as PFHpA and even PFOA [17, 38]. These characteristics increase the likelihood of sustained biological activity of PFSAs compared to PFCAs. In addition, PFSAs have been shown to exhibit stronger binding affinity to L‐FABP and PPARs, both of which play critical roles in lipid metabolism and energy balance [39, 40]. These interactions may enhance the contribution of PFSAs to metabolism disruption, including weight regain, in adolescents following bariatric surgery.

Adolescents undergoing bariatric surgery represent a particularly vulnerable population, and understanding modifiable factors that influence long‐term weight outcomes is critical. While bariatric surgery remains an effective treatment for severe obesity, weight regain can diminish the benefits of the procedure and reintroduce obesity‐related comorbidities. Environmental exposures, such as PFAS, may represent an underrecognized intervenable factor contributing to postoperative weight dynamics.

This study has several strengths, including the use of a well‐characterized cohort with longitudinal data collection and advanced statistical models to account for individual variability, time‐varying effects, and exposure mixtures. Additionally, in a previous analysis of the Teen‐LABS cohort, we found that plasma PFAS concentrations measured at the time of bariatric surgery were comparable to those reported for US adolescents in the National Health and Nutrition Examination Survey (NHANES), suggesting that our sample reflects typical background exposure levels in this age group [41]. This comparability strengthens the generalizability of our findings and supports their relevance to other adolescent populations undergoing bariatric surgery or living with severe obesity. However, some limitations should be acknowledged. First, this is an observational study, which limits causal inference. Second, while our models adjusted for key demographic and socioeconomic factors, we were unable to account for important lifestyle and clinical covariates such as dietary intake, physical activity, and endocrine comorbidities, including polycystic ovary syndrome (PCOS). These unmeasured factors may influence both PFAS exposure and metabolic outcomes, and thus their omission may contribute to residual confounding. However, based on existing literature [9, 13, 30, 42], we do not believe that these unmeasured confounders would substantially alter the direction or magnitude of the observed associations. The findings from our sensitivity analysis including HOMA‐IR as a covariate also did not indicate that adjusting for insulin sensitivity appreciably changes the results. Nonetheless, the findings should be interpreted with caution, and future studies with more comprehensive covariate data are needed to further elucidate these relationships.

In light of these findings, targeted strategies to reduce PFAS exposure may be particularly important for adolescents undergoing bariatric surgery. Specific interventions could include dietary modifications that limit the intake of highly processed and packaged foods, which are known sources of PFAS due to food contact materials [43, 44]. Encouraging the use of PFAS‐free cookware, avoiding microwaveable popcorn and other grease‐resistant food packaging, and choosing fresh or frozen foods over takeout and prepackaged meals may also help reduce exposure [43, 45]. In addition, public health efforts to regulate PFAS in drinking water and advocate for clearer labeling of PFAS‐containing products can further support exposure reduction at the population level [46]. These interventions may help mitigate environmental contributions to postoperative weight regain and promote sustained health benefits after bariatric surgery. Future research should explore the mechanisms by which PFAS exposure influences weight dynamics and should evaluate interventions to mitigate these effects. Longitudinal studies with repeated measures of PFAS and detailed assessments of metabolic pathways and dietary confounders could provide further insights. Additionally, strategies to reduce PFAS exposure, particularly in vulnerable populations, should be prioritized as a potential public health intervention addressing a variety of adverse health outcomes.

In conclusion, our findings suggest that baseline PFAS exposure is associated with longer‐term weight regain and metabolic outcomes in adolescents following bariatric surgery. These results highlight the importance of considering environmental exposures in the management of severe obesity and underscore the need for targeted interventions to improve long‐term weight maintenance in this high‐risk population.

## Author Contributions

Conceptualization and supervision of study: Brittney O. Baumert, Elizabeth Costello, David V. Conti, and Lida Chatzi. Data curation, methodology, visualization, writing, review, and editing: All authors. Writing, original draft: Brittney O. Baumert, Elizabeth Costello, and Lida Chatzi. The Teen LABS research cohort and study management, including specimen collection and banking, and manuscript review and editing were performed by Thomas Inge, Justin R. Ryder, Todd Jenkins, Stavra A. Xanthakos, and Stephanie Sisley.

## Conflicts of Interest

Dr. Scott M. Bartell and Dr. Lida Chatzi have provided expert assistance in legal cases involving PFAS‐exposed populations. Dr. Justin R. Ryder receives support from Boehringer Ingelheim Pharmaceuticals in the form of drug/placebo and serves on an advisory board for Calorify. Dr. Thomas Inge has received consulting fees from Standard Bariatrics, Teleflex, Medtronic, Mediflix, and Independent Medical Expert Consulting Services and royalties from Wolters Kluwer (UpToDate), all unrelated to this project. The other authors declare no conflicts of interest.

## Supporting information


Data S1:

## Data Availability

The data that support the findings of this study are available on request from the corresponding author. The data are not publicly available due to privacy or ethical restrictions.
