# Supplementary material for: PFAS Exposure and Postoperative Weight Regain in Adolescents After Bariatric Surgery: Findings From the Teen‐LABS Study
Source: Obesity (Silver Spring). 2025 Aug 14;33(10):1930–54. doi: 10.1002/oby.70009 (PMC12424438; doi:10.1002/oby.70009)
Supplement: Supplementary file 1 — Data S1: [file OBY-33-1930-s001.docx]

Supplementary Tables and Figures

Figure S1. Coefficient plots for the interaction terms for each PFAS with Period 1(0-1 yr post bariatric surgery, left) and Period 2 (1-3 yrs post bariatric surgery, right) spline.

Figure S2. Mean predicted BMI at baseline and post-surgery, stratified by PFAS tertiles.

Figure S3. Mean predicted percent weight loss at baseline and post-surgery, stratified by PFAS tertiles.

Figure S4. Mean predicted waist circumference at baseline and post-surgery, stratified by PFAS tertiles.

Table S1. PFAS concentrations and tertiles at baseline.

Table S2. PFAS tertile and quartile ranges.

Table S3. Linear mixed model outputs for each PFAS congener (in tertiles) on BMI.

Table S4. Linear mixed model outputs for each PFAS congener (in tertiles) on percent weight loss.

Table S5. Linear mixed model outputs for each PFAS congener (in tertiles) on waist circumference.

Table S6. Linear mixed model outputs for each log_2_-PFAS congener on BMI.

Table S7. Linear mixed model outputs for each log_2_-PFAS congener on percent weight loss.

Table S8. Linear mixed model outputs for each log_2_-PFAS congener on waist circumference.

Table S9. Results from quantile g-computation for each PFAS mixture and outcome five years after surgery.


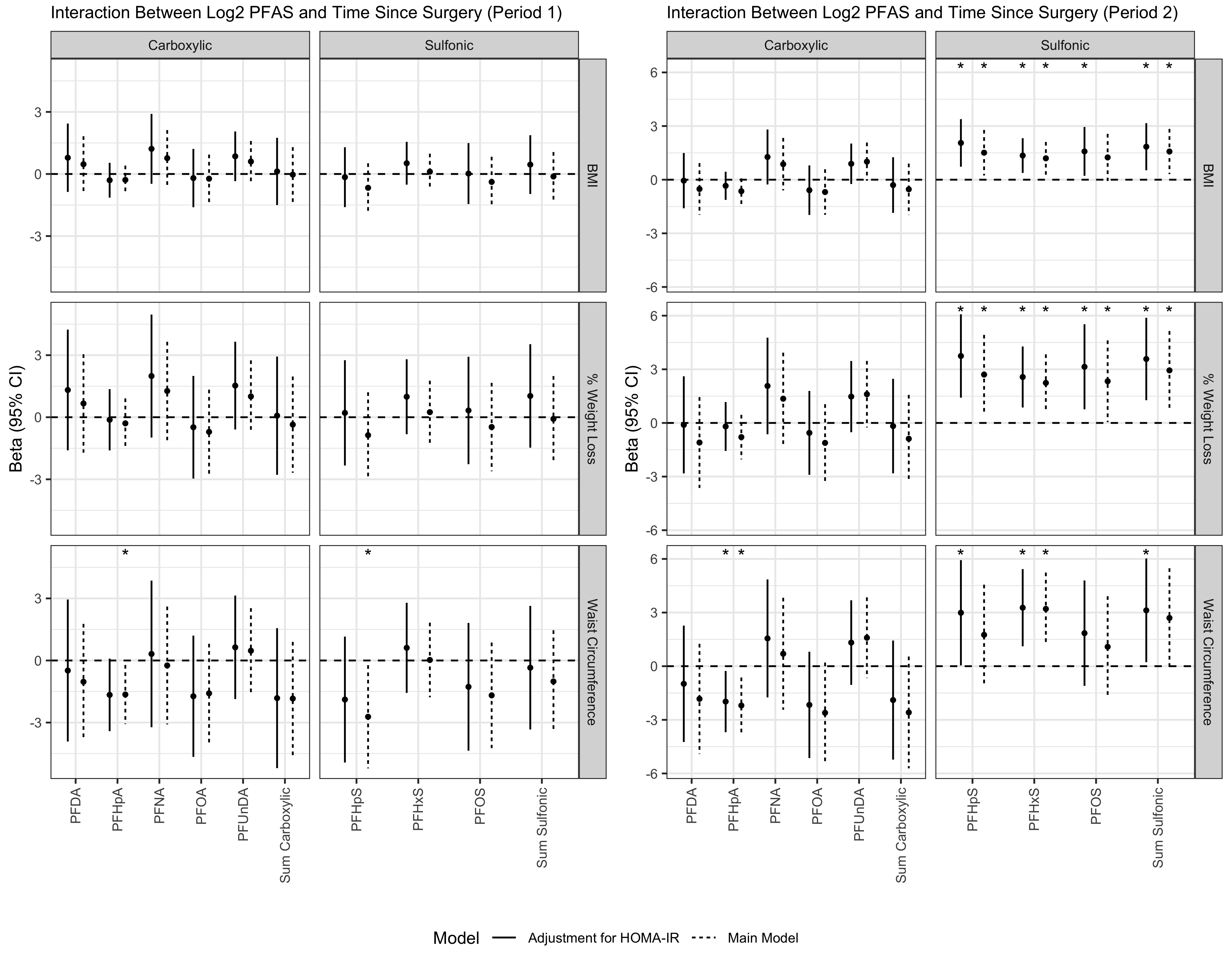


Figure S1. Coefficient plots for the interaction terms for each PFAS Period 1(0-1 yr post bariatric surgery, left) and Period 2 (1-3 yrs post bariatric surgery, right) spline. An asterisk indicates statistical significance (p < 0.05). Dashed lines represent the main model, adjusted for age, sex, race, parents’ income, and study side. Solid lines represent a model adjusted for the same covariates as the main model, with the addition of HOMA-IR.


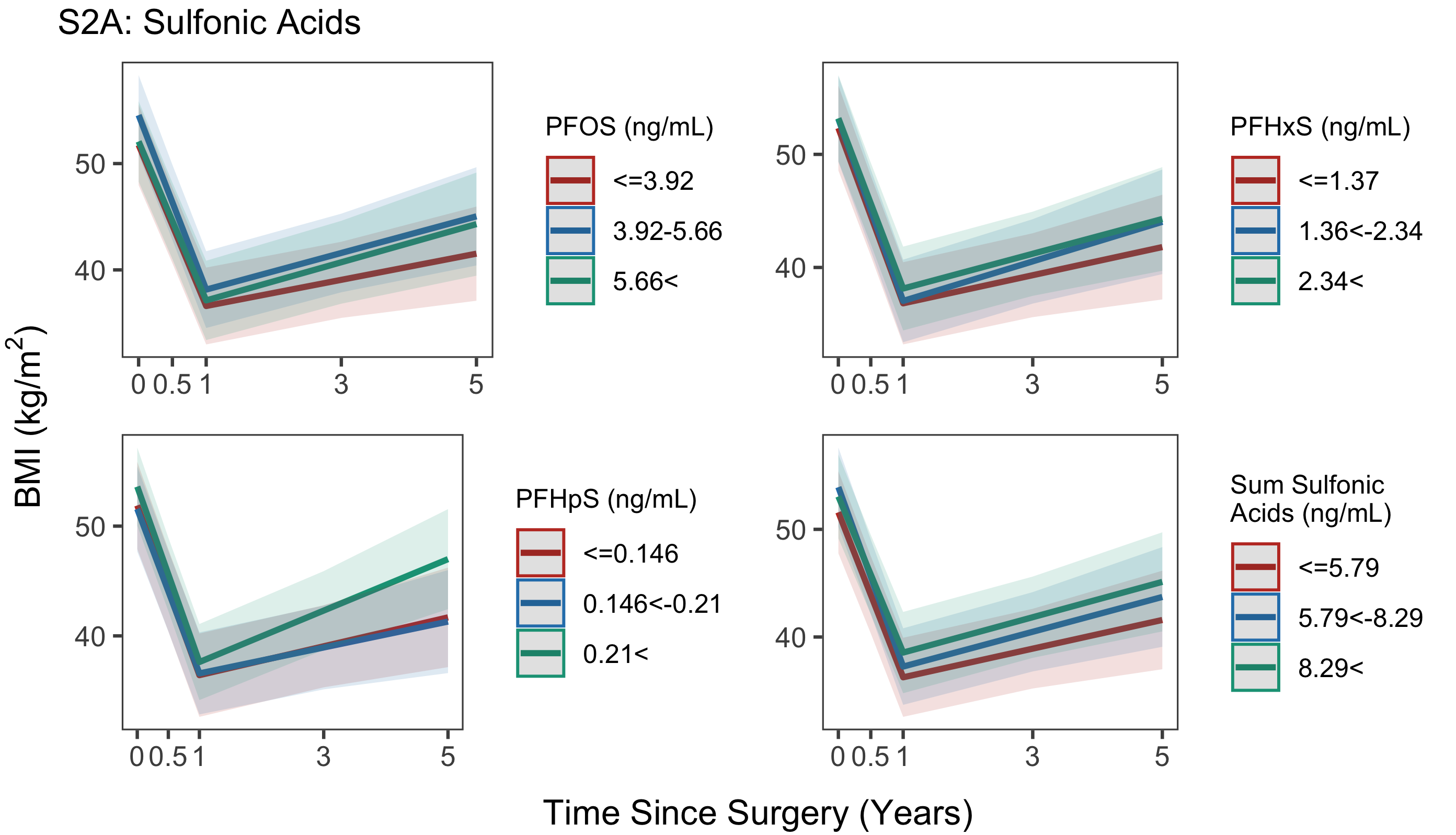


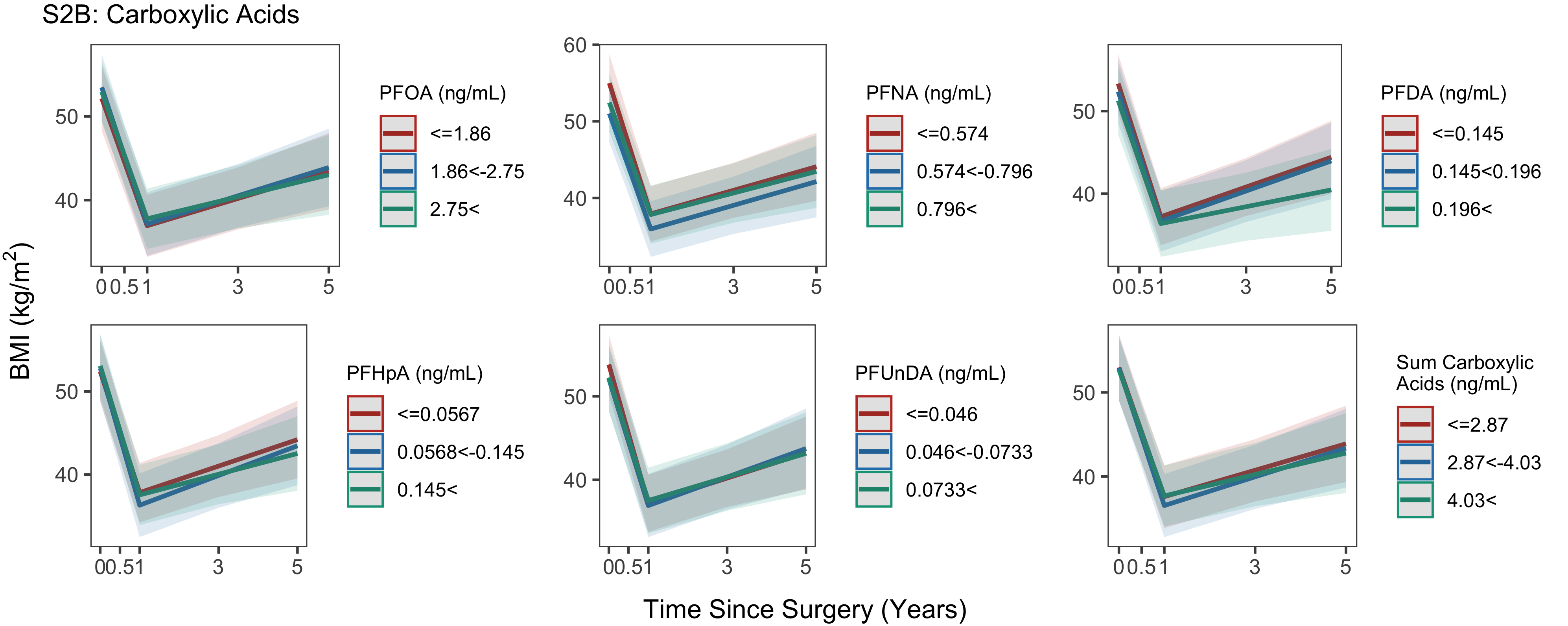


Figure S2. Mean predicted BMI at baseline and post-surgery. Trajectories are stratified PFAS tertiles for sulfonic acid congeners (A) and carboxylic acid congeners (B).


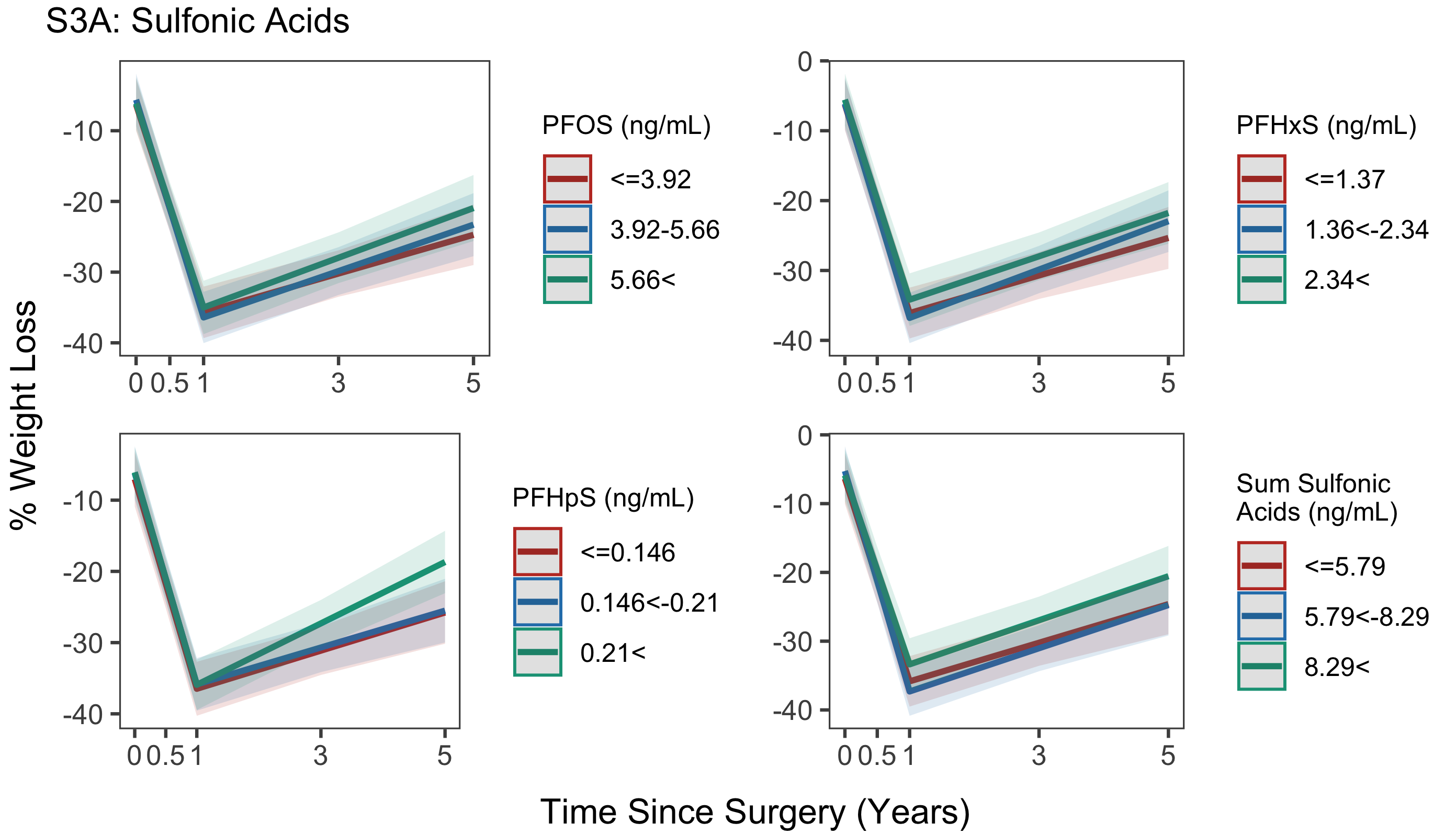


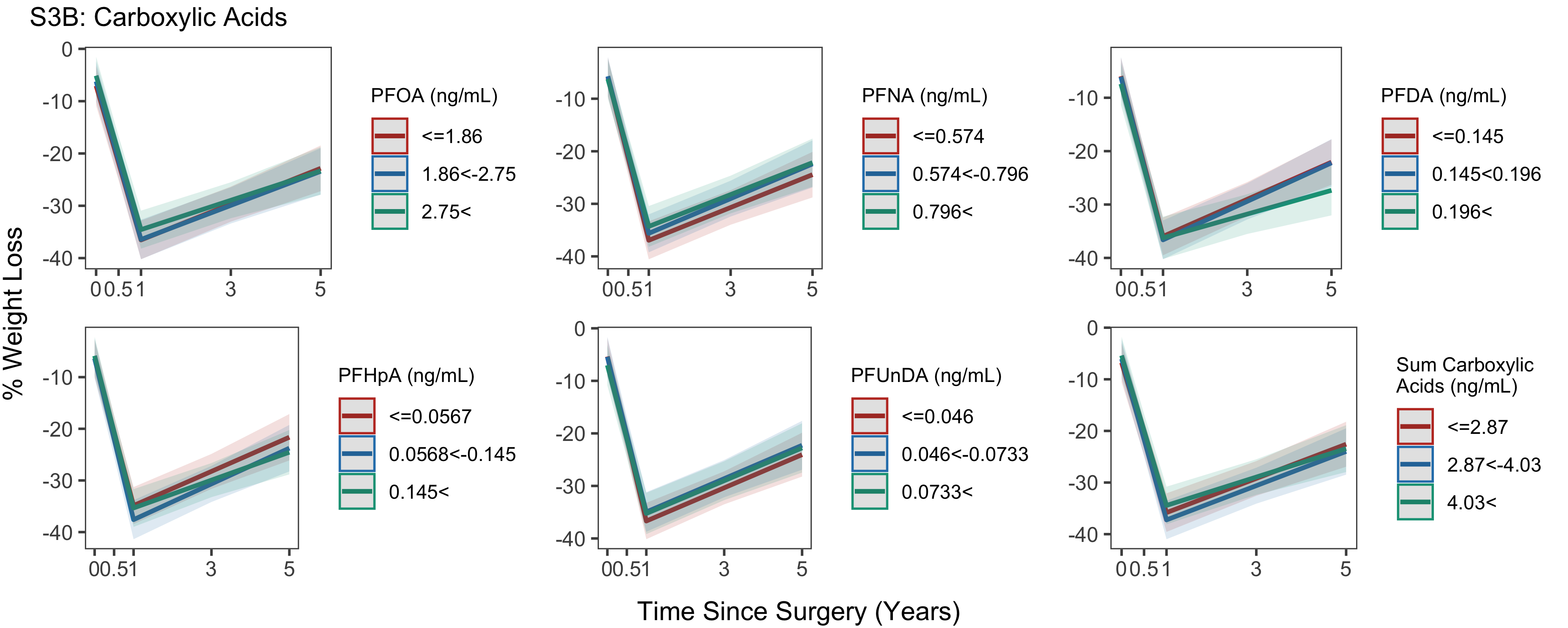


Figure S3. Mean predicted percent weight loss at baseline and post-surgery. Trajectories are stratified PFAS tertiles for sulfonic acid congeners (A) and carboxylic acid congeners (B).


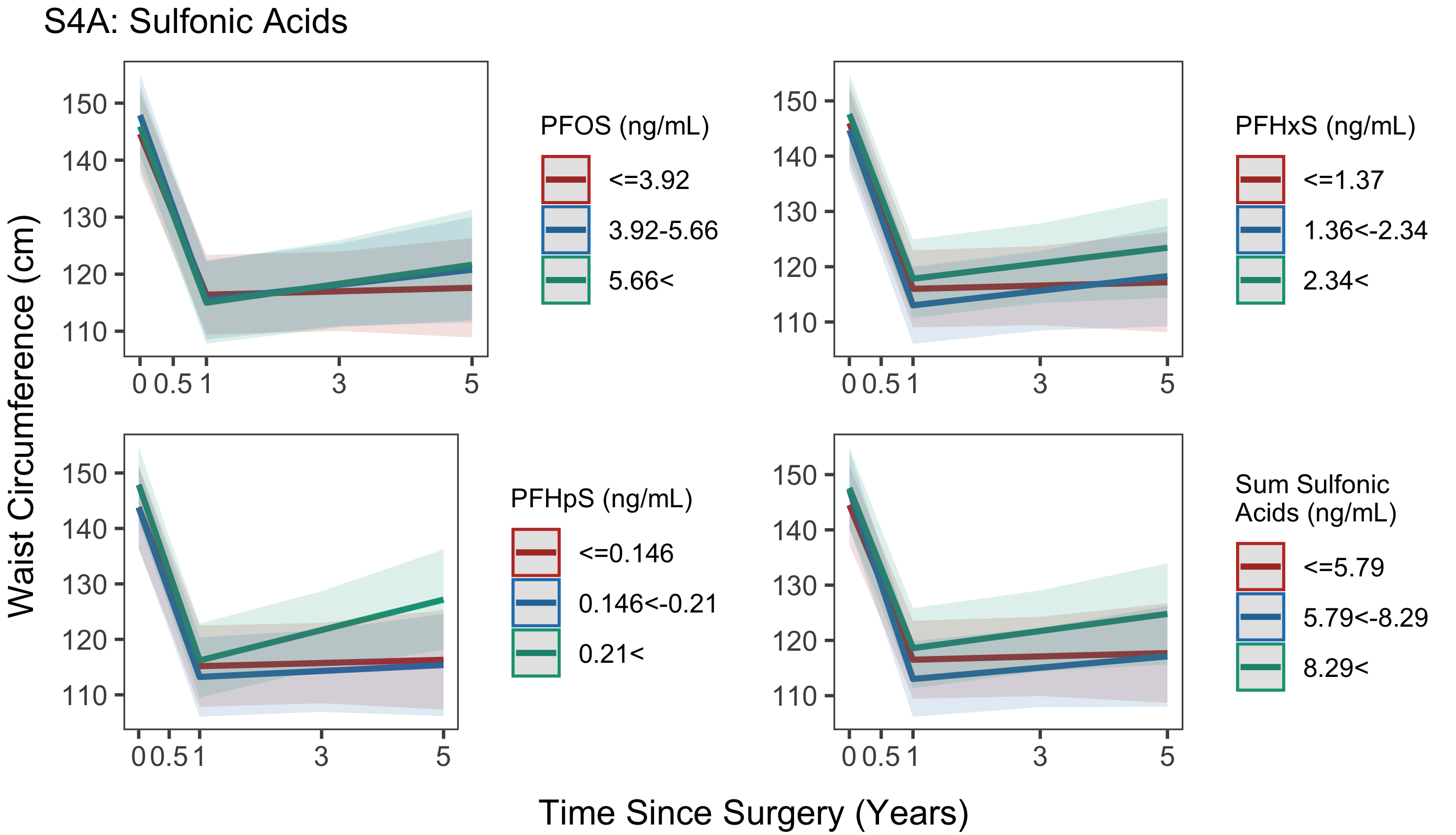


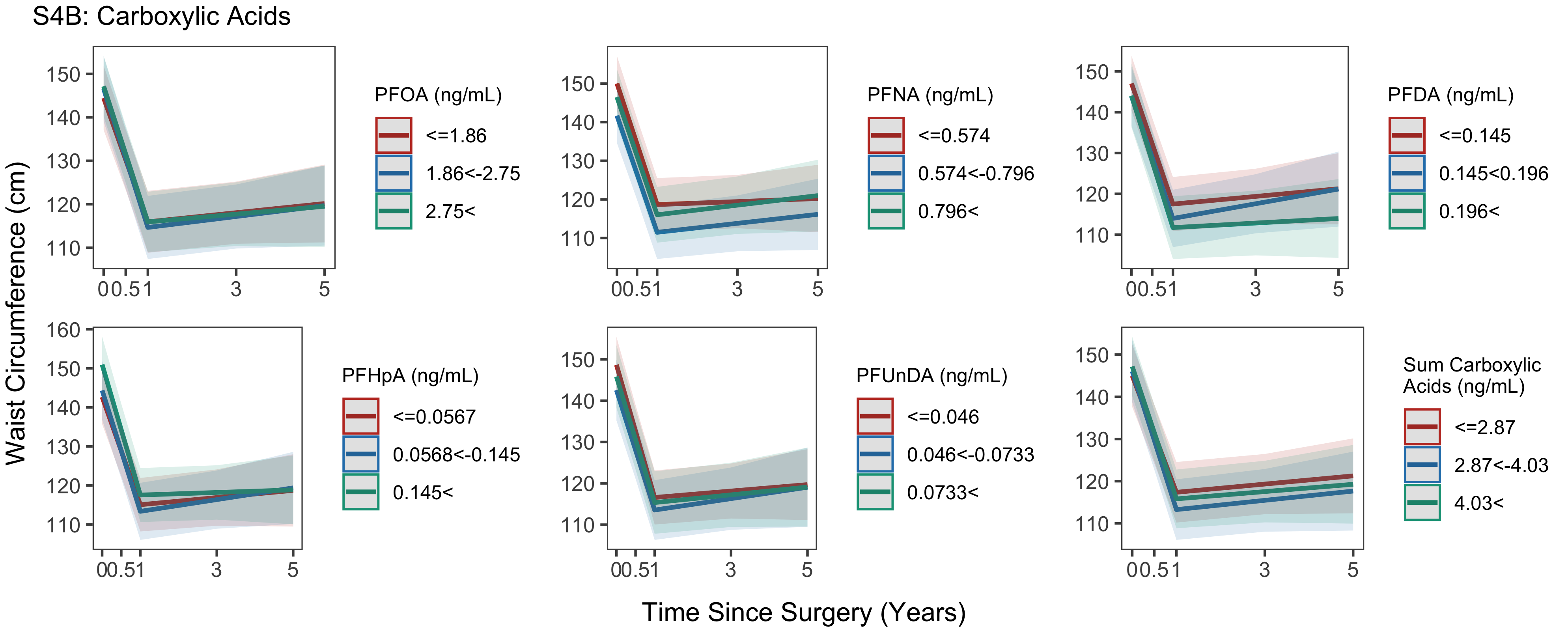


Figure S4. Mean predicted waist circumference at baseline and post-surgery. Trajectories are stratified PFAS tertiles for sulfonic acid congeners (A) and carboxylic acid congeners (B).

Table S1. PFAS concentrations and tertiles at baseline.

|  |  |  | Tertile 1 | Tertile 2 | Tertile 3 |
| --- | --- | --- | --- | --- | --- |
| PFAS (ng/mL) | Mean (SD) | Median (Range) | Range (%) | Range (%) | Range (%) |
| PFOS | 5.25 (2.68) | 4.77 (1.29-15.6) | <LOD-3.92] (33.3%) | (3.92,5.66] (33.3%) | > 5.66 (33.3%) |
| PFHxS | 2.54 (2.40) | 1.77 (0.50-14.7) | <LOD-1.35] (33.3%) | (1.35,2.34] (33.3%) | > 2.34 (33.3%) |
| PFHpS | 0.20 (0.10) | 0.18 (0.034 -0.61) | <LOD-0.146] (33.9%) | (0.146,0.21] (33.3%) | > 0.21 (32.8%) |
| Sum Sulfonic Acids | 7.99 (4.46) | 7.05 (1.92-28.4) | <LOD-5.79] (33.3%) | (5.79,8.29] (33.3%) | > 8.29 (33.3%) |
| PFOA | 2.70 (2.01) | 2.33 (0.60-15.9) | <LOD-1.86] (33.3%) | (1.86,2.75] (33.3%) | > 2.75 (33.3%) |
| PFNA | 0.77 (0.42) | 0.68 (0.21-3.14) | <LOD-0.574] (33.3%) | (0.574,0.796] (33.9%) | > 0.796 (32.8%) |
| PFDA | 0.19 (0.13) | 0.17 (0.063-1.20) | <LOD-0.145] (33.9%) | (0.145,0.196] (32.8%) | > 0.196 (33.3%) |
| PFHpA | 0.14 (0.23) | 0.087 (0.015-2.81) | <LOD-0.0567] (33.3%) | (0.0567,0.145] (33.9%) | > 0.145 (32.8%) |
| PFUnDA | 0.068 (0.044) | 0.056 (0.015-0.29) | <LOD-0.046] (34.9%) | (0.046,0.0733] (31.7%) | > 0.0733 (33.3%) |
| Sum Carboxylic Acids | 3.88 (2.34) | 3.40 (1.05-17.4) | <LOD-2.87] (33.3%) | (2.87,4.03] (33.3%) | > 4.03 (33.3%) |

Table S2. PFAS quartile ranges (ng/mL).

| **PFAS** | **Quartile 1  (n)** | **Quartile 2  (n)** | **Quartile 3  (n)** | **Quartile 4  (n)** |
| --- | --- | --- | --- | --- |
| PFOA | ≤ 1.65  (47) | 1.65 < - 2.33  (46) | 2.33 < - 3.00  (46) | 3.00 < - 15.9  (47) |
| PFOS | ≤ 3.45  (47) | 3.45 < - 4.77  (46) | 4.77 < -6.32  (46) | 6.32 < - 15.6  (47) |
| PFHxS | ≤ 1.24  (47) | 1.24 < -1.77  (46) | 1.77 < -2.86  (46) | 2.86 < - 14.7  (47) |
| PFNA | ≤ 0.524  (48) | 0.524 < - 0.676  (46) | 0.676 < - 0.907  (45) | 0.907 < - 3.14  (47) |
| PFDA | ≤ 0.129  (47) | 0.129 < - 0.166  (47) | 0.166 < - 0.214  (46) | 0.214 < - 1.2  (46) |
| PFHpS | ≤ 0.129  (47) | 0.129 < - 0.178  (47) | 0.177 < -0.244  (46) | 0.244 < - 0.612  (46) |
| PFHpA | ≤ 0.049  (48) | 0.049 < - 0.0865  (45) | 0.0865 < - 0.176  (46) | 0.176 <- 2.81  (47) |
| PFUnDA | ≤ 0.041  (49) | 0.041 < - 0.0555  (44) | 0.0555 < - 0.0838  (46) | 0.0838 < - 0.285  (47) |

| Table S3. Linear mixed model outputs for each PFAS congener (in tertiles) on BMI. | | | | | | |
| --- | --- | --- | --- | --- | --- | --- |
|  | Term | Value | Std.Error | DF | t-value | p-value |
| PFOS | (Intercept) | 60.749 | 7.410 | 526 | 8.198 | 1.877E-15 |
|  | total_pfos_targeted_plasma_tert3.92-5.66 | 2.802 | 1.724 | 169 | 1.625 | 1.060E-01 |
|  | total_pfos_targeted_plasma_tert5.66< | 0.324 | 1.746 | 169 | 0.186 | 8.529E-01 |
|  | bSpline(visit_num, knots = 1, degree = 1)1 | -15.151 | 1.077 | 526 | -14.071 | 2.142E-38 |
|  | bSpline(visit_num, knots = 1, degree = 1)2 | -10.229 | 2.281 | 526 | -4.485 | 8.953E-06 |
|  | sexFemale | -0.927 | 1.493 | 169 | -0.621 | 5.357E-01 |
|  | race_binaryOther | 5.309 | 1.442 | 169 | 3.682 | 3.107E-04 |
|  | ageyrs | -0.381 | 0.409 | 526 | -0.930 | 3.529E-01 |
|  | site_binary | -3.907 | 1.314 | 169 | -2.973 | 3.383E-03 |
|  | parents_income25000-74999 | -3.863 | 1.469 | 169 | -2.629 | 9.355E-03 |
|  | parents_income75000 or more | -6.243 | 1.729 | 169 | -3.611 | 4.022E-04 |
|  | total_pfos_targeted_plasma_tert3.92-5.66:bSpline(visit_num, knots = 1, degree = 1)1 | -1.260 | 1.382 | 526 | -0.911 | 3.625E-01 |
|  | total_pfos_targeted_plasma_tert5.66<:bSpline(visit_num, knots = 1, degree = 1)1 | 0.203 | 1.413 | 526 | 0.144 | 8.859E-01 |
|  | total_pfos_targeted_plasma_tert3.92-5.66:bSpline(visit_num, knots = 1, degree = 1)2 | 0.710 | 1.276 | 526 | 0.557 | 5.780E-01 |
|  | total_pfos_targeted_plasma_tert5.66<:bSpline(visit_num, knots = 1, degree = 1)2 | 2.455 | 1.275 | 526 | 1.926 | 5.470E-02 |
| PFHxS | (Intercept) | 60.121 | 7.493 | 526 | 8.023 | 6.740E-15 |
|  | pf_hx_s_targeted_plasma_tert1.36<-2.34 | 0.804 | 1.740 | 169 | 0.462 | 6.445E-01 |
|  | pf_hx_s_targeted_plasma_tert2.34< | 0.864 | 1.738 | 169 | 0.497 | 6.199E-01 |
|  | bSpline(visit_num, knots = 1, degree = 1)1 | -15.501 | 1.087 | 526 | -14.264 | 2.931E-39 |
|  | bSpline(visit_num, knots = 1, degree = 1)2 | -10.525 | 2.284 | 526 | -4.609 | 5.095E-06 |
|  | sexFemale | -0.918 | 1.501 | 169 | -0.612 | 5.417E-01 |
|  | race_binaryOther | 5.191 | 1.457 | 169 | 3.564 | 4.753E-04 |
|  | ageyrs | -0.320 | 0.407 | 526 | -0.786 | 4.322E-01 |
|  | site_binary | -3.820 | 1.318 | 169 | -2.898 | 4.256E-03 |
|  | parents_income25000-74999 | -3.711 | 1.480 | 169 | -2.508 | 1.309E-02 |
|  | parents_income75000 or more | -6.274 | 1.715 | 169 | -3.658 | 3.394E-04 |
|  | pf_hx_s_targeted_plasma_tert1.36<-2.34:bSpline(visit_num, knots = 1, degree = 1)1 | -0.591 | 1.378 | 526 | -0.429 | 6.681E-01 |
|  | pf_hx_s_targeted_plasma_tert2.34<:bSpline(visit_num, knots = 1, degree = 1)1 | 0.445 | 1.433 | 526 | 0.310 | 7.564E-01 |
|  | pf_hx_s_targeted_plasma_tert1.36<-2.34:bSpline(visit_num, knots = 1, degree = 1)2 | 1.427 | 1.270 | 526 | 1.123 | 2.618E-01 |
|  | pf_hx_s_targeted_plasma_tert2.34<:bSpline(visit_num, knots = 1, degree = 1)2 | 1.623 | 1.295 | 526 | 1.254 | 2.104E-01 |
| PFHpS | (Intercept) | 61.669 | 7.345 | 526 | 8.396 | 4.315E-16 |
|  | pf_hp_s_targeted_plasma_tert0.146<-0.21 | -0.322 | 1.748 | 169 | -0.184 | 8.542E-01 |
|  | pf_hp_s_targeted_plasma_tert0.21< | 1.714 | 1.779 | 169 | 0.963 | 3.368E-01 |
|  | bSpline(visit_num, knots = 1, degree = 1)1 | -15.465 | 1.094 | 526 | -14.141 | 1.036E-38 |
|  | bSpline(visit_num, knots = 1, degree = 1)2 | -10.198 | 2.271 | 526 | -4.491 | 8.719E-06 |
|  | sexFemale | -0.390 | 1.523 | 169 | -0.256 | 7.982E-01 |
|  | race_binaryOther | 5.293 | 1.451 | 169 | 3.648 | 3.513E-04 |
|  | ageyrs | -0.426 | 0.406 | 526 | -1.049 | 2.949E-01 |
|  | site_binary | -3.754 | 1.306 | 169 | -2.875 | 4.558E-03 |
|  | parents_income25000-74999 | -3.751 | 1.460 | 169 | -2.570 | 1.102E-02 |
|  | parents_income75000 or more | -6.671 | 1.727 | 169 | -3.863 | 1.592E-04 |
|  | pf_hp_s_targeted_plasma_tert0.146<-0.21:bSpline(visit_num, knots = 1, degree = 1)1 | 0.483 | 1.381 | 526 | 0.350 | 7.267E-01 |
|  | pf_hp_s_targeted_plasma_tert0.21<:bSpline(visit_num, knots = 1, degree = 1)1 | -0.517 | 1.404 | 526 | -0.368 | 7.127E-01 |
|  | pf_hp_s_targeted_plasma_tert0.146<-0.21:bSpline(visit_num, knots = 1, degree = 1)2 | -0.063 | 1.278 | 526 | -0.049 | 9.608E-01 |
|  | pf_hp_s_targeted_plasma_tert0.21<:bSpline(visit_num, knots = 1, degree = 1)2 | 3.590 | 1.269 | 526 | 2.830 | 4.832E-03 |
| Sum Sulfonic Acids | (Intercept) | 59.929 | 7.426 | 526 | 8.070 | 4.784E-15 |
|  | sum_sulfonic_tert5.79<-8.29 | 2.333 | 1.757 | 169 | 1.328 | 1.860E-01 |
|  | sum_sulfonic_tert8.29< | 1.471 | 1.747 | 169 | 0.842 | 4.011E-01 |
|  | bSpline(visit_num, knots = 1, degree = 1)1 | -15.329 | 1.095 | 526 | -14.003 | 4.303E-38 |
|  | bSpline(visit_num, knots = 1, degree = 1)2 | -10.003 | 2.278 | 526 | -4.392 | 1.362E-05 |
|  | sexFemale | -0.740 | 1.503 | 169 | -0.492 | 6.233E-01 |
|  | race_binaryOther | 5.191 | 1.459 | 169 | 3.559 | 4.836E-04 |
|  | ageyrs | -0.343 | 0.406 | 526 | -0.844 | 3.988E-01 |
|  | site_binary | -4.060 | 1.327 | 169 | -3.060 | 2.576E-03 |
|  | parents_income25000-74999 | -3.903 | 1.473 | 169 | -2.650 | 8.808E-03 |
|  | parents_income75000 or more | -6.623 | 1.731 | 169 | -3.827 | 1.823E-04 |
|  | sum_sulfonic_tert5.79<-8.29:bSpline(visit_num, knots = 1, degree = 1)1 | -1.336 | 1.379 | 526 | -0.969 | 3.329E-01 |
|  | sum_sulfonic_tert8.29<:bSpline(visit_num, knots = 1, degree = 1)1 | 0.827 | 1.432 | 526 | 0.578 | 5.638E-01 |
|  | sum_sulfonic_tert5.79<-8.29:bSpline(visit_num, knots = 1, degree = 1)2 | -0.196 | 1.281 | 526 | -0.153 | 8.783E-01 |
|  | sum_sulfonic_tert8.29<:bSpline(visit_num, knots = 1, degree = 1)2 | 2.070 | 1.275 | 526 | 1.623 | 1.052E-01 |
| PFOA | (Intercept) | 60.429 | 7.484 | 526 | 8.075 | 4.634E-15 |
|  | pfoa_targeted_plasma_tert1.86<-2.75 | 1.311 | 1.766 | 169 | 0.743 | 4.587E-01 |
|  | pfoa_targeted_plasma_tert2.75< | 0.785 | 1.812 | 169 | 0.433 | 6.654E-01 |
|  | bSpline(visit_num, knots = 1, degree = 1)1 | -15.210 | 1.095 | 526 | -13.894 | 1.305E-37 |
|  | bSpline(visit_num, knots = 1, degree = 1)2 | -8.707 | 2.309 | 526 | -3.772 | 1.806E-04 |
|  | sexFemale | -0.963 | 1.521 | 169 | -0.633 | 5.277E-01 |
|  | race_binaryOther | 5.077 | 1.447 | 169 | 3.509 | 5.763E-04 |
|  | ageyrs | -0.343 | 0.412 | 526 | -0.833 | 4.052E-01 |
|  | site_binary | -3.869 | 1.357 | 169 | -2.852 | 4.892E-03 |
|  | parents_income25000-74999 | -3.651 | 1.496 | 169 | -2.440 | 1.570E-02 |
|  | parents_income75000 or more | -6.190 | 1.756 | 169 | -3.524 | 5.460E-04 |
|  | pfoa_targeted_plasma_tert1.86<-2.75:bSpline(visit_num, knots = 1, degree = 1)1 | -1.111 | 1.406 | 526 | -0.790 | 4.298E-01 |
|  | pfoa_targeted_plasma_tert2.75<:bSpline(visit_num, knots = 1, degree = 1)1 | 0.046 | 1.402 | 526 | 0.033 | 9.736E-01 |
|  | pfoa_targeted_plasma_tert1.86<-2.75:bSpline(visit_num, knots = 1, degree = 1)2 | -0.862 | 1.289 | 526 | -0.669 | 5.039E-01 |
|  | pfoa_targeted_plasma_tert2.75<:bSpline(visit_num, knots = 1, degree = 1)2 | -1.205 | 1.279 | 526 | -0.942 | 3.467E-01 |
| PFNA | (Intercept) | 62.458 | 7.387 | 526 | 8.455 | 2.758E-16 |
|  | pfna_targeted_plasma_tert0.574<-0.796 | -3.952 | 1.745 | 169 | -2.265 | 2.479E-02 |
|  | pfna_targeted_plasma_tert0.796< | -2.536 | 1.799 | 169 | -1.409 | 1.606E-01 |
|  | bSpline(visit_num, knots = 1, degree = 1)1 | -17.065 | 1.085 | 526 | -15.726 | 5.899E-46 |
|  | bSpline(visit_num, knots = 1, degree = 1)2 | -10.905 | 2.270 | 526 | -4.804 | 2.033E-06 |
|  | sexFemale | -1.298 | 1.498 | 169 | -0.866 | 3.877E-01 |
|  | race_binaryOther | 5.042 | 1.433 | 169 | 3.519 | 5.576E-04 |
|  | ageyrs | -0.306 | 0.406 | 526 | -0.752 | 4.522E-01 |
|  | site_binary | -3.683 | 1.351 | 169 | -2.726 | 7.093E-03 |
|  | parents_income25000-74999 | -3.161 | 1.468 | 169 | -2.153 | 3.274E-02 |
|  | parents_income75000 or more | -5.420 | 1.760 | 169 | -3.079 | 2.426E-03 |
|  | pfna_targeted_plasma_tert0.574<-0.796:bSpline(visit_num, knots = 1, degree = 1)1 | 1.967 | 1.385 | 526 | 1.421 | 1.559E-01 |
|  | pfna_targeted_plasma_tert0.796<:bSpline(visit_num, knots = 1, degree = 1)1 | 2.440 | 1.420 | 526 | 1.719 | 8.628E-02 |
|  | pfna_targeted_plasma_tert0.574<-0.796:bSpline(visit_num, knots = 1, degree = 1)2 | 2.009 | 1.289 | 526 | 1.558 | 1.198E-01 |
|  | pfna_targeted_plasma_tert0.796<:bSpline(visit_num, knots = 1, degree = 1)2 | 1.914 | 1.282 | 526 | 1.492 | 1.362E-01 |
| PFDA | (Intercept) | 61.543 | 7.439 | 526 | 8.273 | 1.083E-15 |
|  | pfda_targeted_plasma_tert0.145<0.196 | -0.965 | 1.734 | 169 | -0.556 | 5.787E-01 |
|  | pfda_targeted_plasma_tert0.196< | -2.048 | 1.783 | 169 | -1.149 | 2.523E-01 |
|  | bSpline(visit_num, knots = 1, degree = 1)1 | -16.119 | 1.088 | 526 | -14.812 | 9.682E-42 |
|  | bSpline(visit_num, knots = 1, degree = 1)2 | -8.917 | 2.253 | 526 | -3.957 | 8.626E-05 |
|  | sexFemale | -0.875 | 1.498 | 169 | -0.584 | 5.598E-01 |
|  | race_binaryOther | 5.207 | 1.446 | 169 | 3.601 | 4.168E-04 |
|  | ageyrs | -0.337 | 0.405 | 526 | -0.831 | 4.064E-01 |
|  | site_binary | -4.025 | 1.324 | 169 | -3.041 | 2.733E-03 |
|  | parents_income25000-74999 | -2.942 | 1.502 | 169 | -1.959 | 5.180E-02 |
|  | parents_income75000 or more | -5.565 | 1.756 | 169 | -3.169 | 1.814E-03 |
|  | pfda_targeted_plasma_tert0.145<0.196:bSpline(visit_num, knots = 1, degree = 1)1 | 0.435 | 1.396 | 526 | 0.312 | 7.552E-01 |
|  | pfda_targeted_plasma_tert0.196<:bSpline(visit_num, knots = 1, degree = 1)1 | 1.223 | 1.404 | 526 | 0.871 | 3.843E-01 |
|  | pfda_targeted_plasma_tert0.145<0.196:bSpline(visit_num, knots = 1, degree = 1)2 | 0.518 | 1.295 | 526 | 0.400 | 6.894E-01 |
|  | pfda_targeted_plasma_tert0.196<:bSpline(visit_num, knots = 1, degree = 1)2 | -1.900 | 1.248 | 526 | -1.523 | 1.284E-01 |
| PFHpA | (Intercept) | 60.645 | 7.598 | 526 | 7.982 | 9.083E-15 |
|  | pf_hp_a_targeted_plasma_tert0.0568<-0.145 | 0.400 | 1.735 | 169 | 0.230 | 8.182E-01 |
|  | pf_hp_a_targeted_plasma_tert0.145< | 0.672 | 1.738 | 169 | 0.386 | 6.997E-01 |
|  | bSpline(visit_num, knots = 1, degree = 1)1 | -14.597 | 1.115 | 526 | -13.086 | 4.502E-34 |
|  | bSpline(visit_num, knots = 1, degree = 1)2 | -8.212 | 2.301 | 526 | -3.570 | 3.901E-04 |
|  | sexFemale | -1.018 | 1.513 | 169 | -0.673 | 5.022E-01 |
|  | race_binaryOther | 5.075 | 1.450 | 169 | 3.501 | 5.934E-04 |
|  | ageyrs | -0.343 | 0.410 | 526 | -0.837 | 4.027E-01 |
|  | site_binary | -3.802 | 1.322 | 169 | -2.877 | 4.538E-03 |
|  | parents_income25000-74999 | -3.422 | 1.470 | 169 | -2.328 | 2.110E-02 |
|  | parents_income75000 or more | -6.019 | 1.724 | 169 | -3.492 | 6.124E-04 |
|  | pf_hp_a_targeted_plasma_tert0.0568<-0.145:bSpline(visit_num, knots = 1, degree = 1)1 | -1.918 | 1.403 | 526 | -1.367 | 1.721E-01 |
|  | pf_hp_a_targeted_plasma_tert0.145<:bSpline(visit_num, knots = 1, degree = 1)1 | -0.950 | 1.412 | 526 | -0.673 | 5.014E-01 |
|  | pf_hp_a_targeted_plasma_tert0.0568<-0.145:bSpline(visit_num, knots = 1, degree = 1)2 | -1.144 | 1.289 | 526 | -0.887 | 3.753E-01 |
|  | pf_hp_a_targeted_plasma_tert0.145<:bSpline(visit_num, knots = 1, degree = 1)2 | -2.342 | 1.279 | 526 | -1.831 | 6.770E-02 |
| PFUnDA | (Intercept) | 61.840 | 7.431 | 526 | 8.322 | 7.517E-16 |
|  | pf_un_da_targeted_plasma_tert0.046<-0.0733 | -1.702 | 1.728 | 169 | -0.985 | 3.260E-01 |
|  | pf_un_da_targeted_plasma_tert0.0733< | -1.560 | 1.745 | 169 | -0.894 | 3.726E-01 |
|  | bSpline(visit_num, knots = 1, degree = 1)1 | -16.632 | 1.049 | 526 | -15.854 | 1.493E-46 |
|  | bSpline(visit_num, knots = 1, degree = 1)2 | -10.602 | 2.268 | 526 | -4.676 | 3.730E-06 |
|  | sexFemale | -0.985 | 1.507 | 169 | -0.654 | 5.140E-01 |
|  | race_binaryOther | 5.081 | 1.491 | 169 | 3.409 | 8.157E-04 |
|  | ageyrs | -0.332 | 0.408 | 526 | -0.812 | 4.171E-01 |
|  | site_binary | -3.813 | 1.338 | 169 | -2.850 | 4.917E-03 |
|  | parents_income25000-74999 | -3.456 | 1.468 | 169 | -2.355 | 1.967E-02 |
|  | parents_income75000 or more | -6.059 | 1.716 | 169 | -3.531 | 5.332E-04 |
|  | pf_un_da_targeted_plasma_tert0.046<-0.0733:bSpline(visit_num, knots = 1, degree = 1)1 | 1.425 | 1.398 | 526 | 1.019 | 3.085E-01 |
|  | pf_un_da_targeted_plasma_tert0.0733<:bSpline(visit_num, knots = 1, degree = 1)1 | 1.883 | 1.385 | 526 | 1.359 | 1.746E-01 |
|  | pf_un_da_targeted_plasma_tert0.046<-0.0733:bSpline(visit_num, knots = 1, degree = 1)2 | 2.235 | 1.292 | 526 | 1.730 | 8.429E-02 |
|  | pf_un_da_targeted_plasma_tert0.0733<:bSpline(visit_num, knots = 1, degree = 1)2 | 1.508 | 1.246 | 526 | 1.210 | 2.268E-01 |
| Sum Carboxylic Acids | (Intercept) | 60.729 | 7.447 | 526 | 8.155 | 2.588E-15 |
|  | sum_carboxylic_tert2.87<-4.03 | -0.084 | 1.792 | 169 | -0.047 | 9.626E-01 |
|  | sum_carboxylic_tert4.03< | -0.261 | 1.806 | 169 | -0.145 | 8.850E-01 |
|  | bSpline(visit_num, knots = 1, degree = 1)1 | -15.387 | 1.100 | 526 | -13.983 | 5.245E-38 |
|  | bSpline(visit_num, knots = 1, degree = 1)2 | -9.099 | 2.299 | 526 | -3.957 | 8.629E-05 |
|  | sexFemale | -1.092 | 1.528 | 169 | -0.715 | 4.758E-01 |
|  | race_binaryOther | 5.030 | 1.445 | 169 | 3.480 | 6.372E-04 |
|  | ageyrs | -0.320 | 0.411 | 526 | -0.778 | 4.369E-01 |
|  | site_binary | -3.708 | 1.347 | 169 | -2.753 | 6.553E-03 |
|  | parents_income25000-74999 | -3.384 | 1.506 | 169 | -2.247 | 2.592E-02 |
|  | parents_income75000 or more | -6.002 | 1.772 | 169 | -3.388 | 8.766E-04 |
|  | sum_carboxylic_tert2.87<-4.03:bSpline(visit_num, knots = 1, degree = 1)1 | -0.975 | 1.411 | 526 | -0.691 | 4.898E-01 |
|  | sum_carboxylic_tert4.03<:bSpline(visit_num, knots = 1, degree = 1)1 | 0.349 | 1.404 | 526 | 0.249 | 8.038E-01 |
|  | sum_carboxylic_tert2.87<-4.03:bSpline(visit_num, knots = 1, degree = 1)2 | -0.423 | 1.288 | 526 | -0.328 | 7.429E-01 |
|  | sum_carboxylic_tert4.03<:bSpline(visit_num, knots = 1, degree = 1)2 | -0.840 | 1.275 | 526 | -0.659 | 5.104E-01 |

Table S2. Linear mixed model outputs for each PFAS congener on BMI.

Table S4. Linear mixed model outputs for each PFAS congener (in tertiles) on percent weight loss.

|  | Term | Value | Std.Error | DF | t-value | p-value |
| --- | --- | --- | --- | --- | --- | --- |
| PFOS | (Intercept) | 9.430 | 6.801 | 532 | 1.387 | 1.661E-01 |
|  | total_pfos_targeted_plasma_tert3.92-5.66 | 0.704 | 1.901 | 169 | 0.370 | 7.117E-01 |
|  | total_pfos_targeted_plasma_tert5.66< | 0.184 | 1.914 | 169 | 0.096 | 9.237E-01 |
|  | bSpline(visit_num, knots = 1, degree = 1)1 | -29.369 | 1.773 | 532 | -16.562 | 5.446E-50 |
|  | bSpline(visit_num, knots = 1, degree = 1)2 | -18.397 | 2.462 | 532 | -7.473 | 3.251E-13 |
|  | sexFemale | 1.460 | 1.325 | 169 | 1.103 | 2.718E-01 |
|  | race_binaryOther | 3.303 | 1.277 | 169 | 2.586 | 1.054E-02 |
|  | ageyrs | -0.766 | 0.374 | 532 | -2.049 | 4.099E-02 |
|  | site_binary | -2.644 | 1.166 | 169 | -2.268 | 2.461E-02 |
|  | parents_income25000-74999 | 0.258 | 1.305 | 169 | 0.198 | 8.436E-01 |
|  | parents_income75000 or more | -1.031 | 1.529 | 169 | -0.674 | 5.011E-01 |
|  | total_pfos_targeted_plasma_tert3.92-5.66:bSpline(visit_num, knots = 1, degree = 1)1 | -1.428 | 2.449 | 532 | -0.583 | 5.601E-01 |
|  | total_pfos_targeted_plasma_tert5.66<:bSpline(visit_num, knots = 1, degree = 1)1 | 0.507 | 2.498 | 532 | 0.203 | 8.392E-01 |
|  | total_pfos_targeted_plasma_tert3.92-5.66:bSpline(visit_num, knots = 1, degree = 1)2 | 0.727 | 2.250 | 532 | 0.323 | 7.467E-01 |
|  | total_pfos_targeted_plasma_tert5.66<:bSpline(visit_num, knots = 1, degree = 1)2 | 3.601 | 2.247 | 532 | 1.603 | 1.096E-01 |
| PFHxS | (Intercept) | 8.076 | 6.803 | 532 | 1.187 | 2.357E-01 |
|  | pf_hx_s_targeted_plasma_tert1.36<-2.34 | 0.146 | 1.897 | 169 | 0.077 | 9.386E-01 |
|  | pf_hx_s_targeted_plasma_tert2.34< | 0.726 | 1.911 | 169 | 0.380 | 7.044E-01 |
|  | bSpline(visit_num, knots = 1, degree = 1)1 | -29.825 | 1.791 | 532 | -16.649 | 2.085E-50 |
|  | bSpline(visit_num, knots = 1, degree = 1)2 | -19.067 | 2.462 | 532 | -7.746 | 4.831E-14 |
|  | sexFemale | 1.531 | 1.320 | 169 | 1.159 | 2.480E-01 |
|  | race_binaryOther | 3.399 | 1.279 | 169 | 2.659 | 8.600E-03 |
|  | ageyrs | -0.691 | 0.367 | 532 | -1.883 | 6.028E-02 |
|  | site_binary | -2.657 | 1.160 | 169 | -2.291 | 2.317E-02 |
|  | parents_income25000-74999 | 0.277 | 1.304 | 169 | 0.213 | 8.319E-01 |
|  | parents_income75000 or more | -0.955 | 1.502 | 169 | -0.636 | 5.258E-01 |
|  | pf_hx_s_targeted_plasma_tert1.36<-2.34:bSpline(visit_num, knots = 1, degree = 1)1 | -0.840 | 2.442 | 532 | -0.344 | 7.311E-01 |
|  | pf_hx_s_targeted_plasma_tert2.34<:bSpline(visit_num, knots = 1, degree = 1)1 | 1.190 | 2.526 | 532 | 0.471 | 6.376E-01 |
|  | pf_hx_s_targeted_plasma_tert1.36<-2.34:bSpline(visit_num, knots = 1, degree = 1)2 | 2.241 | 2.237 | 532 | 1.002 | 3.169E-01 |
|  | pf_hx_s_targeted_plasma_tert2.34<:bSpline(visit_num, knots = 1, degree = 1)2 | 2.844 | 2.282 | 532 | 1.246 | 2.132E-01 |
| PFHpS | (Intercept) | 9.520 | 6.701 | 532 | 1.421 | 1.560E-01 |
|  | pf_hp_s_targeted_plasma_tert0.146<-0.21 | 0.836 | 1.912 | 169 | 0.437 | 6.625E-01 |
|  | pf_hp_s_targeted_plasma_tert0.21< | 0.971 | 1.927 | 169 | 0.504 | 6.148E-01 |
|  | bSpline(visit_num, knots = 1, degree = 1)1 | -29.432 | 1.799 | 532 | -16.357 | 5.154E-49 |
|  | bSpline(visit_num, knots = 1, degree = 1)2 | -18.721 | 2.466 | 532 | -7.593 | 1.411E-13 |
|  | sexFemale | 1.948 | 1.345 | 169 | 1.448 | 1.494E-01 |
|  | race_binaryOther | 3.683 | 1.279 | 169 | 2.881 | 4.484E-03 |
|  | ageyrs | -0.812 | 0.369 | 532 | -2.202 | 2.807E-02 |
|  | site_binary | -2.523 | 1.152 | 169 | -2.190 | 2.989E-02 |
|  | parents_income25000-74999 | 0.072 | 1.290 | 169 | 0.056 | 9.553E-01 |
|  | parents_income75000 or more | -1.315 | 1.516 | 169 | -0.867 | 3.870E-01 |
|  | pf_hp_s_targeted_plasma_tert0.146<-0.21:bSpline(visit_num, knots = 1, degree = 1)1 | -0.214 | 2.446 | 532 | -0.087 | 9.304E-01 |
|  | pf_hp_s_targeted_plasma_tert0.21<:bSpline(visit_num, knots = 1, degree = 1)1 | -0.410 | 2.480 | 532 | -0.165 | 8.687E-01 |
|  | pf_hp_s_targeted_plasma_tert0.146<-0.21:bSpline(visit_num, knots = 1, degree = 1)2 | -0.572 | 2.260 | 532 | -0.253 | 8.001E-01 |
|  | pf_hp_s_targeted_plasma_tert0.21<:bSpline(visit_num, knots = 1, degree = 1)2 | 6.119 | 2.235 | 532 | 2.738 | 6.398E-03 |
| Sum Sulfonic Acids | (Intercept) | 7.591 | 6.738 | 532 | 1.127 | 2.604E-01 |
|  | sum_sulfonic_tert5.79<-8.29 | 1.119 | 1.907 | 169 | 0.587 | 5.581E-01 |
|  | sum_sulfonic_tert8.29< | 0.489 | 1.909 | 169 | 0.256 | 7.982E-01 |
|  | bSpline(visit_num, knots = 1, degree = 1)1 | -29.470 | 1.800 | 532 | -16.376 | 4.184E-49 |
|  | bSpline(visit_num, knots = 1, degree = 1)2 | -18.238 | 2.457 | 532 | -7.423 | 4.573E-13 |
|  | sexFemale | 1.529 | 1.321 | 169 | 1.157 | 2.488E-01 |
|  | race_binaryOther | 3.121 | 1.277 | 169 | 2.444 | 1.555E-02 |
|  | ageyrs | -0.671 | 0.366 | 532 | -1.833 | 6.741E-02 |
|  | site_binary | -2.645 | 1.167 | 169 | -2.267 | 2.466E-02 |
|  | parents_income25000-74999 | 0.262 | 1.296 | 169 | 0.202 | 8.399E-01 |
|  | parents_income75000 or more | -1.135 | 1.514 | 169 | -0.750 | 4.544E-01 |
|  | sum_sulfonic_tert5.79<-8.29:bSpline(visit_num, knots = 1, degree = 1)1 | -2.591 | 2.438 | 532 | -1.063 | 2.885E-01 |
|  | sum_sulfonic_tert8.29<:bSpline(visit_num, knots = 1, degree = 1)1 | 1.982 | 2.523 | 532 | 0.786 | 4.324E-01 |
|  | sum_sulfonic_tert5.79<-8.29:bSpline(visit_num, knots = 1, degree = 1)2 | -1.257 | 2.250 | 532 | -0.559 | 5.766E-01 |
|  | sum_sulfonic_tert8.29<:bSpline(visit_num, knots = 1, degree = 1)2 | 3.569 | 2.247 | 532 | 1.588 | 1.129E-01 |
| PFOA | (Intercept) | 8.540 | 6.814 | 532 | 1.253 | 2.106E-01 |
|  | pfoa_targeted_plasma_tert1.86<-2.75 | 0.839 | 1.927 | 169 | 0.436 | 6.637E-01 |
|  | pfoa_targeted_plasma_tert2.75< | 1.933 | 1.957 | 169 | 0.988 | 3.246E-01 |
|  | bSpline(visit_num, knots = 1, degree = 1)1 | -29.480 | 1.809 | 532 | -16.298 | 9.814E-49 |
|  | bSpline(visit_num, knots = 1, degree = 1)2 | -15.785 | 2.489 | 532 | -6.343 | 4.818E-10 |
|  | sexFemale | 1.549 | 1.340 | 169 | 1.156 | 2.495E-01 |
|  | race_binaryOther | 3.398 | 1.273 | 169 | 2.670 | 8.319E-03 |
|  | ageyrs | -0.753 | 0.373 | 532 | -2.017 | 4.422E-02 |
|  | site_binary | -2.821 | 1.195 | 169 | -2.361 | 1.936E-02 |
|  | parents_income25000-74999 | 0.319 | 1.320 | 169 | 0.241 | 8.095E-01 |
|  | parents_income75000 or more | -0.981 | 1.539 | 169 | -0.637 | 5.249E-01 |
|  | pfoa_targeted_plasma_tert1.86<-2.75:bSpline(visit_num, knots = 1, degree = 1)1 | -0.746 | 2.489 | 532 | -0.300 | 7.645E-01 |
|  | pfoa_targeted_plasma_tert2.75<:bSpline(visit_num, knots = 1, degree = 1)1 | 0.040 | 2.483 | 532 | 0.016 | 9.871E-01 |
|  | pfoa_targeted_plasma_tert1.86<-2.75:bSpline(visit_num, knots = 1, degree = 1)2 | -1.418 | 2.269 | 532 | -0.625 | 5.322E-01 |
|  | pfoa_targeted_plasma_tert2.75<:bSpline(visit_num, knots = 1, degree = 1)2 | -2.427 | 2.259 | 532 | -1.074 | 2.831E-01 |
| PFNA | (Intercept) | 9.656 | 6.772 | 532 | 1.426 | 1.545E-01 |
|  | pfna_targeted_plasma_tert0.574<-0.796 | 0.171 | 1.914 | 169 | 0.089 | 9.291E-01 |
|  | pfna_targeted_plasma_tert0.796< | -0.357 | 1.961 | 169 | -0.182 | 8.557E-01 |
|  | bSpline(visit_num, knots = 1, degree = 1)1 | -31.057 | 1.795 | 532 | -17.302 | 1.537E-53 |
|  | bSpline(visit_num, knots = 1, degree = 1)2 | -18.563 | 2.477 | 532 | -7.495 | 2.785E-13 |
|  | sexFemale | 1.438 | 1.328 | 169 | 1.083 | 2.803E-01 |
|  | race_binaryOther | 3.295 | 1.269 | 169 | 2.596 | 1.026E-02 |
|  | ageyrs | -0.760 | 0.371 | 532 | -2.050 | 4.085E-02 |
|  | site_binary | -2.419 | 1.196 | 169 | -2.022 | 4.478E-02 |
|  | parents_income25000-74999 | 0.186 | 1.305 | 169 | 0.142 | 8.869E-01 |
|  | parents_income75000 or more | -1.125 | 1.554 | 169 | -0.724 | 4.700E-01 |
|  | pfna_targeted_plasma_tert0.574<-0.796:bSpline(visit_num, knots = 1, degree = 1)1 | 1.151 | 2.455 | 532 | 0.469 | 6.393E-01 |
|  | pfna_targeted_plasma_tert0.796<:bSpline(visit_num, knots = 1, degree = 1)1 | 2.996 | 2.513 | 532 | 1.192 | 2.338E-01 |
|  | pfna_targeted_plasma_tert0.574<-0.796:bSpline(visit_num, knots = 1, degree = 1)2 | 1.872 | 2.264 | 532 | 0.827 | 4.087E-01 |
|  | pfna_targeted_plasma_tert0.796<:bSpline(visit_num, knots = 1, degree = 1)2 | 2.640 | 2.284 | 532 | 1.156 | 2.483E-01 |
| PFDA | (Intercept) | 8.225 | 6.760 | 532 | 1.217 | 2.243E-01 |
|  | pfda_targeted_plasma_tert0.145<0.196 | -0.127 | 1.899 | 169 | -0.067 | 9.468E-01 |
|  | pfda_targeted_plasma_tert0.196< | -1.437 | 1.930 | 169 | -0.745 | 4.574E-01 |
|  | bSpline(visit_num, knots = 1, degree = 1)1 | -30.013 | 1.806 | 532 | -16.616 | 3.013E-50 |
|  | bSpline(visit_num, knots = 1, degree = 1)2 | -16.107 | 2.423 | 532 | -6.647 | 7.439E-11 |
|  | sexFemale | 1.547 | 1.319 | 169 | 1.173 | 2.423E-01 |
|  | race_binaryOther | 3.553 | 1.273 | 169 | 2.790 | 5.871E-03 |
|  | ageyrs | -0.679 | 0.366 | 532 | -1.853 | 6.445E-02 |
|  | site_binary | -2.818 | 1.164 | 169 | -2.422 | 1.650E-02 |
|  | parents_income25000-74999 | 0.993 | 1.323 | 169 | 0.750 | 4.542E-01 |
|  | parents_income75000 or more | -0.253 | 1.537 | 169 | -0.165 | 8.695E-01 |
|  | pfda_targeted_plasma_tert0.145<0.196:bSpline(visit_num, knots = 1, degree = 1)1 | -0.525 | 2.465 | 532 | -0.213 | 8.316E-01 |
|  | pfda_targeted_plasma_tert0.196<:bSpline(visit_num, knots = 1, degree = 1)1 | 1.169 | 2.489 | 532 | 0.470 | 6.387E-01 |
|  | pfda_targeted_plasma_tert0.145<0.196:bSpline(visit_num, knots = 1, degree = 1)2 | -0.002 | 2.272 | 532 | -0.001 | 9.993E-01 |
|  | pfda_targeted_plasma_tert0.196<:bSpline(visit_num, knots = 1, degree = 1)2 | -3.842 | 2.209 | 532 | -1.739 | 8.253E-02 |
| PFHpA | (Intercept) | 8.867 | 6.887 | 532 | 1.288 | 1.985E-01 |
|  | pf_hp_a_targeted_plasma_tert0.0568<-0.145 | -0.005 | 1.902 | 169 | -0.003 | 9.980E-01 |
|  | pf_hp_a_targeted_plasma_tert0.145< | 0.507 | 1.901 | 169 | 0.267 | 7.899E-01 |
|  | bSpline(visit_num, knots = 1, degree = 1)1 | -28.488 | 1.837 | 532 | -15.512 | 4.840E-45 |
|  | bSpline(visit_num, knots = 1, degree = 1)2 | -15.261 | 2.491 | 532 | -6.126 | 1.758E-09 |
|  | sexFemale | 1.387 | 1.326 | 169 | 1.046 | 2.972E-01 |
|  | race_binaryOther | 3.407 | 1.271 | 169 | 2.682 | 8.049E-03 |
|  | ageyrs | -0.739 | 0.370 | 532 | -1.999 | 4.616E-02 |
|  | site_binary | -2.637 | 1.160 | 169 | -2.274 | 2.420E-02 |
|  | parents_income25000-74999 | 0.631 | 1.292 | 169 | 0.488 | 6.260E-01 |
|  | parents_income75000 or more | -0.565 | 1.503 | 169 | -0.376 | 7.076E-01 |
|  | pf_hp_a_targeted_plasma_tert0.0568<-0.145:bSpline(visit_num, knots = 1, degree = 1)1 | -2.726 | 2.483 | 532 | -1.098 | 2.728E-01 |
|  | pf_hp_a_targeted_plasma_tert0.145<:bSpline(visit_num, knots = 1, degree = 1)1 | -0.981 | 2.499 | 532 | -0.393 | 6.948E-01 |
|  | pf_hp_a_targeted_plasma_tert0.0568<-0.145:bSpline(visit_num, knots = 1, degree = 1)2 | -2.112 | 2.279 | 532 | -0.927 | 3.545E-01 |
|  | pf_hp_a_targeted_plasma_tert0.145<:bSpline(visit_num, knots = 1, degree = 1)2 | -3.406 | 2.249 | 532 | -1.515 | 1.304E-01 |
| PFUnDA | (Intercept) | 9.701 | 6.764 | 532 | 1.434 | 1.521E-01 |
|  | pf_un_da_targeted_plasma_tert0.046<-0.0733 | -0.064 | 1.902 | 169 | -0.034 | 9.731E-01 |
|  | pf_un_da_targeted_plasma_tert0.0733< | -1.657 | 1.897 | 169 | -0.873 | 3.838E-01 |
|  | bSpline(visit_num, knots = 1, degree = 1)1 | -31.326 | 1.736 | 532 | -18.048 | 3.709E-57 |
|  | bSpline(visit_num, knots = 1, degree = 1)2 | -18.689 | 2.416 | 532 | -7.736 | 5.183E-14 |
|  | sexFemale | 1.238 | 1.328 | 169 | 0.933 | 3.523E-01 |
|  | race_binaryOther | 3.457 | 1.316 | 169 | 2.627 | 9.414E-03 |
|  | ageyrs | -0.730 | 0.370 | 532 | -1.973 | 4.901E-02 |
|  | site_binary | -2.623 | 1.179 | 169 | -2.225 | 2.742E-02 |
|  | parents_income25000-74999 | 0.326 | 1.297 | 169 | 0.252 | 8.016E-01 |
|  | parents_income75000 or more | -0.728 | 1.505 | 169 | -0.483 | 6.295E-01 |
|  | pf_un_da_targeted_plasma_tert0.046<-0.0733:bSpline(visit_num, knots = 1, degree = 1)1 | 1.791 | 2.479 | 532 | 0.723 | 4.702E-01 |
|  | pf_un_da_targeted_plasma_tert0.0733<:bSpline(visit_num, knots = 1, degree = 1)1 | 3.092 | 2.450 | 532 | 1.262 | 2.074E-01 |
|  | pf_un_da_targeted_plasma_tert0.046<-0.0733:bSpline(visit_num, knots = 1, degree = 1)2 | 1.849 | 2.276 | 532 | 0.812 | 4.169E-01 |
|  | pf_un_da_targeted_plasma_tert0.0733<:bSpline(visit_num, knots = 1, degree = 1)2 | 2.935 | 2.200 | 532 | 1.334 | 1.828E-01 |
| Sum Carboxylic Acids | (Intercept) | 8.035 | 6.784 | 532 | 1.184 | 2.368E-01 |
|  | sum_carboxylic_tert2.87<-4.03 | 0.731 | 1.946 | 169 | 0.375 | 7.078E-01 |
|  | sum_carboxylic_tert4.03< | 1.392 | 1.952 | 169 | 0.713 | 4.768E-01 |
|  | bSpline(visit_num, knots = 1, degree = 1)1 | -29.053 | 1.820 | 532 | -15.959 | 3.893E-47 |
|  | bSpline(visit_num, knots = 1, degree = 1)2 | -15.803 | 2.479 | 532 | -6.376 | 3.950E-10 |
|  | sexFemale | 1.495 | 1.346 | 169 | 1.110 | 2.685E-01 |
|  | race_binaryOther | 3.319 | 1.271 | 169 | 2.612 | 9.814E-03 |
|  | ageyrs | -0.717 | 0.372 | 532 | -1.926 | 5.460E-02 |
|  | site_binary | -2.598 | 1.187 | 169 | -2.189 | 2.997E-02 |
|  | parents_income25000-74999 | 0.493 | 1.329 | 169 | 0.371 | 7.111E-01 |
|  | parents_income75000 or more | -0.980 | 1.553 | 169 | -0.631 | 5.289E-01 |
|  | sum_carboxylic_tert2.87<-4.03:bSpline(visit_num, knots = 1, degree = 1)1 | -2.144 | 2.493 | 532 | -0.860 | 3.902E-01 |
|  | sum_carboxylic_tert4.03<:bSpline(visit_num, knots = 1, degree = 1)1 | -0.005 | 2.488 | 532 | -0.002 | 9.984E-01 |
|  | sum_carboxylic_tert2.87<-4.03:bSpline(visit_num, knots = 1, degree = 1)2 | -2.133 | 2.266 | 532 | -0.941 | 3.469E-01 |
|  | sum_carboxylic_tert4.03<:bSpline(visit_num, knots = 1, degree = 1)2 | -2.244 | 2.251 | 532 | -0.997 | 3.194E-01 |

Table S5. Linear mixed model outputs for each PFAS congener (in tertiles) on waist circumference.

|  | Term | Value | Std.Error | DF | t-value | p-value |
| --- | --- | --- | --- | --- | --- | --- |
| PFOS | (Intercept) | 146.154 | 14.422 | 485 | 10.134 | 5.086E-22 |
|  | total_pfos_targeted_plasma_tert3.92-5.66 | 3.273 | 3.339 | 169 | 0.980 | 3.283E-01 |
|  | total_pfos_targeted_plasma_tert5.66< | 1.317 | 3.386 | 169 | 0.389 | 6.977E-01 |
|  | bSpline(visit_num, knots = 1, degree = 1)1 | -28.221 | 2.210 | 485 | -12.770 | 2.143E-32 |
|  | bSpline(visit_num, knots = 1, degree = 1)2 | -27.011 | 4.499 | 485 | -6.004 | 3.773E-09 |
|  | sexFemale | -8.166 | 2.866 | 169 | -2.849 | 4.927E-03 |
|  | race_binaryOther | 7.858 | 2.775 | 169 | 2.832 | 5.187E-03 |
|  | ageyrs | 0.054 | 0.798 | 485 | 0.068 | 9.456E-01 |
|  | site_binary | -5.872 | 2.529 | 169 | -2.322 | 2.141E-02 |
|  | parents_income25000-74999 | -5.597 | 2.824 | 169 | -1.982 | 4.913E-02 |
|  | parents_income75000 or more | -11.419 | 3.319 | 169 | -3.441 | 7.309E-04 |
|  | total_pfos_targeted_plasma_tert3.92-5.66:bSpline(visit_num, knots = 1, degree = 1)1 | -4.167 | 2.869 | 485 | -1.453 | 1.470E-01 |
|  | total_pfos_targeted_plasma_tert5.66<:bSpline(visit_num, knots = 1, degree = 1)1 | -2.715 | 2.942 | 485 | -0.923 | 3.565E-01 |
|  | total_pfos_targeted_plasma_tert3.92-5.66:bSpline(visit_num, knots = 1, degree = 1)2 | -0.099 | 2.826 | 485 | -0.035 | 9.722E-01 |
|  | total_pfos_targeted_plasma_tert5.66<:bSpline(visit_num, knots = 1, degree = 1)2 | 2.726 | 2.783 | 485 | 0.979 | 3.279E-01 |
| PFHxS | (Intercept) | 146.452 | 14.385 | 485 | 10.181 | 3.422E-22 |
|  | pf_hx_s_targeted_plasma_tert1.36<-2.34 | -1.253 | 3.333 | 169 | -0.376 | 7.075E-01 |
|  | pf_hx_s_targeted_plasma_tert2.34< | 1.617 | 3.337 | 169 | 0.484 | 6.287E-01 |
|  | bSpline(visit_num, knots = 1, degree = 1)1 | -30.009 | 2.214 | 485 | -13.553 | 1.029E-35 |
|  | bSpline(visit_num, knots = 1, degree = 1)2 | -28.811 | 4.484 | 485 | -6.425 | 3.147E-10 |
|  | sexFemale | -7.988 | 2.848 | 169 | -2.805 | 5.625E-03 |
|  | race_binaryOther | 7.497 | 2.770 | 169 | 2.706 | 7.506E-03 |
|  | ageyrs | 0.115 | 0.781 | 485 | 0.147 | 8.831E-01 |
|  | site_binary | -6.010 | 2.507 | 169 | -2.398 | 1.759E-02 |
|  | parents_income25000-74999 | -5.207 | 2.811 | 169 | -1.852 | 6.578E-02 |
|  | parents_income75000 or more | -11.631 | 3.253 | 169 | -3.575 | 4.566E-04 |
|  | pf_hx_s_targeted_plasma_tert1.36<-2.34:bSpline(visit_num, knots = 1, degree = 1)1 | -1.725 | 2.848 | 485 | -0.606 | 5.449E-01 |
|  | pf_hx_s_targeted_plasma_tert2.34<:bSpline(visit_num, knots = 1, degree = 1)1 | 0.223 | 2.983 | 485 | 0.075 | 9.404E-01 |
|  | pf_hx_s_targeted_plasma_tert1.36<-2.34:bSpline(visit_num, knots = 1, degree = 1)2 | 2.358 | 2.801 | 485 | 0.842 | 4.003E-01 |
|  | pf_hx_s_targeted_plasma_tert2.34<:bSpline(visit_num, knots = 1, degree = 1)2 | 4.625 | 2.852 | 485 | 1.622 | 1.055E-01 |
| PFHpS | (Intercept) | 147.903 | 14.196 | 485 | 10.418 | 4.515E-23 |
|  | pf_hp_s_targeted_plasma_tert0.146<-0.21 | -0.120 | 3.367 | 169 | -0.036 | 9.716E-01 |
|  | pf_hp_s_targeted_plasma_tert0.21< | 4.075 | 3.432 | 169 | 1.188 | 2.367E-01 |
|  | bSpline(visit_num, knots = 1, degree = 1)1 | -28.663 | 2.259 | 485 | -12.689 | 4.665E-32 |
|  | bSpline(visit_num, knots = 1, degree = 1)2 | -27.518 | 4.502 | 485 | -6.112 | 2.022E-09 |
|  | sexFemale | -6.974 | 2.908 | 169 | -2.399 | 1.755E-02 |
|  | race_binaryOther | 8.019 | 2.774 | 169 | 2.891 | 4.343E-03 |
|  | ageyrs | -0.084 | 0.786 | 485 | -0.107 | 9.152E-01 |
|  | site_binary | -5.717 | 2.498 | 169 | -2.289 | 2.331E-02 |
|  | parents_income25000-74999 | -5.711 | 2.789 | 169 | -2.048 | 4.212E-02 |
|  | parents_income75000 or more | -12.211 | 3.293 | 169 | -3.709 | 2.824E-04 |
|  | pf_hp_s_targeted_plasma_tert0.146<-0.21:bSpline(visit_num, knots = 1, degree = 1)1 | -1.827 | 2.873 | 485 | -0.636 | 5.252E-01 |
|  | pf_hp_s_targeted_plasma_tert0.21<:bSpline(visit_num, knots = 1, degree = 1)1 | -3.041 | 2.927 | 485 | -1.039 | 2.994E-01 |
|  | pf_hp_s_targeted_plasma_tert0.146<-0.21:bSpline(visit_num, knots = 1, degree = 1)2 | -0.806 | 2.805 | 485 | -0.287 | 7.740E-01 |
|  | pf_hp_s_targeted_plasma_tert0.21<:bSpline(visit_num, knots = 1, degree = 1)2 | 6.783 | 2.812 | 485 | 2.412 | 1.624E-02 |
| Sum Sulfonic Acids | (Intercept) | 144.525 | 14.329 | 485 | 10.086 | 7.592E-22 |
|  | sum_sulfonic_tert5.79<-8.29 | 2.724 | 3.378 | 169 | 0.807 | 4.211E-01 |
|  | sum_sulfonic_tert8.29< | 3.074 | 3.365 | 169 | 0.914 | 3.622E-01 |
|  | bSpline(visit_num, knots = 1, degree = 1)1 | -28.011 | 2.222 | 485 | -12.605 | 1.037E-31 |
|  | bSpline(visit_num, knots = 1, degree = 1)2 | -26.807 | 4.486 | 485 | -5.976 | 4.431E-09 |
|  | sexFemale | -7.998 | 2.867 | 169 | -2.790 | 5.882E-03 |
|  | race_binaryOther | 7.338 | 2.790 | 169 | 2.630 | 9.321E-03 |
|  | ageyrs | 0.136 | 0.784 | 485 | 0.174 | 8.622E-01 |
|  | site_binary | -5.920 | 2.538 | 169 | -2.332 | 2.088E-02 |
|  | parents_income25000-74999 | -5.646 | 2.813 | 169 | -2.007 | 4.637E-02 |
|  | parents_income75000 or more | -12.028 | 3.301 | 169 | -3.643 | 3.578E-04 |
|  | sum_sulfonic_tert5.79<-8.29:bSpline(visit_num, knots = 1, degree = 1)1 | -6.207 | 2.832 | 485 | -2.191 | 2.891E-02 |
|  | sum_sulfonic_tert8.29<:bSpline(visit_num, knots = 1, degree = 1)1 | -0.978 | 2.965 | 485 | -0.330 | 7.416E-01 |
|  | sum_sulfonic_tert5.79<-8.29:bSpline(visit_num, knots = 1, degree = 1)2 | -3.318 | 2.807 | 485 | -1.182 | 2.379E-01 |
|  | sum_sulfonic_tert8.29<:bSpline(visit_num, knots = 1, degree = 1)2 | 4.022 | 2.787 | 485 | 1.443 | 1.496E-01 |
| PFOA | (Intercept) | 145.924 | 14.438 | 485 | 10.107 | 6.398E-22 |
|  | pfoa_targeted_plasma_tert1.86<-2.75 | 2.104 | 3.401 | 169 | 0.619 | 5.370E-01 |
|  | pfoa_targeted_plasma_tert2.75< | 2.700 | 3.486 | 169 | 0.775 | 4.397E-01 |
|  | bSpline(visit_num, knots = 1, degree = 1)1 | -28.486 | 2.244 | 485 | -12.692 | 4.521E-32 |
|  | bSpline(visit_num, knots = 1, degree = 1)2 | -24.275 | 4.542 | 485 | -5.345 | 1.394E-07 |
|  | sexFemale | -8.054 | 2.901 | 169 | -2.776 | 6.122E-03 |
|  | race_binaryOther | 7.745 | 2.765 | 169 | 2.801 | 5.694E-03 |
|  | ageyrs | 0.060 | 0.796 | 485 | 0.076 | 9.396E-01 |
|  | site_binary | -5.987 | 2.595 | 169 | -2.308 | 2.224E-02 |
|  | parents_income25000-74999 | -5.477 | 2.856 | 169 | -1.918 | 5.682E-02 |
|  | parents_income75000 or more | -11.463 | 3.346 | 169 | -3.426 | 7.688E-04 |
|  | pfoa_targeted_plasma_tert1.86<-2.75:bSpline(visit_num, knots = 1, degree = 1)1 | -3.397 | 2.914 | 485 | -1.166 | 2.443E-01 |
|  | pfoa_targeted_plasma_tert2.75<:bSpline(visit_num, knots = 1, degree = 1)1 | -2.727 | 2.914 | 485 | -0.936 | 3.499E-01 |
|  | pfoa_targeted_plasma_tert1.86<-2.75:bSpline(visit_num, knots = 1, degree = 1)2 | -2.578 | 2.834 | 485 | -0.910 | 3.634E-01 |
|  | pfoa_targeted_plasma_tert2.75<:bSpline(visit_num, knots = 1, degree = 1)2 | -3.347 | 2.807 | 485 | -1.192 | 2.337E-01 |
| PFNA | (Intercept) | 148.673 | 14.150 | 485 | 10.507 | 2.099E-23 |
|  | pfna_targeted_plasma_tert0.574<-0.796 | -8.336 | 3.342 | 169 | -2.494 | 1.359E-02 |
|  | pfna_targeted_plasma_tert0.796< | -3.514 | 3.444 | 169 | -1.020 | 3.091E-01 |
|  | bSpline(visit_num, knots = 1, degree = 1)1 | -31.373 | 2.237 | 485 | -14.024 | 9.518E-38 |
|  | bSpline(visit_num, knots = 1, degree = 1)2 | -29.802 | 4.461 | 485 | -6.681 | 6.520E-11 |
|  | sexFemale | -8.856 | 2.833 | 169 | -3.126 | 2.086E-03 |
|  | race_binaryOther | 7.686 | 2.717 | 169 | 2.828 | 5.243E-03 |
|  | ageyrs | 0.195 | 0.779 | 485 | 0.250 | 8.029E-01 |
|  | site_binary | -5.307 | 2.563 | 169 | -2.071 | 3.987E-02 |
|  | parents_income25000-74999 | -4.409 | 2.780 | 169 | -1.586 | 1.146E-01 |
|  | parents_income75000 or more | -9.601 | 3.327 | 169 | -2.886 | 4.416E-03 |
|  | pfna_targeted_plasma_tert0.574<-0.796:bSpline(visit_num, knots = 1, degree = 1)1 | 1.161 | 2.890 | 485 | 0.402 | 6.881E-01 |
|  | pfna_targeted_plasma_tert0.796<:bSpline(visit_num, knots = 1, degree = 1)1 | 0.881 | 2.950 | 485 | 0.299 | 7.654E-01 |
|  | pfna_targeted_plasma_tert0.574<-0.796:bSpline(visit_num, knots = 1, degree = 1)2 | 4.246 | 2.867 | 485 | 1.481 | 1.393E-01 |
|  | pfna_targeted_plasma_tert0.796<:bSpline(visit_num, knots = 1, degree = 1)2 | 4.282 | 2.805 | 485 | 1.526 | 1.276E-01 |
| PFDA | (Intercept) | 147.407 | 14.306 | 485 | 10.304 | 1.199E-22 |
|  | pfda_targeted_plasma_tert0.145<0.196 | -3.189 | 3.332 | 169 | -0.957 | 3.400E-01 |
|  | pfda_targeted_plasma_tert0.196< | -2.992 | 3.419 | 169 | -0.875 | 3.828E-01 |
|  | bSpline(visit_num, knots = 1, degree = 1)1 | -29.573 | 2.241 | 485 | -13.199 | 3.345E-34 |
|  | bSpline(visit_num, knots = 1, degree = 1)2 | -25.817 | 4.449 | 485 | -5.803 | 1.178E-08 |
|  | sexFemale | -7.894 | 2.845 | 169 | -2.774 | 6.157E-03 |
|  | race_binaryOther | 8.150 | 2.757 | 169 | 2.956 | 3.560E-03 |
|  | ageyrs | 0.124 | 0.780 | 485 | 0.159 | 8.734E-01 |
|  | site_binary | -6.172 | 2.518 | 169 | -2.451 | 1.528E-02 |
|  | parents_income25000-74999 | -4.072 | 2.858 | 169 | -1.425 | 1.561E-01 |
|  | parents_income75000 or more | -10.025 | 3.335 | 169 | -3.006 | 3.049E-03 |
|  | pfda_targeted_plasma_tert0.145<0.196:bSpline(visit_num, knots = 1, degree = 1)1 | -0.330 | 2.897 | 485 | -0.114 | 9.093E-01 |
|  | pfda_targeted_plasma_tert0.196<:bSpline(visit_num, knots = 1, degree = 1)1 | -2.775 | 2.922 | 485 | -0.949 | 3.429E-01 |
|  | pfda_targeted_plasma_tert0.145<0.196:bSpline(visit_num, knots = 1, degree = 1)2 | 3.117 | 2.923 | 485 | 1.067 | 2.867E-01 |
|  | pfda_targeted_plasma_tert0.196<:bSpline(visit_num, knots = 1, degree = 1)2 | -4.294 | 2.717 | 485 | -1.581 | 1.146E-01 |
| PFHpA | (Intercept) | 141.082 | 14.572 | 485 | 9.682 | 2.186E-20 |
|  | pf_hp_a_targeted_plasma_tert0.0568<-0.145 | 1.677 | 3.322 | 169 | 0.505 | 6.143E-01 |
|  | pf_hp_a_targeted_plasma_tert0.145< | 8.279 | 3.328 | 169 | 2.488 | 1.383E-02 |
|  | bSpline(visit_num, knots = 1, degree = 1)1 | -27.592 | 2.270 | 485 | -12.156 | 7.301E-30 |
|  | bSpline(visit_num, knots = 1, degree = 1)2 | -23.930 | 4.561 | 485 | -5.247 | 2.313E-07 |
|  | sexFemale | -7.592 | 2.864 | 169 | -2.651 | 8.784E-03 |
|  | race_binaryOther | 7.967 | 2.752 | 169 | 2.895 | 4.296E-03 |
|  | ageyrs | 0.219 | 0.786 | 485 | 0.278 | 7.811E-01 |
|  | site_binary | -5.791 | 2.508 | 169 | -2.310 | 2.212E-02 |
|  | parents_income25000-74999 | -5.521 | 2.789 | 169 | -1.979 | 4.942E-02 |
|  | parents_income75000 or more | -11.853 | 3.261 | 169 | -3.634 | 3.697E-04 |
|  | pf_hp_a_targeted_plasma_tert0.0568<-0.145:bSpline(visit_num, knots = 1, degree = 1)1 | -3.378 | 2.890 | 485 | -1.169 | 2.430E-01 |
|  | pf_hp_a_targeted_plasma_tert0.145<:bSpline(visit_num, knots = 1, degree = 1)1 | -5.801 | 2.909 | 485 | -1.994 | 4.673E-02 |
|  | pf_hp_a_targeted_plasma_tert0.0568<-0.145:bSpline(visit_num, knots = 1, degree = 1)2 | -1.026 | 2.827 | 485 | -0.363 | 7.169E-01 |
|  | pf_hp_a_targeted_plasma_tert0.145<:bSpline(visit_num, knots = 1, degree = 1)2 | -8.144 | 2.823 | 485 | -2.885 | 4.089E-03 |
| PFUnDA | (Intercept) | 148.369 | 14.276 | 485 | 10.393 | 5.597E-23 |
|  | pf_un_da_targeted_plasma_tert0.046<-0.0733 | -6.096 | 3.319 | 169 | -1.837 | 6.798E-02 |
|  | pf_un_da_targeted_plasma_tert0.0733< | -2.825 | 3.344 | 169 | -0.845 | 3.994E-01 |
|  | bSpline(visit_num, knots = 1, degree = 1)1 | -32.088 | 2.138 | 485 | -15.009 | 4.233E-42 |
|  | bSpline(visit_num, knots = 1, degree = 1)2 | -28.972 | 4.458 | 485 | -6.498 | 2.018E-10 |
|  | sexFemale | -7.857 | 2.858 | 169 | -2.749 | 6.628E-03 |
|  | race_binaryOther | 7.516 | 2.835 | 169 | 2.651 | 8.777E-03 |
|  | ageyrs | 0.151 | 0.785 | 485 | 0.192 | 8.481E-01 |
|  | site_binary | -5.810 | 2.543 | 169 | -2.285 | 2.358E-02 |
|  | parents_income25000-74999 | -5.034 | 2.789 | 169 | -1.805 | 7.291E-02 |
|  | parents_income75000 or more | -11.206 | 3.253 | 169 | -3.444 | 7.216E-04 |
|  | pf_un_da_targeted_plasma_tert0.046<-0.0733:bSpline(visit_num, knots = 1, degree = 1)1 | 3.055 | 2.906 | 485 | 1.051 | 2.938E-01 |
|  | pf_un_da_targeted_plasma_tert0.0733<:bSpline(visit_num, knots = 1, degree = 1)1 | 1.591 | 2.870 | 485 | 0.555 | 5.795E-01 |
|  | pf_un_da_targeted_plasma_tert0.046<-0.0733:bSpline(visit_num, knots = 1, degree = 1)2 | 5.454 | 2.920 | 485 | 1.868 | 6.238E-02 |
|  | pf_un_da_targeted_plasma_tert0.0733<:bSpline(visit_num, knots = 1, degree = 1)2 | 2.226 | 2.703 | 485 | 0.824 | 4.106E-01 |
| Sum Carboxylic Acids | (Intercept) | 144.983 | 14.359 | 485 | 10.097 | 6.946E-22 |
|  | sum_carboxylic_tert2.87<-4.03 | 1.067 | 3.446 | 169 | 0.310 | 7.572E-01 |
|  | sum_carboxylic_tert4.03< | 2.190 | 3.469 | 169 | 0.631 | 5.287E-01 |
|  | bSpline(visit_num, knots = 1, degree = 1)1 | -27.630 | 2.250 | 485 | -12.278 | 2.321E-30 |
|  | bSpline(visit_num, knots = 1, degree = 1)2 | -23.739 | 4.504 | 485 | -5.271 | 2.044E-07 |
|  | sexFemale | -8.213 | 2.911 | 169 | -2.822 | 5.353E-03 |
|  | race_binaryOther | 7.700 | 2.759 | 169 | 2.791 | 5.867E-03 |
|  | ageyrs | 0.131 | 0.793 | 485 | 0.165 | 8.687E-01 |
|  | site_binary | -5.631 | 2.573 | 169 | -2.188 | 3.001E-02 |
|  | parents_income25000-74999 | -5.006 | 2.871 | 169 | -1.744 | 8.305E-02 |
|  | parents_income75000 or more | -11.307 | 3.371 | 169 | -3.354 | 9.822E-04 |
|  | sum_carboxylic_tert2.87<-4.03:bSpline(visit_num, knots = 1, degree = 1)1 | -5.171 | 2.917 | 485 | -1.773 | 7.692E-02 |
|  | sum_carboxylic_tert4.03<:bSpline(visit_num, knots = 1, degree = 1)1 | -3.755 | 2.911 | 485 | -1.290 | 1.978E-01 |
|  | sum_carboxylic_tert2.87<-4.03:bSpline(visit_num, knots = 1, degree = 1)2 | -4.671 | 2.828 | 485 | -1.652 | 9.925E-02 |
|  | sum_carboxylic_tert4.03<:bSpline(visit_num, knots = 1, degree = 1)2 | -4.180 | 2.780 | 485 | -1.504 | 1.333E-01 |

Table S6. Linear mixed model outputs for each log_2_-PFAS congener on BMI.

|  | Term | Value | Std.Error | DF | t-value | p-value |
| --- | --- | --- | --- | --- | --- | --- |
| PFOS | (Intercept) | 59.948 | 7.546 | 528 | 7.944 | 1.186E-14 |
|  | total_pfos_targeted_plasma_log2 | 0.914 | 0.986 | 170 | 0.927 | 3.554E-01 |
|  | bSpline(visit_num, knots = 1, degree = 1)1 | -15.612 | 1.837 | 528 | -8.500 | 1.953E-16 |
|  | bSpline(visit_num, knots = 1, degree = 1)2 | -12.179 | 2.644 | 528 | -4.607 | 5.127E-06 |
|  | race_binaryOther | 5.179 | 1.433 | 170 | 3.615 | 3.956E-04 |
|  | sexFemale | -0.660 | 1.507 | 170 | -0.438 | 6.620E-01 |
|  | site_binary | -3.819 | 1.310 | 170 | -2.916 | 4.021E-03 |
|  | ageyrs | -0.395 | 0.406 | 528 | -0.975 | 3.302E-01 |
|  | parents_income25000-74999 | -3.934 | 1.471 | 170 | -2.673 | 8.239E-03 |
|  | parents_income75000 or more | -6.717 | 1.743 | 170 | -3.854 | 1.648E-04 |
|  | total_pfos_targeted_plasma_log2:bSpline(visit_num, knots = 1, degree = 1)1 | 0.053 | 0.770 | 528 | 0.069 | 9.451E-01 |
|  | total_pfos_targeted_plasma_log2:bSpline(visit_num, knots = 1, degree = 1)2 | 1.384 | 0.704 | 528 | 1.967 | 4.973E-02 |
| PFHxS | (Intercept) | 60.314 | 7.437 | 528 | 8.110 | 3.573E-15 |
|  | pf_hx_s_targeted_plasma_log2 | 0.256 | 0.679 | 170 | 0.377 | 7.066E-01 |
|  | bSpline(visit_num, knots = 1, degree = 1)1 | -15.932 | 0.875 | 528 | -18.203 | 7.972E-58 |
|  | bSpline(visit_num, knots = 1, degree = 1)2 | -10.515 | 2.200 | 528 | -4.780 | 2.275E-06 |
|  | race_binaryOther | 5.170 | 1.440 | 170 | 3.591 | 4.303E-04 |
|  | sexFemale | -0.818 | 1.503 | 170 | -0.544 | 5.871E-01 |
|  | site_binary | -3.907 | 1.316 | 170 | -2.968 | 3.428E-03 |
|  | ageyrs | -0.313 | 0.406 | 528 | -0.771 | 4.408E-01 |
|  | parents_income25000-74999 | -3.721 | 1.461 | 170 | -2.546 | 1.178E-02 |
|  | parents_income75000 or more | -6.372 | 1.716 | 170 | -3.713 | 2.779E-04 |
|  | pf_hx_s_targeted_plasma_log2:bSpline(visit_num, knots = 1, degree = 1)1 | 0.373 | 0.538 | 528 | 0.693 | 4.887E-01 |
|  | pf_hx_s_targeted_plasma_log2:bSpline(visit_num, knots = 1, degree = 1)2 | 1.037 | 0.492 | 528 | 2.107 | 3.557E-02 |
| PFHpS | (Intercept) | 67.336 | 7.902 | 528 | 8.521 | 1.661E-16 |
|  | pf_hp_s_targeted_plasma_log2 | 1.742 | 0.974 | 170 | 1.788 | 7.549E-02 |
|  | bSpline(visit_num, knots = 1, degree = 1)1 | -16.279 | 2.043 | 528 | -7.968 | 1.001E-14 |
|  | bSpline(visit_num, knots = 1, degree = 1)2 | -4.635 | 2.756 | 528 | -1.682 | 9.318E-02 |
|  | race_binaryOther | 5.808 | 1.451 | 170 | 4.003 | 9.338E-05 |
|  | sexFemale | -0.224 | 1.511 | 170 | -0.148 | 8.823E-01 |
|  | site_binary | -3.722 | 1.297 | 170 | -2.869 | 4.636E-03 |
|  | ageyrs | -0.477 | 0.403 | 528 | -1.183 | 2.374E-01 |
|  | parents_income25000-74999 | -4.127 | 1.454 | 170 | -2.838 | 5.098E-03 |
|  | parents_income75000 or more | -7.080 | 1.728 | 170 | -4.097 | 6.465E-05 |
|  | pf_hp_s_targeted_plasma_log2:bSpline(visit_num, knots = 1, degree = 1)1 | -0.352 | 0.757 | 528 | -0.465 | 6.421E-01 |
|  | pf_hp_s_targeted_plasma_log2:bSpline(visit_num, knots = 1, degree = 1)2 | 1.631 | 0.683 | 528 | 2.386 | 1.737E-02 |
| Sum Sulfonic Acids | (Intercept) | 59.103 | 7.764 | 528 | 7.612 | 1.251E-13 |
|  | sum_sulfonic_log2 | 0.786 | 0.948 | 170 | 0.829 | 4.083E-01 |
|  | bSpline(visit_num, knots = 1, degree = 1)1 | -16.580 | 2.189 | 528 | -7.576 | 1.613E-13 |
|  | bSpline(visit_num, knots = 1, degree = 1)2 | -13.707 | 2.861 | 528 | -4.790 | 2.165E-06 |
|  | race_binaryOther | 5.195 | 1.433 | 170 | 3.626 | 3.796E-04 |
|  | sexFemale | -0.644 | 1.505 | 170 | -0.428 | 6.694E-01 |
|  | site_binary | -3.927 | 1.311 | 170 | -2.995 | 3.151E-03 |
|  | ageyrs | -0.354 | 0.404 | 528 | -0.876 | 3.816E-01 |
|  | parents_income25000-74999 | -3.958 | 1.470 | 170 | -2.693 | 7.790E-03 |
|  | parents_income75000 or more | -6.759 | 1.741 | 170 | -3.882 | 1.481E-04 |
|  | sum_sulfonic_log2:bSpline(visit_num, knots = 1, degree = 1)1 | 0.372 | 0.742 | 528 | 0.502 | 6.161E-01 |
|  | sum_sulfonic_log2:bSpline(visit_num, knots = 1, degree = 1)2 | 1.559 | 0.677 | 528 | 2.301 | 2.176E-02 |
| PFOA | (Intercept) | 60.576 | 7.422 | 528 | 8.161 | 2.445E-15 |
|  | pfoa_targeted_plasma_log2 | 0.158 | 0.916 | 170 | 0.173 | 8.631E-01 |
|  | bSpline(visit_num, knots = 1, degree = 1)1 | -15.068 | 1.122 | 528 | -13.430 | 1.387E-35 |
|  | bSpline(visit_num, knots = 1, degree = 1)2 | -8.390 | 2.308 | 528 | -3.636 | 3.040E-04 |
|  | race_binaryOther | 5.015 | 1.442 | 170 | 3.478 | 6.424E-04 |
|  | sexFemale | -1.092 | 1.508 | 170 | -0.724 | 4.699E-01 |
|  | site_binary | -3.706 | 1.345 | 170 | -2.755 | 6.502E-03 |
|  | ageyrs | -0.327 | 0.409 | 528 | -0.799 | 4.247E-01 |
|  | parents_income25000-74999 | -3.458 | 1.460 | 170 | -2.369 | 1.897E-02 |
|  | parents_income75000 or more | -5.980 | 1.723 | 170 | -3.470 | 6.595E-04 |
|  | pfoa_targeted_plasma_log2:bSpline(visit_num, knots = 1, degree = 1)1 | -0.440 | 0.730 | 528 | -0.602 | 5.472E-01 |
|  | pfoa_targeted_plasma_log2:bSpline(visit_num, knots = 1, degree = 1)2 | -0.929 | 0.684 | 528 | -1.359 | 1.749E-01 |
| PFNA | (Intercept) | 61.066 | 7.488 | 528 | 8.156 | 2.547E-15 |
|  | pfna_targeted_plasma_log2 | -0.317 | 1.098 | 170 | -0.288 | 7.734E-01 |
|  | bSpline(visit_num, knots = 1, degree = 1)1 | -14.911 | 0.882 | 528 | -16.910 | 1.389E-51 |
|  | bSpline(visit_num, knots = 1, degree = 1)2 | -8.971 | 2.203 | 528 | -4.072 | 5.373E-05 |
|  | race_binaryOther | 5.043 | 1.440 | 170 | 3.501 | 5.913E-04 |
|  | sexFemale | -0.990 | 1.505 | 170 | -0.658 | 5.116E-01 |
|  | site_binary | -3.737 | 1.340 | 170 | -2.787 | 5.917E-03 |
|  | ageyrs | -0.354 | 0.408 | 528 | -0.867 | 3.865E-01 |
|  | parents_income25000-74999 | -3.565 | 1.465 | 170 | -2.434 | 1.599E-02 |
|  | parents_income75000 or more | -6.193 | 1.752 | 170 | -3.534 | 5.266E-04 |
|  | pfna_targeted_plasma_log2:bSpline(visit_num, knots = 1, degree = 1)1 | 1.150 | 0.879 | 528 | 1.309 | 1.911E-01 |
|  | pfna_targeted_plasma_log2:bSpline(visit_num, knots = 1, degree = 1)2 | 0.694 | 0.786 | 528 | 0.883 | 3.774E-01 |
| PFDA | (Intercept) | 58.015 | 8.056 | 528 | 7.202 | 2.061E-12 |
|  | pfda_targeted_plasma_log2 | -1.051 | 1.094 | 170 | -0.961 | 3.378E-01 |
|  | bSpline(visit_num, knots = 1, degree = 1)1 | -13.538 | 2.317 | 528 | -5.843 | 8.977E-09 |
|  | bSpline(visit_num, knots = 1, degree = 1)2 | -10.878 | 2.908 | 528 | -3.741 | 2.037E-04 |
|  | race_binaryOther | 5.150 | 1.442 | 170 | 3.571 | 4.631E-04 |
|  | sexFemale | -1.088 | 1.497 | 170 | -0.727 | 4.684E-01 |
|  | site_binary | -3.964 | 1.326 | 170 | -2.991 | 3.197E-03 |
|  | ageyrs | -0.326 | 0.406 | 528 | -0.803 | 4.221E-01 |
|  | parents_income25000-74999 | -3.272 | 1.471 | 170 | -2.224 | 2.747E-02 |
|  | parents_income75000 or more | -5.754 | 1.732 | 170 | -3.322 | 1.094E-03 |
|  | pfda_targeted_plasma_log2:bSpline(visit_num, knots = 1, degree = 1)1 | 0.796 | 0.856 | 528 | 0.930 | 3.526E-01 |
|  | pfda_targeted_plasma_log2:bSpline(visit_num, knots = 1, degree = 1)2 | -0.548 | 0.770 | 528 | -0.711 | 4.771E-01 |
| PFHpA | (Intercept) | 62.091 | 7.558 | 528 | 8.215 | 1.648E-15 |
|  | pf_hp_a_targeted_plasma_log2 | 0.340 | 0.528 | 170 | 0.643 | 5.208E-01 |
|  | bSpline(visit_num, knots = 1, degree = 1)1 | -17.076 | 1.673 | 528 | -10.207 | 1.906E-22 |
|  | bSpline(visit_num, knots = 1, degree = 1)2 | -12.089 | 2.551 | 528 | -4.739 | 2.762E-06 |
|  | race_binaryOther | 5.040 | 1.444 | 170 | 3.490 | 6.150E-04 |
|  | sexFemale | -1.043 | 1.511 | 170 | -0.690 | 4.911E-01 |
|  | site_binary | -3.783 | 1.319 | 170 | -2.869 | 4.646E-03 |
|  | ageyrs | -0.335 | 0.407 | 528 | -0.822 | 4.116E-01 |
|  | parents_income25000-74999 | -3.464 | 1.473 | 170 | -2.352 | 1.982E-02 |
|  | parents_income75000 or more | -6.039 | 1.731 | 170 | -3.488 | 6.196E-04 |
|  | pf_hp_a_targeted_plasma_log2:bSpline(visit_num, knots = 1, degree = 1)1 | -0.429 | 0.431 | 528 | -0.994 | 3.205E-01 |
|  | pf_hp_a_targeted_plasma_log2:bSpline(visit_num, knots = 1, degree = 1)2 | -0.760 | 0.384 | 528 | -1.981 | 4.813E-02 |
| PFUnDA | (Intercept) | 58.645 | 8.577 | 528 | 6.837 | 2.240E-11 |
|  | pf_un_da_targeted_plasma_log2 | -0.648 | 0.830 | 170 | -0.780 | 4.362E-01 |
|  | bSpline(visit_num, knots = 1, degree = 1)1 | -10.974 | 2.698 | 528 | -4.068 | 5.463E-05 |
|  | bSpline(visit_num, knots = 1, degree = 1)2 | -5.020 | 3.202 | 528 | -1.568 | 1.175E-01 |
|  | race_binaryOther | 5.042 | 1.484 | 170 | 3.398 | 8.448E-04 |
|  | sexFemale | -1.051 | 1.502 | 170 | -0.700 | 4.848E-01 |
|  | site_binary | -3.793 | 1.336 | 170 | -2.840 | 5.065E-03 |
|  | ageyrs | -0.357 | 0.409 | 528 | -0.873 | 3.831E-01 |
|  | parents_income25000-74999 | -3.535 | 1.472 | 170 | -2.401 | 1.745E-02 |
|  | parents_income75000 or more | -6.106 | 1.721 | 170 | -3.548 | 5.017E-04 |
|  | pf_un_da_targeted_plasma_log2:bSpline(visit_num, knots = 1, degree = 1)1 | 1.102 | 0.623 | 528 | 1.768 | 7.756E-02 |
|  | pf_un_da_targeted_plasma_log2:bSpline(visit_num, knots = 1, degree = 1)2 | 1.036 | 0.568 | 528 | 1.823 | 6.885E-02 |
| Sum Carboxylic Acids | (Intercept) | 60.708 | 7.488 | 528 | 8.107 | 3.642E-15 |
|  | sum_carboxylic_log2 | 0.051 | 1.035 | 170 | 0.049 | 9.608E-01 |
|  | bSpline(visit_num, knots = 1, degree = 1)1 | -15.363 | 1.643 | 528 | -9.353 | 2.414E-19 |
|  | bSpline(visit_num, knots = 1, degree = 1)2 | -8.064 | 2.551 | 528 | -3.161 | 1.663E-03 |
|  | race_binaryOther | 5.032 | 1.441 | 170 | 3.492 | 6.117E-04 |
|  | sexFemale | -1.075 | 1.509 | 170 | -0.712 | 4.773E-01 |
|  | site_binary | -3.758 | 1.327 | 170 | -2.832 | 5.180E-03 |
|  | ageyrs | -0.328 | 0.410 | 528 | -0.801 | 4.235E-01 |
|  | parents_income25000-74999 | -3.472 | 1.461 | 170 | -2.376 | 1.864E-02 |
|  | parents_income75000 or more | -5.991 | 1.736 | 170 | -3.451 | 7.056E-04 |
|  | sum_carboxylic_log2:bSpline(visit_num, knots = 1, degree = 1)1 | -0.123 | 0.841 | 528 | -0.146 | 8.836E-01 |
|  | sum_carboxylic_log2:bSpline(visit_num, knots = 1, degree = 1)2 | -0.801 | 0.772 | 528 | -1.037 | 3.001E-01 |

Table S7. Linear mixed model outputs for each log_2_-PFAS congener on percent weight loss.

|  | Term | Value | Std.Error | DF | t-value | p-value |
| --- | --- | --- | --- | --- | --- | --- |
| PFOS | (Intercept) | 9.119 | 6.998 | 534 | 1.303 | 1.931E-01 |
|  | total_pfos_targeted_plasma_log2 | 0.124 | 1.072 | 170 | 0.116 | 9.078E-01 |
|  | bSpline(visit_num, knots = 1, degree = 1)1 | -30.369 | 3.186 | 534 | -9.533 | 5.364E-20 |
|  | bSpline(visit_num, knots = 1, degree = 1)2 | -22.683 | 3.425 | 534 | -6.624 | 8.562E-11 |
|  | race_binaryOther | 3.424 | 1.266 | 170 | 2.705 | 7.533E-03 |
|  | sexFemale | 1.648 | 1.334 | 170 | 1.236 | 2.182E-01 |
|  | site_binary | -2.586 | 1.159 | 170 | -2.231 | 2.696E-02 |
|  | ageyrs | -0.753 | 0.369 | 534 | -2.042 | 4.167E-02 |
|  | parents_income25000-74999 | 0.123 | 1.304 | 170 | 0.095 | 9.247E-01 |
|  | parents_income75000 or more | -1.160 | 1.536 | 170 | -0.755 | 4.514E-01 |
|  | total_pfos_targeted_plasma_log2:bSpline(visit_num, knots = 1, degree = 1)1 | 0.299 | 1.365 | 534 | 0.219 | 8.268E-01 |
|  | total_pfos_targeted_plasma_log2:bSpline(visit_num, knots = 1, degree = 1)2 | 2.542 | 1.234 | 534 | 2.060 | 3.985E-02 |
| PFHxS | (Intercept) | 7.846 | 6.674 | 534 | 1.176 | 2.403E-01 |
|  | pf_hx_s_targeted_plasma_log2 | 0.370 | 0.740 | 170 | 0.500 | 6.180E-01 |
|  | bSpline(visit_num, knots = 1, degree = 1)1 | -30.428 | 1.383 | 534 | -21.995 | 8.080E-77 |
|  | bSpline(visit_num, knots = 1, degree = 1)2 | -19.254 | 2.221 | 534 | -8.670 | 5.208E-17 |
|  | race_binaryOther | 3.516 | 1.253 | 170 | 2.807 | 5.585E-03 |
|  | sexFemale | 1.721 | 1.310 | 170 | 1.314 | 1.905E-01 |
|  | site_binary | -2.777 | 1.147 | 170 | -2.421 | 1.654E-02 |
|  | ageyrs | -0.681 | 0.363 | 534 | -1.876 | 6.122E-02 |
|  | parents_income25000-74999 | 0.067 | 1.275 | 170 | 0.052 | 9.582E-01 |
|  | parents_income75000 or more | -1.178 | 1.488 | 170 | -0.792 | 4.297E-01 |
|  | pf_hx_s_targeted_plasma_log2:bSpline(visit_num, knots = 1, degree = 1)1 | 0.727 | 0.949 | 534 | 0.766 | 4.438E-01 |
|  | pf_hx_s_targeted_plasma_log2:bSpline(visit_num, knots = 1, degree = 1)2 | 1.956 | 0.869 | 534 | 2.250 | 2.484E-02 |
| PFHpS | (Intercept) | 11.676 | 7.367 | 534 | 1.585 | 1.136E-01 |
|  | pf_hp_s_targeted_plasma_log2 | 0.647 | 1.055 | 170 | 0.614 | 5.404E-01 |
|  | bSpline(visit_num, knots = 1, degree = 1)1 | -30.335 | 3.547 | 534 | -8.552 | 1.278E-16 |
|  | bSpline(visit_num, knots = 1, degree = 1)2 | -9.682 | 3.697 | 534 | -2.619 | 9.077E-03 |
|  | race_binaryOther | 3.827 | 1.289 | 170 | 2.968 | 3.429E-03 |
|  | sexFemale | 1.933 | 1.346 | 170 | 1.436 | 1.529E-01 |
|  | site_binary | -2.546 | 1.154 | 170 | -2.205 | 2.880E-02 |
|  | ageyrs | -0.807 | 0.369 | 534 | -2.188 | 2.910E-02 |
|  | parents_income25000-74999 | 0.013 | 1.298 | 170 | 0.010 | 9.921E-01 |
|  | parents_income75000 or more | -1.359 | 1.532 | 170 | -0.887 | 3.764E-01 |
|  | pf_hp_s_targeted_plasma_log2:bSpline(visit_num, knots = 1, degree = 1)1 | -0.282 | 1.337 | 534 | -0.211 | 8.328E-01 |
|  | pf_hp_s_targeted_plasma_log2:bSpline(visit_num, knots = 1, degree = 1)2 | 2.857 | 1.204 | 534 | 2.374 | 1.797E-02 |
| Sum Sulfonic Acids | (Intercept) | 8.242 | 7.202 | 534 | 1.145 | 2.529E-01 |
|  | sum_sulfonic_log2 | 0.322 | 1.030 | 170 | 0.313 | 7.548E-01 |
|  | bSpline(visit_num, knots = 1, degree = 1)1 | -31.993 | 3.816 | 534 | -8.385 | 4.548E-16 |
|  | bSpline(visit_num, knots = 1, degree = 1)2 | -25.275 | 3.922 | 534 | -6.445 | 2.592E-10 |
|  | race_binaryOther | 3.466 | 1.257 | 170 | 2.757 | 6.472E-03 |
|  | sexFemale | 1.788 | 1.323 | 170 | 1.351 | 1.785E-01 |
|  | site_binary | -2.724 | 1.152 | 170 | -2.363 | 1.924E-02 |
|  | ageyrs | -0.735 | 0.365 | 534 | -2.014 | 4.453E-02 |
|  | parents_income25000-74999 | -0.045 | 1.293 | 170 | -0.035 | 9.721E-01 |
|  | parents_income75000 or more | -1.416 | 1.524 | 170 | -0.929 | 3.542E-01 |
|  | sum_sulfonic_log2:bSpline(visit_num, knots = 1, degree = 1)1 | 0.816 | 1.314 | 534 | 0.621 | 5.351E-01 |
|  | sum_sulfonic_log2:bSpline(visit_num, knots = 1, degree = 1)2 | 2.894 | 1.191 | 534 | 2.430 | 1.543E-02 |
| PFOA | (Intercept) | 7.570 | 6.775 | 534 | 1.117 | 2.644E-01 |
|  | pfoa_targeted_plasma_log2 | 0.857 | 1.000 | 170 | 0.857 | 3.924E-01 |
|  | bSpline(visit_num, knots = 1, degree = 1)1 | -28.569 | 1.866 | 534 | -15.313 | 3.831E-44 |
|  | bSpline(visit_num, knots = 1, degree = 1)2 | -15.612 | 2.516 | 534 | -6.206 | 1.091E-09 |
|  | race_binaryOther | 3.334 | 1.271 | 170 | 2.624 | 9.472E-03 |
|  | sexFemale | 1.364 | 1.330 | 170 | 1.026 | 3.065E-01 |
|  | site_binary | -2.593 | 1.188 | 170 | -2.183 | 3.038E-02 |
|  | ageyrs | -0.705 | 0.371 | 534 | -1.900 | 5.791E-02 |
|  | parents_income25000-74999 | 0.443 | 1.291 | 170 | 0.343 | 7.317E-01 |
|  | parents_income75000 or more | -0.698 | 1.514 | 170 | -0.461 | 6.453E-01 |
|  | pfoa_targeted_plasma_log2:bSpline(visit_num, knots = 1, degree = 1)1 | -1.027 | 1.294 | 534 | -0.794 | 4.276E-01 |
|  | pfoa_targeted_plasma_log2:bSpline(visit_num, knots = 1, degree = 1)2 | -1.437 | 1.172 | 534 | -1.226 | 2.208E-01 |
| PFNA | (Intercept) | 9.318 | 6.782 | 534 | 1.374 | 1.700E-01 |
|  | pfna_targeted_plasma_log2 | 0.038 | 1.185 | 170 | 0.032 | 9.747E-01 |
|  | bSpline(visit_num, knots = 1, degree = 1)1 | -28.680 | 1.399 | 534 | -20.506 | 2.350E-69 |
|  | bSpline(visit_num, knots = 1, degree = 1)2 | -16.533 | 2.232 | 534 | -7.407 | 5.081E-13 |
|  | race_binaryOther | 3.312 | 1.265 | 170 | 2.619 | 9.616E-03 |
|  | sexFemale | 1.476 | 1.322 | 170 | 1.117 | 2.656E-01 |
|  | site_binary | -2.352 | 1.177 | 170 | -1.998 | 4.727E-02 |
|  | ageyrs | -0.748 | 0.369 | 534 | -2.030 | 4.287E-02 |
|  | parents_income25000-74999 | 0.246 | 1.291 | 170 | 0.190 | 8.494E-01 |
|  | parents_income75000 or more | -1.082 | 1.532 | 170 | -0.706 | 4.809E-01 |
|  | pfna_targeted_plasma_log2:bSpline(visit_num, knots = 1, degree = 1)1 | 1.860 | 1.556 | 534 | 1.195 | 2.325E-01 |
|  | pfna_targeted_plasma_log2:bSpline(visit_num, knots = 1, degree = 1)2 | 1.037 | 1.395 | 534 | 0.743 | 4.576E-01 |
| PFDA | (Intercept) | 7.050 | 7.499 | 534 | 0.940 | 3.476E-01 |
|  | pfda_targeted_plasma_log2 | -0.514 | 1.190 | 170 | -0.432 | 6.662E-01 |
|  | bSpline(visit_num, knots = 1, degree = 1)1 | -26.524 | 4.047 | 534 | -6.553 | 1.327E-10 |
|  | bSpline(visit_num, knots = 1, degree = 1)2 | -20.192 | 4.080 | 534 | -4.949 | 9.995E-07 |
|  | race_binaryOther | 3.365 | 1.274 | 170 | 2.642 | 9.021E-03 |
|  | sexFemale | 1.339 | 1.322 | 170 | 1.013 | 3.126E-01 |
|  | site_binary | -2.661 | 1.170 | 170 | -2.274 | 2.420E-02 |
|  | ageyrs | -0.694 | 0.368 | 534 | -1.885 | 6.002E-02 |
|  | parents_income25000-74999 | 0.549 | 1.302 | 170 | 0.422 | 6.735E-01 |
|  | parents_income75000 or more | -0.538 | 1.521 | 170 | -0.354 | 7.241E-01 |
|  | pfda_targeted_plasma_log2:bSpline(visit_num, knots = 1, degree = 1)1 | 1.275 | 1.520 | 534 | 0.839 | 4.019E-01 |
|  | pfda_targeted_plasma_log2:bSpline(visit_num, knots = 1, degree = 1)2 | -1.108 | 1.369 | 534 | -0.809 | 4.187E-01 |
| PFHpA | (Intercept) | 9.480 | 6.940 | 534 | 1.366 | 1.725E-01 |
|  | pf_hp_a_targeted_plasma_log2 | 0.204 | 0.578 | 170 | 0.353 | 7.248E-01 |
|  | bSpline(visit_num, knots = 1, degree = 1)1 | -31.379 | 2.881 | 534 | -10.893 | 4.326E-25 |
|  | bSpline(visit_num, knots = 1, degree = 1)2 | -20.771 | 3.163 | 534 | -6.566 | 1.226E-10 |
|  | race_binaryOther | 3.284 | 1.270 | 170 | 2.587 | 1.053E-02 |
|  | sexFemale | 1.255 | 1.329 | 170 | 0.944 | 3.464E-01 |
|  | site_binary | -2.576 | 1.161 | 170 | -2.218 | 2.790E-02 |
|  | ageyrs | -0.716 | 0.369 | 534 | -1.942 | 5.270E-02 |
|  | parents_income25000-74999 | 0.588 | 1.301 | 170 | 0.452 | 6.522E-01 |
|  | parents_income75000 or more | -0.520 | 1.516 | 170 | -0.343 | 7.323E-01 |
|  | pf_hp_a_targeted_plasma_log2:bSpline(visit_num, knots = 1, degree = 1)1 | -0.457 | 0.762 | 534 | -0.600 | 5.487E-01 |
|  | pf_hp_a_targeted_plasma_log2:bSpline(visit_num, knots = 1, degree = 1)2 | -1.011 | 0.677 | 534 | -1.494 | 1.357E-01 |
| PFUnDA | (Intercept) | 6.145 | 8.026 | 534 | 0.766 | 4.443E-01 |
|  | pf_un_da_targeted_plasma_log2 | -0.781 | 0.893 | 170 | -0.874 | 3.831E-01 |
|  | bSpline(visit_num, knots = 1, degree = 1)1 | -21.614 | 4.727 | 534 | -4.572 | 6.006E-06 |
|  | bSpline(visit_num, knots = 1, degree = 1)2 | -9.965 | 4.672 | 534 | -2.133 | 3.340E-02 |
|  | race_binaryOther | 3.133 | 1.306 | 170 | 2.399 | 1.754E-02 |
|  | sexFemale | 1.265 | 1.322 | 170 | 0.957 | 3.401E-01 |
|  | site_binary | -2.461 | 1.176 | 170 | -2.093 | 3.788E-02 |
|  | ageyrs | -0.743 | 0.370 | 534 | -2.005 | 4.542E-02 |
|  | parents_income25000-74999 | 0.295 | 1.299 | 170 | 0.227 | 8.208E-01 |
|  | parents_income75000 or more | -0.840 | 1.509 | 170 | -0.557 | 5.785E-01 |
|  | pf_un_da_targeted_plasma_log2:bSpline(visit_num, knots = 1, degree = 1)1 | 1.960 | 1.108 | 534 | 1.769 | 7.739E-02 |
|  | pf_un_da_targeted_plasma_log2:bSpline(visit_num, knots = 1, degree = 1)2 | 1.720 | 1.002 | 534 | 1.717 | 8.664E-02 |
| Sum Carboxylic Acids | (Intercept) | 7.405 | 6.895 | 534 | 1.074 | 2.833E-01 |
|  | sum_carboxylic_log2 | 0.768 | 1.131 | 170 | 0.679 | 4.984E-01 |
|  | bSpline(visit_num, knots = 1, degree = 1)1 | -28.911 | 2.826 | 534 | -10.231 | 1.486E-22 |
|  | bSpline(visit_num, knots = 1, degree = 1)2 | -15.044 | 3.144 | 534 | -4.785 | 2.213E-06 |
|  | race_binaryOther | 3.343 | 1.270 | 170 | 2.633 | 9.237E-03 |
|  | sexFemale | 1.396 | 1.330 | 170 | 1.050 | 2.953E-01 |
|  | site_binary | -2.616 | 1.171 | 170 | -2.233 | 2.682E-02 |
|  | ageyrs | -0.715 | 0.372 | 534 | -1.924 | 5.491E-02 |
|  | parents_income25000-74999 | 0.406 | 1.292 | 170 | 0.314 | 7.540E-01 |
|  | parents_income75000 or more | -0.768 | 1.525 | 170 | -0.504 | 6.150E-01 |
|  | sum_carboxylic_log2:bSpline(visit_num, knots = 1, degree = 1)1 | -0.482 | 1.488 | 534 | -0.324 | 7.460E-01 |
|  | sum_carboxylic_log2:bSpline(visit_num, knots = 1, degree = 1)2 | -1.254 | 1.329 | 534 | -0.944 | 3.456E-01 |

Table S8. Linear mixed model outputs for each log_2_-PFAS congener on waist circumference.

|  | Term | Value | Std.Error | DF | t-value | p-value |
| --- | --- | --- | --- | --- | --- | --- |
| PFOS | (Intercept) | 144.141 | 14.595 | 487 | 9.876 | 4.330E-21 |
|  | total_pfos_targeted_plasma_log2 | 1.826 | 1.904 | 170 | 0.959 | 3.387E-01 |
|  | bSpline(visit_num, knots = 1, degree = 1)1 | -27.716 | 3.797 | 487 | -7.300 | 1.178E-12 |
|  | bSpline(visit_num, knots = 1, degree = 1)2 | -29.481 | 5.335 | 487 | -5.526 | 5.355E-08 |
|  | race_binaryOther | 7.831 | 2.746 | 170 | 2.852 | 4.884E-03 |
|  | sexFemale | -7.742 | 2.880 | 170 | -2.688 | 7.895E-03 |
|  | site_binary | -5.837 | 2.509 | 170 | -2.326 | 2.118E-02 |
|  | ageyrs | 0.021 | 0.786 | 487 | 0.027 | 9.789E-01 |
|  | parents_income25000-74999 | -5.881 | 2.815 | 170 | -2.089 | 3.820E-02 |
|  | parents_income75000 or more | -12.045 | 3.330 | 170 | -3.617 | 3.930E-04 |
|  | total_pfos_targeted_plasma_log2:bSpline(visit_num, knots = 1, degree = 1)1 | -1.257 | 1.600 | 487 | -0.785 | 4.326E-01 |
|  | total_pfos_targeted_plasma_log2:bSpline(visit_num, knots = 1, degree = 1)2 | 1.579 | 1.519 | 487 | 1.040 | 2.990E-01 |
| PFHxS | (Intercept) | 145.832 | 14.307 | 487 | 10.193 | 3.021E-22 |
|  | pf_hx_s_targeted_plasma_log2 | 0.657 | 1.302 | 170 | 0.504 | 6.147E-01 |
|  | bSpline(visit_num, knots = 1, degree = 1)1 | -30.876 | 1.770 | 487 | -17.444 | 2.699E-53 |
|  | bSpline(visit_num, knots = 1, degree = 1)2 | -29.277 | 4.293 | 487 | -6.820 | 2.699E-11 |
|  | race_binaryOther | 7.816 | 2.745 | 170 | 2.848 | 4.948E-03 |
|  | sexFemale | -7.753 | 2.857 | 170 | -2.713 | 7.346E-03 |
|  | site_binary | -6.094 | 2.508 | 170 | -2.430 | 1.616E-02 |
|  | ageyrs | 0.122 | 0.781 | 487 | 0.157 | 8.756E-01 |
|  | parents_income25000-74999 | -5.749 | 2.783 | 170 | -2.066 | 4.033E-02 |
|  | parents_income75000 or more | -11.794 | 3.262 | 170 | -3.616 | 3.946E-04 |
|  | pf_hx_s_targeted_plasma_log2:bSpline(visit_num, knots = 1, degree = 1)1 | 0.273 | 1.124 | 487 | 0.243 | 8.082E-01 |
|  | pf_hx_s_targeted_plasma_log2:bSpline(visit_num, knots = 1, degree = 1)2 | 2.971 | 1.101 | 487 | 2.698 | 7.219E-03 |
| PFHpS | (Intercept) | 158.957 | 15.346 | 487 | 10.358 | 7.430E-23 |
|  | pf_hp_s_targeted_plasma_log2 | 3.555 | 1.882 | 170 | 1.889 | 6.057E-02 |
|  | bSpline(visit_num, knots = 1, degree = 1)1 | -36.242 | 4.238 | 487 | -8.551 | 1.593E-16 |
|  | bSpline(visit_num, knots = 1, degree = 1)2 | -19.580 | 5.728 | 487 | -3.418 | 6.831E-04 |
|  | race_binaryOther | 8.846 | 2.782 | 170 | 3.179 | 1.756E-03 |
|  | sexFemale | -7.005 | 2.895 | 170 | -2.419 | 1.661E-02 |
|  | site_binary | -5.660 | 2.490 | 170 | -2.273 | 2.430E-02 |
|  | ageyrs | -0.125 | 0.783 | 487 | -0.160 | 8.729E-01 |
|  | parents_income25000-74999 | -6.238 | 2.789 | 170 | -2.237 | 2.659E-02 |
|  | parents_income75000 or more | -12.689 | 3.308 | 170 | -3.836 | 1.760E-04 |
|  | pf_hp_s_targeted_plasma_log2:bSpline(visit_num, knots = 1, degree = 1)1 | -2.365 | 1.576 | 487 | -1.501 | 1.341E-01 |
|  | pf_hp_s_targeted_plasma_log2:bSpline(visit_num, knots = 1, degree = 1)2 | 2.261 | 1.513 | 487 | 1.494 | 1.359E-01 |
| Sum Sulfonic Acids | (Intercept) | 142.806 | 14.972 | 487 | 9.538 | 6.963E-20 |
|  | sum_sulfonic_log2 | 1.747 | 1.827 | 170 | 0.956 | 3.402E-01 |
|  | bSpline(visit_num, knots = 1, degree = 1)1 | -28.995 | 4.531 | 487 | -6.399 | 3.683E-10 |
|  | bSpline(visit_num, knots = 1, degree = 1)2 | -34.214 | 5.877 | 487 | -5.822 | 1.059E-08 |
|  | race_binaryOther | 7.841 | 2.739 | 170 | 2.863 | 4.732E-03 |
|  | sexFemale | -7.600 | 2.870 | 170 | -2.648 | 8.848E-03 |
|  | site_binary | -6.035 | 2.506 | 170 | -2.408 | 1.712E-02 |
|  | ageyrs | 0.054 | 0.781 | 487 | 0.070 | 9.445E-01 |
|  | parents_income25000-74999 | -6.043 | 2.806 | 170 | -2.154 | 3.266E-02 |
|  | parents_income75000 or more | -12.306 | 3.319 | 170 | -3.707 | 2.832E-04 |
|  | sum_sulfonic_log2:bSpline(visit_num, knots = 1, degree = 1)1 | -0.551 | 1.547 | 487 | -0.356 | 7.220E-01 |
|  | sum_sulfonic_log2:bSpline(visit_num, knots = 1, degree = 1)2 | 2.874 | 1.497 | 487 | 1.920 | 5.547E-02 |
| PFOA | (Intercept) | 144.879 | 14.303 | 487 | 10.129 | 5.203E-22 |
|  | pfoa_targeted_plasma_log2 | 2.345 | 1.759 | 170 | 1.333 | 1.843E-01 |
|  | bSpline(visit_num, knots = 1, degree = 1)1 | -27.837 | 2.289 | 487 | -12.163 | 6.576E-30 |
|  | bSpline(visit_num, knots = 1, degree = 1)2 | -22.949 | 4.558 | 487 | -5.035 | 6.744E-07 |
|  | race_binaryOther | 7.731 | 2.752 | 170 | 2.809 | 5.556E-03 |
|  | sexFemale | -8.051 | 2.871 | 170 | -2.804 | 5.636E-03 |
|  | site_binary | -6.089 | 2.567 | 170 | -2.372 | 1.881E-02 |
|  | ageyrs | 0.052 | 0.789 | 487 | 0.066 | 9.470E-01 |
|  | parents_income25000-74999 | -5.403 | 2.784 | 170 | -1.941 | 5.393E-02 |
|  | parents_income75000 or more | -11.429 | 3.279 | 170 | -3.486 | 6.248E-04 |
|  | pfoa_targeted_plasma_log2:bSpline(visit_num, knots = 1, degree = 1)1 | -2.289 | 1.509 | 487 | -1.516 | 1.301E-01 |
|  | pfoa_targeted_plasma_log2:bSpline(visit_num, knots = 1, degree = 1)2 | -2.790 | 1.522 | 487 | -1.833 | 6.744E-02 |
| PFNA | (Intercept) | 146.008 | 14.452 | 487 | 10.103 | 6.486E-22 |
|  | pfna_targeted_plasma_log2 | -0.977 | 2.113 | 170 | -0.463 | 6.443E-01 |
|  | bSpline(visit_num, knots = 1, degree = 1)1 | -30.550 | 1.793 | 487 | -17.038 | 2.123E-51 |
|  | bSpline(visit_num, knots = 1, degree = 1)2 | -26.133 | 4.315 | 487 | -6.056 | 2.795E-09 |
|  | race_binaryOther | 7.707 | 2.753 | 170 | 2.800 | 5.706E-03 |
|  | sexFemale | -8.285 | 2.867 | 170 | -2.889 | 4.363E-03 |
|  | site_binary | -6.030 | 2.558 | 170 | -2.357 | 1.954E-02 |
|  | ageyrs | 0.117 | 0.788 | 487 | 0.149 | 8.819E-01 |
|  | parents_income25000-74999 | -5.213 | 2.796 | 170 | -1.865 | 6.394E-02 |
|  | parents_income75000 or more | -10.913 | 3.336 | 170 | -3.272 | 1.295E-03 |
|  | pfna_targeted_plasma_log2:bSpline(visit_num, knots = 1, degree = 1)1 | 0.087 | 1.829 | 487 | 0.047 | 9.622E-01 |
|  | pfna_targeted_plasma_log2:bSpline(visit_num, knots = 1, degree = 1)2 | 0.748 | 1.683 | 487 | 0.444 | 6.571E-01 |
| PFDA | (Intercept) | 142.305 | 15.548 | 487 | 9.153 | 1.531E-18 |
|  | pfda_targeted_plasma_log2 | -1.400 | 2.103 | 170 | -0.666 | 5.064E-01 |
|  | bSpline(visit_num, knots = 1, degree = 1)1 | -32.170 | 4.767 | 487 | -6.748 | 4.263E-11 |
|  | bSpline(visit_num, knots = 1, degree = 1)2 | -30.464 | 5.922 | 487 | -5.145 | 3.892E-07 |
|  | race_binaryOther | 7.973 | 2.757 | 170 | 2.892 | 4.328E-03 |
|  | sexFemale | -8.327 | 2.852 | 170 | -2.919 | 3.982E-03 |
|  | site_binary | -6.156 | 2.529 | 170 | -2.434 | 1.597E-02 |
|  | ageyrs | 0.144 | 0.784 | 487 | 0.183 | 8.546E-01 |
|  | parents_income25000-74999 | -4.888 | 2.808 | 170 | -1.741 | 8.350E-02 |
|  | parents_income75000 or more | -10.566 | 3.298 | 170 | -3.204 | 1.620E-03 |
|  | pfda_targeted_plasma_log2:bSpline(visit_num, knots = 1, degree = 1)1 | -0.599 | 1.766 | 487 | -0.339 | 7.346E-01 |
|  | pfda_targeted_plasma_log2:bSpline(visit_num, knots = 1, degree = 1)2 | -1.511 | 1.650 | 487 | -0.916 | 3.603E-01 |
| PFHpA | (Intercept) | 155.358 | 14.457 | 487 | 10.746 | 2.572E-24 |
|  | pf_hp_a_targeted_plasma_log2 | 2.829 | 1.008 | 170 | 2.806 | 5.604E-03 |
|  | bSpline(visit_num, knots = 1, degree = 1)1 | -37.489 | 3.453 | 487 | -10.858 | 9.667E-25 |
|  | bSpline(visit_num, knots = 1, degree = 1)2 | -35.143 | 5.128 | 487 | -6.853 | 2.188E-11 |
|  | race_binaryOther | 7.997 | 2.736 | 170 | 2.923 | 3.944E-03 |
|  | sexFemale | -7.626 | 2.855 | 170 | -2.671 | 8.299E-03 |
|  | site_binary | -5.828 | 2.497 | 170 | -2.334 | 2.077E-02 |
|  | ageyrs | 0.172 | 0.780 | 487 | 0.221 | 8.252E-01 |
|  | parents_income25000-74999 | -5.967 | 2.789 | 170 | -2.140 | 3.382E-02 |
|  | parents_income75000 or more | -12.136 | 3.270 | 170 | -3.711 | 2.795E-04 |
|  | pf_hp_a_targeted_plasma_log2:bSpline(visit_num, knots = 1, degree = 1)1 | -1.955 | 0.893 | 487 | -2.190 | 2.903E-02 |
|  | pf_hp_a_targeted_plasma_log2:bSpline(visit_num, knots = 1, degree = 1)2 | -2.398 | 0.846 | 487 | -2.836 | 4.763E-03 |
| PFUnDA | (Intercept) | 139.349 | 16.563 | 487 | 8.413 | 4.464E-16 |
|  | pf_un_da_targeted_plasma_log2 | -1.619 | 1.597 | 170 | -1.014 | 3.119E-01 |
|  | bSpline(visit_num, knots = 1, degree = 1)1 | -25.883 | 5.572 | 487 | -4.645 | 4.377E-06 |
|  | bSpline(visit_num, knots = 1, degree = 1)2 | -19.424 | 6.529 | 487 | -2.975 | 3.075E-03 |
|  | race_binaryOther | 8.065 | 2.835 | 170 | 2.844 | 4.997E-03 |
|  | sexFemale | -8.123 | 2.861 | 170 | -2.839 | 5.081E-03 |
|  | site_binary | -6.084 | 2.549 | 170 | -2.386 | 1.812E-02 |
|  | ageyrs | 0.132 | 0.790 | 487 | 0.168 | 8.669E-01 |
|  | parents_income25000-74999 | -5.146 | 2.811 | 170 | -1.831 | 6.887E-02 |
|  | parents_income75000 or more | -11.022 | 3.279 | 170 | -3.362 | 9.558E-04 |
|  | pf_un_da_targeted_plasma_log2:bSpline(visit_num, knots = 1, degree = 1)1 | 1.136 | 1.289 | 487 | 0.881 | 3.787E-01 |
|  | pf_un_da_targeted_plasma_log2:bSpline(visit_num, knots = 1, degree = 1)2 | 1.740 | 1.210 | 487 | 1.438 | 1.510E-01 |
| Sum Carboxylic Acids | (Intercept) | 143.517 | 14.435 | 487 | 9.943 | 2.492E-21 |
|  | sum_carboxylic_log2 | 2.111 | 1.989 | 170 | 1.061 | 2.901E-01 |
|  | bSpline(visit_num, knots = 1, degree = 1)1 | -26.301 | 3.378 | 487 | -7.786 | 4.192E-14 |
|  | bSpline(visit_num, knots = 1, degree = 1)2 | -21.506 | 5.133 | 487 | -4.190 | 3.314E-05 |
|  | race_binaryOther | 7.722 | 2.752 | 170 | 2.806 | 5.608E-03 |
|  | sexFemale | -8.098 | 2.874 | 170 | -2.817 | 5.415E-03 |
|  | site_binary | -5.909 | 2.533 | 170 | -2.332 | 2.085E-02 |
|  | ageyrs | 0.073 | 0.791 | 487 | 0.093 | 9.261E-01 |
|  | parents_income25000-74999 | -5.387 | 2.788 | 170 | -1.932 | 5.503E-02 |
|  | parents_income75000 or more | -11.394 | 3.305 | 170 | -3.447 | 7.134E-04 |
|  | sum_carboxylic_log2:bSpline(visit_num, knots = 1, degree = 1)1 | -2.416 | 1.740 | 487 | -1.389 | 1.656E-01 |
|  | sum_carboxylic_log2:bSpline(visit_num, knots = 1, degree = 1)2 | -2.730 | 1.690 | 487 | -1.615 | 1.070E-01 |

Table S9. Results from quantile g-computation for each PFAS mixture and outcome five years after surgery.

| Outcome (5 Years) | Ψ | 95% CI | P-value | Mixture | Model |
| --- | --- | --- | --- | --- | --- |
| BMI (kg/m^2^) | 0.43 | -2.30, 3.16 | 0.76 | All PFAS | Model 1 |
| Waist Circumference (cm) | 1.86 | -3.51, 7.24 | 0.50 |  |  |
| % Weight Loss | 1.16 | -2.31, 4.64 | 0.51 |  |  |
| BMI (kg/m^2^) | 0.089 | -2.72, 2.90 | 0.95 |  | Model 2 |
| Waist Circumference (cm) | 1.60 | -3.73, 6.92 | 0.56 |  |  |
| % Weight Loss | 0.98 | -2.63, 4.59 | 0.60 |  |  |
| BMI (kg/m^2^) | -1.09 | -3.62, 1.45 | 0.40 | Carboxylic Acids | Model 1 |
| Waist Circumference (cm) | -1.99 | -7.11, 3.13 | 0.45 |  |  |
| % Weight Loss | -0.89 | -4.06, 2.29 | 0.58 |  |  |
| BMI (kg/m^2^) | -1.22 | -3.80, 1.35 | 0.35 |  | Model 2 |
| Waist Circumference (cm) | -1.40 | -6.43, 3.63 | 0.59 |  |  |
| % Weight Loss | -1.03 | -4.26, 2.21 | 0.54 |  |  |
| BMI (kg/m^2^) | 1.94 | -0.076, 3.96 | 0.061 | Sulfonic Acids | Model 1 |
| Waist Circumference (cm) | 4.41 | 0.23, 8.59 | 0.041 |  |  |
| % Weight Loss | 2.51 | -0.040, 5.06 | 0.056 |  |  |
| BMI (kg/m^2^) | 1.77 | -0.27, 3.81 | 0.092 |  | Model 2 |
| Waist Circumference (cm) | 3.81 | -0.27, 7.89 | 0.070 |  |  |
| % Weight Loss | 2.40 | -0.20, 5.00 | 0.073 |  |  |
| Notes: Model 1 adjusted for age, sex, race, study site, and parents’ income at baseline. Model 2 adjusted for the same covariates as Model 1, as well as for HOMA-IR at baseline. | | | | | |
